# Comprehensive *in silico *prediction and analysis of chlamydial outer membrane proteins reflects evolution and life style of the *Chlamydiae*

**DOI:** 10.1186/1471-2164-10-634

**Published:** 2009-12-29

**Authors:** Eva Heinz, Patrick Tischler, Thomas Rattei, Garry Myers, Michael Wagner, Matthias Horn

**Affiliations:** 1 Department of Microbial Ecology, Faculty of Life Sciences, University of Vienna, Vienna, Austria; 2 Department of Computational Systems Biology, Faculty of Life Sciences, University of Vienna, Vienna, Austria; 3 Genome oriented Bioinformatics, Technische Universität München, Freising, Germany; 4 Institute for Genome Science, University of Maryland, Baltimore, MD, USA

## Abstract

**Background:**

Chlamydiae are obligate intracellular bacteria comprising some of the most important bacterial pathogens of animals and humans. Although chlamydial outer membrane proteins play a key role for attachment to and entry into host cells, only few have been described so far. We developed a comprehensive, multiphasic *in silico *approach, including the calculation of clusters of orthologues, to predict outer membrane proteins using conservative criteria. We tested this approach using *Escherichia coli *(positive control) and *Bacillus subtilis *(negative control), and applied it to five chlamydial species; *Chlamydia trachomatis*, *Chlamydia muridarum*, *Chlamydia *(a.k.a. *Chlamydophila*) *pneumoniae*, *Chlamydia *(a.k.a. *Chlamydophila*) *caviae*, and *Protochlamydia amoebophila*.

**Results:**

In total, 312 chlamydial outer membrane proteins and lipoproteins in 88 orthologous clusters were identified, including 238 proteins not previously recognized to be located in the outer membrane. Analysis of their taxonomic distribution revealed an evolutionary conservation among *Chlamydiae*, *Verrucomicrobia*, *Lentisphaerae *and *Planctomycetes *as well as lifestyle-dependent conservation of the chlamydial outer membrane protein composition.

**Conclusion:**

This analysis suggested a correlation between the outer membrane protein composition and the host range of chlamydiae and revealed a common set of outer membrane proteins shared by these intracellular bacteria. The collection of predicted chlamydial outer membrane proteins is available at the online database pCOMP http://www.microbial-ecology.net/pcomp and might provide future guidance in the quest for anti-chlamydial vaccines.

## Background

The phylum *Chlamydiae *is a unique group of evolutionary well separated, intracellular bacteria that comprises some of the most important bacterial pathogens of humans and animals. *Chlamydia trachomatis *is the world's leading cause of preventable blindness [1] and with over 90 million new cases each year the most frequently sexually transmitted bacterial infection, which can lead to pelvic inflammatory disease, Fallopian tube obstruction, potentially life-threatening ectopic pregnancy, infertility and subfertility [2]. *Chlamydia *(a.k.a. *Chlamydophila*) *pneumoniae *is a causative agent of community acquired pneumonia and might be associated with several chronic diseases such as atherosclerosis [3,4].

All recognized chlamydial pathogens form a small group of closely related bacteria constituting the family *Chlamydiaceae *within the phylum *Chlamydiae*. In addition, seven new families, the *Clavochlamydiaceae*, *Criblamydiaceae*, *Parachlamydiaceae, Piscichlamydiaceae, Rhabdochlamydiaceae, Simkaniaceae*, and *Waddliaceae *were recently described within this phylum [5], revealing a previously underestimated diversity of these elusive microorganisms, which is also represented by their extremely broad host spectrum. More than 60 host species are known, ranging from mammals, marsupials, birds, reptiles, amphibians and fish to insects, crustaceans, molluscs and protozoa [6]. Moreover, evidence exists that the recognized diversity and host range represent only the tip of the iceberg and that chlamydiae are ubiquitous [6].

A hallmark of all chlamydiae is their obligate intracellular lifestyle and a developmental cycle consisting of morphologically and physiologically distinct stages. The chlamydial elementary body (EB) is the infectious form that is metabolically inert and can persist in the environment. After infection of a eukaryotic host cell the EB transforms into a reticulate body (RB), which is metabolically active and divides by binary fission within a host-derived vacuole termed inclusion [7-9]. Following the replicative phase RBs differentiate to EBs, that are released into the environment either by lysis of the host cell or exocytosis [10] and a new infection cycle begins.

The crucial step of attachment to and entry into the host cell is mediated by the chlamydial cell envelope, which is one of the most inimitable features of this unique group of microbes. The possible lack of peptidoglycan in chlamydiae is a subject of ongoing discussion [11], but the difficulties of detecting it clearly support the hypothesis that chlamydiae lack peptidoglycan as main structural and stabilizing element of the cell envelope, which is believed to be substituted by the chlamydial outer membrane complex as a structure-giving component. This assemblage of proteins consists of two cysteine-rich proteins (OmcA and OmcB) and the major outer membrane protein (MOMP, OmpA [12]). The only other group of well-described chlamydial outer membrane proteins are the polymorphic membrane proteins (Pmps, [13-15]) a family of autotransporters. Some Pmps play a role in the attachment to the host cell [16] and are possibly contributing to tissue specificity of different *C. trachomatis *disease groups [17,18].

However, despite the importance of outer membrane proteins in the initial steps of host cell invasion, current knowledge about the key players in this process is still scarce. This reflects the general challenges in the analysis of chlamydiae as there are currently no means to genetically manipulate them and hence to characterize protein function by classic genetic methods [19]. Additionally, hardly any studies attempted to obtain a comprehensive picture of the outer membrane components by a systematic approach [20,21]. Among sequenced *Chlamydiaceae *genomes, between 31 (*C. trachomatis*) and 40 proteins (*C. pneumoniae*) are currently annotated as chlamydial outer membrane proteins with most of them belonging to the Pmp family or being classified as lipoproteins. This is a surprisingly low number when compared to other intracellular bacteria with a similar life style and genome size such as *Anaplasma marginale*, which has a genome of 1.1 Mb and also replicates in a membrane-enclosed compartment within its host cells. Compared to the *Chlamydiaceae *with their 1-1.2 Mb genomes, up to twice as many (62) outer membrane proteins could be identified in the *A. marginale *genome [22]. Furthermore, genomic analysis of the environmental counterpart of the *Chlamydiaceae*, the amoeba symbiont *Protochlamydia amoebophila *(a member of the *Parachlamydiaceae*) [23], revealed only homologues of the cysteine-rich proteins OmcA and OmcB, but no homologues of other main components of the chlamydial outer membrane complex (MOMP or Pmps). The apparent absence of these dominant proteins and the unexpectedly low number of other annotated outer membrane proteins in *P. amoebophila *further illustrate our general lack of knowledge about the outer membrane and suggest that its main components are unique and yet unknown proteins.

**Figure 1:**
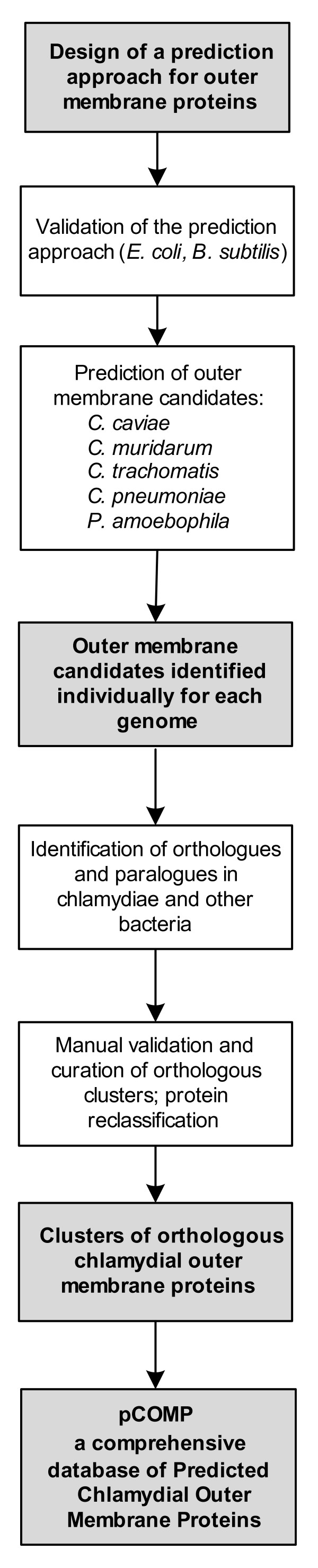
**The approach to identify chlamydial outer membrane proteins applied in this study**. The individual steps used to identify chlamydial outer membrane proteins are indicated. Further details on the prediction approach on the level of individual genomes are given in Figure 2. Protein reclassification is described in more detail in additional file 1: Supporting Information, Figure S1. Annotated prediction results are available in the pCOMP database http://www.microbial-ecology.net/pcomp and in additional file 1: Supporting Information, Table S3.

To close this gap of knowledge, we have developed an *in silico *approach for the identification of yet unknown chlamydial outer membrane proteins (Figure 1). An extensive combination of different prediction programs and manual curation steps using conservative criteria were applied to determine a comprehensive and reliable set of chlamydial outer membrane proteins. This approach was tested with the well characterized proteomes of *Escherichia coli *and *Bacillus subtilis *and subsequently used to predict the outer membrane proteins of five chlamydial species, *Chlamydia trachomatis *D/UW3/CX, *Chlamydia muridarum *Nigg, *Chlamydia *(a.k.a. *Chlamydophila*) *pneumoniae *AR39, *Chlamydia *(a.k.a. *Chlamydophila*) *caviae *GPIC, and the amoeba symbiont *P. amoebophila *UWE25 (Figure 1) [23-26]. In addition, the taxonomic distribution of the identified outer membrane proteins was analysed to illuminate their conservation throughout the chlamydiae and among representatives of all other bacterial lineages.

Our *in silico *analysis predicted 312 outer membrane proteins for the five chlamydial species including more than 100 novel outer membrane proteins of the *Chlamydiaceae *despite our conservative approach. A database available online at http://www.microbial-ecology.net/pcomp was set up and provides access to all predicted outer membrane proteins including details on taxonomic distribution, results of the individual programs used, and links to experimental evidence for their location if available. Taken together, we present a comprehensive and curated set of candidate outer membrane proteins of the *Chlamydiae*. As outer membrane proteins are preferred targets for anti-bacterial vaccines, these data can provide guidance for future development of anti-chlamydial immunization strategies.

## Results and Discussion

Well-characterized outer membrane proteins are rare in the scientific literature compared to all other proteins as they are experimentally elusive due to their physicochemical properties. Furthermore, the *in silico *identification of outer membrane proteins is challenging as their main three-dimensional fold, the beta-barrel, is very difficult to predict based on the amino acid sequence, if no closely related protein with known 3D structure is available. All 85 structural entries of a resolved transmembrane beta-barrel structure according to the RCSB protein data bank [27] are from members of the *Proteobacteria *and consequently, available outer membrane predictors have been trained mostly on proteobacterial sequences. As chlamydiae are a unique group of microorganisms only distantly related to the *Proteobacteria*, chlamydial outer membrane proteins pose an even greater challenge to prediction programs than proteins from organisms more closely related to the training set. To account for the difficulties in predicting a reliable set of chlamydial outer membrane proteins, we developed a semi-automatic procedure comprising 10 different programs using various mathematical approaches and providing overlapping as well as complementary predictions.

### A multiphasic *in silico *approach to predict outer membrane proteins

**Figure 2:**
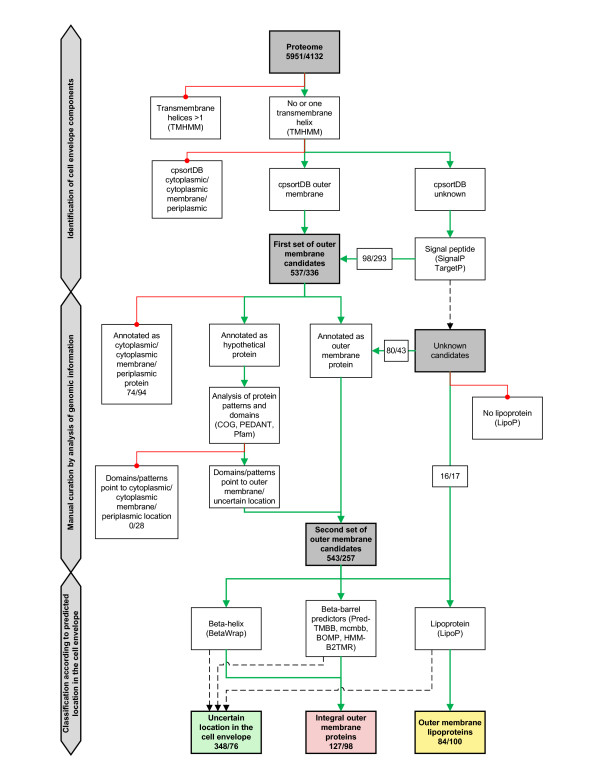
**Prediction of outer membrane proteins**. All proteins from the five chlamydial proteomes of *C. caviae *GPIC, *C. trachomatis *D/UW-3/CX, *C. muridarum *Nigg, *C. pneumoniae *AR39 and *P. amoebophila *UWE25 as well as *E. coli *K12 and *B. subtilis *subsp. *subtilis *were subjected to the prediction approach for the identification of outer membrane proteins. Solid green lines indicate subsets of proteins included in further analysis; solid red lines indicate protein subsets excluded from further analysis; dashed black lines indicate negative result obtained with the respective prediction programs. Total numbers of proteins for all five chlamydial organisms and *E. coli*, respectively, are shown.

The multiphasic outer membrane protein prediction approach designed in this study can be subdivided into three major steps. In the first step, the complete *in silico *proteome of the respective organism was screened for general features of proteins translocated across the cytoplasmic membrane. In a second, more rigorous step, the list was curated manually by taking into account the proteins' annotation, domain, motif and pattern information. The last step aimed at the identification of integral outer membrane proteins and outer membrane lipoproteins within this subset based on conserved structural features. Further details on the design of the prediction approach and the individual programs and thresholds used are provided in the Methods section and in Figure 2.

To test sensitivity and specificity of our prediction approach, we applied it to the *in silico *proteomes of *Escherichia coli *K12 and *Bacillus subtilis *subsp. *subtilis *[27,28], being extremely well-studied model organisms with extensive experimental data concerning protein function and location. Outer membrane protein candidates were predicted as described above and evaluated by a comparison with experimental data.

*B. subtilis *served as negative control for the prediction approach, since as a Gram-positive microorganism it lacks an outer membrane, and therefore all predicted outer membrane proteins can be considered false positives. From 4,105 proteins encoded in the *B. subtilis *genome, 31 were predicted as integral outer membrane proteins, resulting in a false positive rate of 0.73%. Ten of those proteins are experimentally verified cell wall associated proteins partly with a high pI (9-10) and a predicted signal peptide, which might explain their misclassification as integral outer membrane proteins. The remaining proteins are annotated as hypothetical proteins with unknown location. Prediction of outer membrane lipoproteins was not performed for *B. subtilis*, as the differentiation of outer membrane from cytoplasmic membrane lipoproteins is based on only one amino acid [28], which is only applicable for bacteria with a Gram-negative type cell wall.

The Gram-negative model organism *E. coli *served as positive control for our prediction approach, and 98 integral outer membrane proteins were predicted (see additional file 1: Supporting Information, Table S1). For the evaluation of these predictions, the *E. coli *knowledge base *Echo*LOCATION [29] was used, which provides comprehensive subcellular location analysis supported by experimental data for all *E. coli *proteins. For proteins without evidence for their location in *Echo*LOCATION a literature search was performed in order to include also very recent findings, resulting in a set of 45 experimentally confirmed integral outer membrane proteins. 42 of those 45 proteins (93%) were recognized by our prediction approach as outer membrane proteins. Three proteins (7%) were not identified and therefore represent false negatives. Only two proteins (4%) were false positives according to published data. Those two proteins showed no transmembrane helices, but a signal peptide and were predicted as beta-barrel proteins. In addition, 100 outer membrane lipoproteins were predicted, and published experimental data supports an outer membrane location of 71 proteins (93% of 76 experimentally verified outer membrane lipoproteins). According to published data no false positives and five false negatives (7%) were identified. The predicted outer membrane proteins of *E. coli *identified in this study are listed in Table S1 (see additional file 1: Supporting Information).

The achieved sensitivity (the fraction of correctly recognized outer membrane proteins) and specificity (the fraction of globular cytoplasmic proteins correctly recognized as non-outer membrane proteins) of the prediction approach developed in this study is better than the performance of individual beta-barrel prediction programs. Two of the programs used in this study, PRED-TMBB and B2TMR-HMM, were previously reported to show a sensitivity of 88% and 84%, and a specificity of 89% and 90%, respectively, with datasets consisting of well-described outer membrane or globular cytoplasmic proteins [30,31]. However, in a comparative analysis of several beta-barrel predictors, the sensitivity of these two programs was 70% (PRED-TMBB) and 75% (B2TMR-HMM), respectively, when applied to a smaller dataset containing only 20 beta barrel proteins known at the structural level [32]. An improvement of sensitivity to 90% was achieved by a consensus prediction using the five best performing programs [32]. The validation of the prediction approach designed in this study resulted in a sensitivity of 93% (7% false negatives) and a specificity of 96% (4% false positives) for experimentally verified proteins of *E. coli *and a specificity of 99% for *B. subtilis*. This shows that the multiphasic approach including manual curation steps used in this study achieves a higher sensitivity and specificity compared to individual or other consensus prediction methods.

For a comparison with an experimental data set obtained by a high-throughput proteomic study, we compared our analyses with a recent study focussing on the surface proteins of uropathogenic *E. coli *[33]. This study detected 102 proteins in outer membrane fractions analysed by LC/MS-MS. Twenty five of these proteins were designated as outer membrane proteins, and 36 proteins had an unknown location according to psort [34], which the authors used for prediction. All other detected proteins are likely contaminants including many ribosomal proteins, a common contaminant in outer membrane proteomic studies. A comparison with the highly similar proteins of *E. coli *K12 showed that out of the 25 detected proteins designated as outer membrane proteins, 24 were recognized correctly by our approach as outer membrane proteins (see additional file 1: Supporting Information, Table S1, S2, S3). The only protein not recognised as putative outer membrane protein in our analysis represents a false positive in the proteomic study as it functions in the periplasm to assist transport by outer membrane proteins [35]. Furthermore, 15 detected proteins categorized as unknown location were correctly recognized by our approach as outer membrane proteins. This high degree of overlap between the experimental data from this study and our prediction results further illustrates the high selectivity and sensitivity of our prediction approach.

### Prediction of chlamydial outer membrane proteins

Next the developed approach was deployed to predict outer membrane proteins in five chlamydial proteomes from two human pathogens (*C. trachomatis *D/UW3/CX, *C. pneumoniae *AR39), two animal pathogens (*C. muridarum *Nigg, *C. caviae *GPIC) and an amoeba symbiont (*P. amoebophila *UWE25; Figure 1; [23-26]). A detailed presentation of all predicted proteins is available as Tables S2, S3, and S4 (see additional file 1: Supporting Information). In total, 33 *C. trachomatis*, 34 *C. muridarum*, 49 *C. caviae*, 53 *C. pneumoniae *and 42 *P. amoebophila *predicted integral outer membrane proteins and predicted outer membrane lipoproteins could be identified (Table 1). A striking finding of this analysis was that in general more heterogeneous and less well-defined prediction results were obtained for chlamydial outer membrane proteins when compared to *E. coli*. For example, even the well-characterized chlamydial porin MOMP (a trimeric beta-barrel protein) never reaches prediction results as clear as most *E. coli *porins (see additional file 1: Supporting Information, Tables S1 and S2). This might not be surprising if one considers that prediction algorithms are generally optimized for proteobacterial proteins. Thus the high sensitivity of our prediction approach achieved for *E. coli *is certainly not valid for chlamydial proteins. We therefore reasoned that by identifying orthologous groups of proteins (Figure 1), uncertain prediction results (i.e. proteins classified as cell envelope components) can be resolved by taking into account the predictions of all orthologues, which should further improve the accuracy of the prediction.

**Table 1:** Summary of predicted outer membrane proteins.

Organism	Integral membrane protein	Outer membrane lipoproteins	Cell envelope component with uncertain prediction	Proteome
E. coli K12	98	100	76	4132

C. caviae GPIC*	29/**40**	20/**26**	60/**10**	1004

C. pneumoniae AR39*	33/**46**	20/**24**	54/**12**	1111

C. trachomatis D/UW-3/CX*	21/**35**	12/**20**	47/**1**	895

C. muridarum Nigg*	22/**37**	12/**22**	55/**4**	910

P. amoebophila UWE25*	22/**35**	20/**27**	132/**58**	2031

* Numbers of proteins obtained before/after orthologous cluster formation and reclassification are shown.

### Predicted chlamydial outer membrane proteins belong to 88 orthologous clusters

Clusters of orthologous proteins were constructed for all chlamydial proteins from bidirectional best FASTA hits using an empirically determined E-value and overlapping cut-off values. In total 1,911 clusters were found, from which 190 contained at least one chlamydial protein predicted either as integral outer membrane protein, as outer membrane lipoprotein, or as cell envelope component. As expected, a number of clusters comprised proteins with different prediction results, confirming our previous observation that the structure of chlamydial proteins is difficult to predict and therefore often leads to ambiguous results in signal peptide, transmembrane helix or general location prediction. The existence of such orthologous clusters with inconsistent prediction results further demonstrated the need for manual curation of predicted chlamydial outer membrane proteins. After detailed analysis of clusters with inconsistent predictions (see Methods section), the remaining 88 clusters could subsequently be used to further categorize those chlamydial proteins with uncertain prediction results. A summary of the predicted outer membrane clusters and their most important features can be found in Table 2.

**Table 2:** Orthologous clusters of predicted chlamydial outer membrane proteins

COMP cluster^1^	C^2^	P^2^	o^2^	Description	No. of proteins	Int^3^	Lip^3^	Exp^4^
001	•	•	•	OppA - Oligopeptide binding proteins, ABC transporters	715		•	

002	•	•	•	Amino acid binding proteins, ABC transporters	453		•	

003	•	•	•	Amino acid binding proteins, ABC transporters	428		•	

004		•	•	TolC - Type I secretion outer membrane proteins	364	•		

005	•		•	PmpB/C - autotransporters/cell surface proteins	352	•		+

006		•	•	Spermidine/putrescine-binding proteins, ABC transporters	324		•	

007	•	•	•	PmpG, PmpH - autotransporters/cell surface proteins	309	•		+

008	•	•	•	Metalloprotease, insulase family proteins; peptidase III proteins	297		•	

009	•	•	•	Omp85 - outer membrane proteins, surface antigen (D15) proteins	294	•		

010	•	•	•	Periplasmic solute binding proteins, ABC transporters	287	•		+

011	•	•	•	Pal - peptidoglycan-associated lipoproteins	279		•	

012		•	•	Outer membrane proteins, multidrug efflux systems	274	•		

013	•	•	•	ApbE - thiamine biosynthesis lipoproteins	265		•	

014		•	•	Efflux transporter proteins, secretion proteins	258	•		

015	•		•	Solute binding proteins, ABC transporters	234		•	

016	•	•	•	TolB - translocation proteins	231		•	

017	•	•	•	PmpG - autotransporters/cell surface proteins	225	•		+

018		•	•	ArcAB (MexAB) OprM - multidrug efflux pump outer membrane proteins	215		•	

019	•	•	•	Mip - peptidyl-prolyl cis-trans isomerases	195	•		

020	•	•	•	SctC - type three secretion system proteins	184	•		+

021		•	•	Omp85 - outer membrane proteins; surface antigen (D15) proteins	180	•		

022		•	•	Outer membrane efflux proteins	163	•		

023	•	•	•	GspD - general secretion pathway proteins	154		•	

024		•	•	Wza - polysaccharide export proteins	143		•	+

025	•		•	PmpG - autotransporters/cell suface proteins	140	•		

026		•	•	Fibronectin/fibrinogen binding proteins	127	•		

027	•		•	Tarp - autotransporters/cell surface proteins	114	•		-

028	•	•	•	SctJ - type three secretion lipoproteins	104		•	-

029	•	•	•	Imp, OstA - organic solvent tolerance proteins	101	•		

030	•	•	•	Skp, OmpH - outer membrane proteins	101	•		

031		•	•	RhsB, RhsD - RHS family proteins	98	•		

032	•		•	PmpB/C - autotransporters/cell surface proteins	94	•		+

033	•		•	DSBA-like thioredoxins, disulfide isomerases	92		•	

034		•	•	Outer membrane efflux proteins	85		•	

035	•		•	PmpD - autotransporters/cell surface proteins	85	•		+

036	•		•	Solute binding proteins, ABC transporters	73		•	

037	•		•	PmpE/F - autotransporters/cell surface proteins	46	•		+

038		•	•	Host cell attachment-required proteins; most proteins hypotheticals	43	•		

039		•	•	Flagellar hook associated proteins; outer membrane/cell surface proteins	43	•		

040	•	•	•	ComL - competence lipoproteins	42		•	

041		•	•	OsmY - osmotically inducible proteins	39		•	

042	•		•	PmpG - autotransporters	38	•		+

043	•		•	Cell surface proteins	27	•	•	~

044	•	•	•	OmcB - 60 kDa cysteine-rich outer membrane protein	27	•		~

045	•		•	PmpA, PmpG - autotransporters/cell surface proteins	27	•		+

046	•		•	PmpE/F - autotransporters/cell surface antigens	25	•		+

047	•		•	Outer membrane proteins, most proteins hypotheticals	23	•		

048	•	•	•	Cell wall associated proteins, most proteins hypotheticals	22	•		

049	•	•	•	MORN motif proteins, kinases, most proteins hypotheticals	21	•	•	

050	•	•	•	Hypothetical proteins	18	•		

051	•		•	OprB - Carbohydrate-selective porins, most proteins hypotheticals	15	•		+

052	•		•	PmpG, PmiI - autotransporters/outer membrane proteins	15	•		+

053		•	•	Outer membrane proteins/invasins, most proteins hypotheticals	15	•		

054	•		•	Flagellar hook proteins, most proteins hypotheticals	12	•		

055	•		•	Hypothetical proteins	12	•		

056	•		•	PmpG - autotransporters/cell surface proteins	12	•		+

057	•		•	Hypothetical proteins	11	•		

058	•	•		Hypothetical proteins	11	•		

059	•	•		Hypothetical proteins	11	•		

060	•	•		Hypothetical proteins	11		•	

061	•	•	•	CHLPN 76 kDa homologues	10	•		~

062	•	•		Hypothetical proteins	10		•	

063	•		•	Hypothetical proteins	10		•	

064	•			OmpA, MOMP - major outer membrane proteins	9	•		+

065	•			Hypothetical proteins	8		•	

066	•			Hypothetical proteins	8		•	

067	•	•	•	Hypothetical proteins	8		•	

068	•		•	OmcA - 9 kDa cysteine-rich outer membrane proteins	8	•		-

069	•			Hypothetical proteins	7	•		

070	•			Hypothetical proteins	7	•		

071	•			Hypothetical proteins	7	•		

072	•			Hypothetical proteins	7	•		

073	•			Hypothetical proteins	7		•	

074		•	•	OmpW - outer membrane proteins	7		•	

075	•			OmpB, PorB - outer membrane proteins	7	•		+

076	•		•	PmpG, hypothetical proteins	7	•		+

077	•		•	Adherence factors, cytotoxins	6	•		

078	•			Hypothetical proteins	6		•	

079	•			Srp - 15 kDa cysteine-rich proteins	6	•		~

080	•			MAC/perforin family proteins	5	•		

081		•		Hypothetical proteins	5	•	•	

082	•			Hypothetical proteins	4		•	

083		•		Hypothetical proteins	4	•		

084		•	•	Hypothetical proteins	3		•	

085		•		Hypothetical proteins	3		•	

086	•			PmpE/F - polymorphic membrane proteins	3	•		+

087		•		Hypothetical proteins	2		•	

088	•			PmpG - polymorphic membrane proteins	2	•		+

^1 ^Cluster number refers to pCOMP http://www.microbial-ecology.net/pcomp.

^2 ^Bullets indicate presence of proteins from *Chlamydiaceae *(C), *Parachlamydiaceae *(P) or other bacteria (other).

^3 ^Cluster contains integral outer membrane proteins (Int) or outer membrane lipoproteins (Lip).

^4 ^Experimental evidence available confirming (+) or contradicting (-) the prediction; ~, ambiguous reports about subcellular location

For categorization of proteins with uncertain prediction results, all proteins classified as cell envelope components but assigned to integral outer membrane clusters were categorized as putative integral outer membrane proteins (see additional file 1: Supporting Information, Figure S1, Table S6). In addition, all proteins not recognized as cell envelope component but found in predicted outer membrane clusters were investigated for possible formation of beta-barrel (supported by at least two predictors) or beta-helix structures. If either of these structures were predicted, these proteins were identified as predicted integral outer membrane proteins; if only one prediction program supported a beta-barrel structure, proteins were predicted as putative integral outer membrane proteins. Similarly, all proteins with an uncertain location in the cell envelope in clusters containing predicted outer membrane lipoproteins were reassigned as putative outer membrane lipoproteins. Proteins in predicted outer membrane clusters that did not match the criteria for reassignment were labelled "ambiguous predictions" (see additional file 1: Supporting Information, Table S6). Altogether, the analysis of orthologues protein clusters could be used to reassign 96 proteins (see additional file 1: Supporting Information, Table S3, Figure S1).

After these analyses, 55 predicted integral outer membrane proteins or outer membrane lipoproteins of *C. trachomatis*, 59 of *C. muridarum*, 66 of *C. caviae*, 70 of *C. pneumoniae *and 62 of *P. amoebophila *could be identified. Our approach was thus able to predict up to 77% more outer membrane proteins than currently recognized (Table 1, Table S3 in additional file 1: Supporting Information). Taking into account that due to the lack of a close relative with a sequenced genome for *P. amoebophila *58 species-specific proteins of uncertain location in the cell envelope were not associated to a cluster and therefore could not be considered in the reassignment step (see additional file 1: Supporting Information, Table S4), the new numbers correspond well with the number of outer membrane proteins expected for the respective genome sizes when compared to other organisms with a similar lifestyle [22].

### pCOMP - a comprehensive database for predicted chlamydial outer membrane proteins

In order to provide a convenient and straightforward interface to the prediction and cluster analysis performed in this study, we have set up the online database pCOMP (predicted Chlamydial Outer Membrane Proteins). The pCOMP database, accessible at http://www.microbial-ecology.net/pcomp provides an overview of all 88 predicted outer membrane protein clusters and the associated proteins from all bacteria included in this study. A unique pCOMP cluster number was assigned to each cluster, which is in the following used as reference. Detailed information on the various prediction results from all applied programs for proteins of the five investigated chlamydial species including their final location prediction is provided. In addition, information about experimentally confirmed chlamydial outer membrane proteins, including links to abstracts at PubMed [36], is available, and protein sequences can be directly accessed at GenBank [36] and UniProt [37]. Several options to search pPCOMP are available, including a free text search applicable for all current protein identifiers, organism and strain names as well as the possibility to BLAST a sequence of interest against all proteins in the database.

### Predicted outer membrane proteins with experimental evidence

Several (n = 26) of the predicted outer membrane protein clusters include proteins whose subcellular location has already been demonstrated experimentally. Selected examples are discussed below.

The first chlamydial proteins described as outer membrane proteins were the members of the chlamydial outer membrane complex (COMC), all of which were predicted as outer membrane proteins in our analysis; the major outer membrane protein OmpA (MOMP) as well as the two cysteine rich proteins OmcA and OmcB (pCOMP clusters 044, 064, and 068). The location of OmpA in the outer membrane and its function as a porin has been shown in numerous publications (see e.g. [38-41]). This is also true for OmcA, which was demonstrated to be located in the outer membrane [42,43] and was furthermore characterised as lipoprotein [44]. Our failure to recognize it as a predicted lipoprotein is due to the settings chosen for LipoP, which resulted in only the best prediction result to be displayed. In OmcA, there is a signal for an SPI site which overrules the SPII site, and the SPII site is therefore not displayed as a result. However, when choosing the output format to display all results, the SPII site is also recognized, but at a lower value than the SPI site. OmcB was sometimes described as a periplasmic protein due to its lack of recognition by TID labelling and the recovery in the soluble protein fraction (and not the membrane fraction), and it was not recognized on the surface of EBs by specific antibodies in several studies [43,45-47]. However, later studies clearly showed its surface exposure and heparin-binding activity. In addition, incubation with purified OmcB blocked host cell infection. OmcB is therefore now considered an important surface-exposed adhesin crucial for host cell infection [48-50].

The largest group of chlamydial outer membrane proteins are the polymorphic membrane proteins (Pmps), which have been identified as autotransporters (pCOMP clusters 005, 007, 017, 025, 032, 035, 037, 042, 045, 046, 052, 056, 076, 086, 088). Autotransporters are proteins which possess a transmembrane domain spanning the outer membrane and mediating its own transport as well as a domain exposed to the extracellular environment, often functioning as adhesins or virulence factors [51]. *Chlamydiaceae *Pmp proteins are the only described autotransporters outside the *Proteobacteria *and are considered essential for host cell interaction. At first only few Pmp proteins could be detected by TID labelling or immunofluorescence of formalin fixed EBs in the chlamydial outer membrane, but the authors of these studies stated that this is likely due to insufficient amounts of proteins to be detected or failure of surface epitope recognition by the antibodies in the respective essay [52,53]. However, the outer membrane location and the function of several Pmps, most dominantly PmpD (Pmp21), has been reported in several studies [54-59], and in a recent study, the expression and surface exposure of all Pmp proteins from *C. trachomatis *has been demonstrated [60]. As further support for their importance in the chlamydial outer membrane, several studies showed disease-correlated serum reactions for Pmp proteins [58,61,62], and there are indications for tissue tropism-related differences in the Pmp proteins based on sequence clustering analyses [17,18].

Additional predicted outer membrane proteins, which are in agreement with experimental evidence, include SctC, a component of the type three secretion apparatus [63], the protein CTL0626 [64] and OmpH [65], as well as PorB, which functions as a porin in the chlamydial outer membrane [66], and the '76 kDa protein' CP0017 [67](pCOMP clusters 020, 030, 051, 061, 075). The Mip protein (pCOMP cluster 019) was originally reported not to be surface exposed [68,69], whereas one study describes it as secreted into the inclusion membrane [70]. It was however also shown to be immunogenic [71,72], and surface exposure was shown by biotinylation of EBs as well as surface immunoprecipitation in a study that suggests the most likely location of Mip to be dual, in the inner as well as the outer membrane [73], which would also explain the contradicting reports in the literature.

Few proteins (n = 4) predicted by our approach are not in agreement with available experimental data. The location of the type three secretion protein SctJ (pCOMP 028) has, to our knowledge, not been demonstrated for *Chlamydiae*; it is however reported to function as a bridge between the inner and outer membrane and thus highly unlikely to be located on the surface of chlamydiae [63]. The same can be concluded for YtgA (pCOMP 010), which has been reported to be associated with an ABC transporter, but is likely to function mostly in the periplasm [74]. The protein Srp (pCOMP 079) was originally reported to be located in the outer membrane and function together with OmcA and OmcB as a third cysteine-rich protein [75-77], but was also shown to be translocated to the inclusion membrane by immunofluorescence [78]. The protein TARP (pCOMP 027) has been shown to be located at the cytoplasmic side of the plasma membrane[79], it is transported through the cell envelope only in the process of being injected into the host cell where it performs actin recruitment to facilitate chlamydial entry (e.g. [80-82]).

### New putative chlamydial outer membrane proteins

Forty two of the 88 obtained orthologous clusters of predicted chlamydial outer membrane proteins (Table 2) contained proteins already described as such or likely to function as such based on their homology to other outer membrane proteins, whereas 46 clusters contained at least one chlamydial protein not yet described as outer membrane protein (annotated as hypothetical protein, and no reports available demonstrating its location). Altogether, 143 new *Chlamydiaceae *and 94 new *P. amoebophila *outer membrane candidates were thus identified in this study (Table 2; additional file 1: Supporting Information, Table S3, S4). In previous studies, 112 of those were shown to be transcribed [83-85] and 58 were confirmed as expressed proteins [48,86-91]. This extended set of putative chlamydial outer membrane proteins should represent important targets for further experimental characterization. As an example, eight orthologous clusters containing hypothetical proteins are shared by both *Chlamydiaceae *and *Parachlamydiaceae *(see pCOMP clusters 048-050, 058-060, 062, 067), and four of these clusters include proteins from all chlamydial organisms (pCOMP 049, 058-060). Those proteins could represent cell envelope features which remained hidden so far but are shared by all chlamydiae.

**Figure 3:**
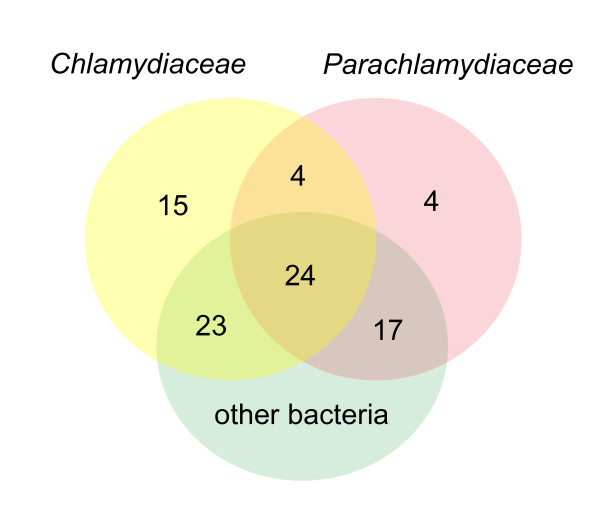
**Taxonomic distribution of the 88 chlamydial outer membrane clusters**. The Venn diagram shows the numbers of clusters that contain at least one protein from the *Chlamydiaceae*, the *Parachlamydiaceae *or other bacteria, and the respective intersections.

Twenty three orthologous clusters contain proteins from the *Parachlamydiaceae *and/or the *Chlamydiaceae *but no orthologues from other bacteria and thus represent chlamydia-specific outer membrane proteins (Figure 3, Table 2). It is remarkable though, that only four of these clusters comprised proteins from both chlamydial families and that not a single cluster included proteins from all chlamydial species. This demonstrates that the outer membrane of the *Chlamydiae *has undergone drastic changes during evolution after the emergence of extant *Parachlamydiaceae *and *Chlamydiaceae*. The observed expansion of outer membrane proteins in the *Chlamydiaceae *(15 clusters) compared to the *Parachlamydiaceae *(4 clusters) might reflect the highly adapted lifestyle of the *Chlamydiaceae *as parasites of vertebrates.

### A putative autotransporter in *P. amoebophila*

Interestingly, no evidence for Pmps was found previously in the genome of the amoeba symbiont *P. amoebophila *[23]. In our analysis, indeed all except one Pmp cluster contain *Chlamydiaceae *but no *Parachlamydiaceae *proteins (Table 2). The only exception is a cluster (pCOMP cluster 017) which comprises putative autotransporter and cell surface proteins from *C. pneumoniae *and other bacteria, as well as one hypothetical protein from *P. amoebophila*. It will be interesting to decipher the role of this *P. amoebophila *protein, which might function as an adhesin to attach to amoeba cell membranes. However, the apparent overrepresentation of autotransporters in the *Chlamydiaceae *compared to *P. amoebophila *might indicate a strong effect of life style and ecological niche on the composition of the outer membrane proteins. In fact, Pmps constitute the most diverse family of outer membrane proteins in the *Chlamydiaceae*, suggesting an essential role of these proteins as virulence factors for the infection of vertebrate (as opposed to protozoan) host cells.

### A set of enigmatic outer membrane lipoproteins specific for chlamydiae

Thirty one predicted outer membrane protein clusters obtained in our analysis contain only lipoproteins (Table 2). Lipoproteins are a molecular consortium of protein and lipid, which anchors them in the bacterial cytoplasmic or outer membrane. Most characterized bacterial lipoproteins assist periplasmic transport of components imported by ABC transporters. However, some outer membrane lipoproteins are also known to be exposed to the outside of bacterial cells (e.g. [92]). The predicted lipoprotein clusters of chlamydiae can be divided into two groups; on the one hand they contain well-described proteins, mostly components of ABC transport systems, which are likely anchored in the outer membrane and exposed to the periplasm and perform a general function conserved throughout a great diversity of bacteria. On the other hand, about half of the lipoprotein clusters contain exclusively chlamydial proteins all of which are annotated as hypothetical proteins. Therefore, lipoproteins seem to be a class of proteins in which the chlamydial diversification from all other bacteria is particularly pronounced. Due to the absence of experimental data for most of the chlamydial lipoproteins, their function remains enigmatic.

### Species-specific outer membrane proteins might reflect the host spectrum

One protein from *C. trachomatis*, four proteins from *C. muridarum*, 10 proteins from *C. caviae*, 17 proteins from *C. pneumoniae*, and 72 proteins from *P. amoebophila *were predicted as outer membrane proteins but not assigned to a cluster within the threshold applied in this study (additional file 1: Supporting Information, Table S4). These proteins are therefore considered species-specific proteins (pCOMP group 089). Independent of their origin, these proteins must have undergone rapid evolution obscuring a detectable sequence homology. For most of them no unambiguous prediction was inferred and they were thus classified as predicted cell envelope components. However, for *C. pneumoniae*, five predicted integral outer membrane proteins could be identified (CP1072, CP1074, CP1075, CP1076, and CP1077). Their arrangement in the same region of the genome might indicate that they originated from multiple gene duplication events, and it is tempting to speculate that these *C. pneumoniae *specific outer membrane proteins contribute to tissue and host specificity of these microorganisms. *C. pneumoniae *has been shown to thrive in a wide variety of hosts (14 species; [6]) and encodes a significantly wider spectrum of outer membrane proteins than the two species *C. trachomatis *and *C. muridarum*, which show the smallest and therefore most specified pool of predicted outer membrane proteins, and which are found in only one or two host species (humans; mouse and hamster, respectively; [2,93,94]).

The greatest number of species-specific proteins was found for *P. amoebophila *(additional file 1: Supporting Information, Table S4). Within these 72 proteins, there are 11 predicted integral outer membrane proteins (pc0036, pc0074, pc0790, pc1030, pc1071, pc1295, pc1304, pc1463, pc1862, pc1863 and pc1864) and three predicted outer membrane lipoproteins (pc0291, pc0498, and pc0606) without any detectable orthologues. The high number of apparent species-specific proteins in *P. amoebophila *shows that these chlamydial symbionts are remarkably different from the *Chlamydiaceae *with respect to their repertoire of predicted outer membrane proteins, which most likely represents an adaptation to the environmental life style and a variety of amoeba and possibly also other protozoan hosts.

### Outer membrane proteins show a rapid evolutionary rate

Using cluster analysis, taxonomic profiles of predicted outer membrane proteins of *Chlamydiaceae *and *Parachlamydiaceae *clustered together but showed a lower similarity (91%) than those of proteins not predicted to be located in the outer membrane (97%; additional file 1: Supporting Information, Table S7, S8, Figure S2). This suggests that outer membrane proteins are among the fastest evolving groups of chlamydial proteins and thus contributed most to differentiation of life style and host spectrum of chlamydiae. A similar role is in fact discussed for members of the Pmp family, the largest family of *Chlamydiaceae *outer membrane proteins, which are implicated in tissue specificity of *C. trachomatis *disease groups [17,18,95]. These adaptations might be similar to the Sca proteins of *Rickettsia *species [96] or the mosaic genes of *Anaplasma *species [97], two other bacterial groups containing important obligate intracellular human pathogens.

### Chlamydiae share predicted outer membrane proteins with other intracellular pathogens

A comparison of the taxonomic profiles of chlamydial proteins with respect to the categories free-living, facultative or obligate intracellular (additional file 1: Supporting Information, Table S7) with those of the free-living model organism *E. coli *showed no obvious differences between *E. coli*, the *Chlamydiaceae *and the *Parachlamydiaceae*, respectively (additional file 1: Supporting Information, Figure S3, Table S9). When only the presence or absence of orthologues from obligate intracellular human pathogens was analysed, again, no differences between the two chlamydial families were seen, demonstrating that the resolution of this approach with respect to host adaptation is limited (additional file 1: Supporting Information, Figure S4, Table S10). However, both *Chlamydiaceae *and *Parachlamydiaceae *showed marked differences compared to the free-living model organism *E. coli*. The higher number of clusters with orthologues from obligate intracellular human pathogens in the *Chlamydiae *suggests that indeed the lifestyle of an organism has a profound effect on its outer membrane proteins and that - in addition to host and tissue specific adaptations - general features are shared between phylogenetically largely unrelated groups of bacteria with the same life style.

### Signatures of the chlamydial evolutionary history

Interestingly, the vast majority of outer membrane proteins of both *Chlamydiaceae *and *Parachlamydiaceae *have orthologues in at least one of the three phyla *Planctomycetes*, *Verrucomicrobia*, or *Lentisphaerae *(additional file 1: Supporting Information, Figure S5, Table S11). The overrepresentation of orthologues with these phyla, compared to *E. coli*, supports an ancient relationship of *Chlamydiae *with *Verrucomicrobia, Lentisphaerae *and *Planctomycetes*, which were recently proposed to constitute the so-called PVC superphylum [98,99], and suggests that a number of outer membrane proteins were retained from their last common ancestor.

## Conclusions

*In silico *prediction of chlamydial outer membrane proteins and subsequent analysis of orthologous clusters resulted in a comprehensive collection of chlamydial outer membrane proteins, revealing major differences among chlamydial organisms with respect to their cell envelope. In addition, phylogenetic profiling of predicted chlamydial outer membrane proteins uncovered similarities of the chlamydial outer membrane to those of other human pathogens and allowed insights into ancient evolutionary relationships of the *Chlamydiae*. As our prediction approach included several manual curating steps and followed a highly conservative policy minimizing false positives, this collection represents a reliable resource of predicted chlamydial outer membrane proteins.

Chlamydiae cause some of the most widespread diseases of humans worldwide [1-3], and the need for an anti-chlamydial vaccine was thus already recognized decades ago [100]. Interestingly, recently identified putative chlamydial B and T cell antigens [101,102] matched proteins from five pCOMP clusters, one of them not recognized previously as outer membrane protein. The first extensive overview of chlamydial outer membrane proteins provided in this study might thus also provide a solid basis for and help in the quest for an anti-chlamydial vaccine.

## Methods

### Software used for prediction of protein structure and location

Signal peptide predictions were performed at the the SignalP 3.0 server http://www.cbs.dtu.dk/services/SignalP/ using the model for Gram-negative bacteria and the combination of Neural Networks and Hidden Markov Models [103] and the TargetP 1.1 server http://www.cbs.dtu.dk/services/TargetP/ using the settings for Non-plant organisms and the 'no cutoffs, winner-takes-all' setting without cleavage site prediction [60]. Alpha-helical transmembrane regions were investigated using the TMHMM 2.0 server http://www.cbs.dtu.dk/services/TMHMM/[104]. Further information about putative protein location was obtained from the cPsortdb database http://db.psort.org[34]. Four different servers were used for the identification of putative beta-barrel structures; the Beta-barrel Outer Membrane protein Predictor (BOMP) http://www.bioinfo.no/tools/bomp[105], the Prediction of TransMembrane Beta-Barrel Proteins server (PRED-TMBB) http://bioinformatics.biol.uoa.gr/PRED-TMBB/[30], the Markov Chain Model for Beta Barrels prediction program (MCMBB) http://athina.biol.uoa.gr/bioinformatics/mcmbb/[106] and the B2TMR-HMM predictor http://gpcr.biocomp.unibo.it/predictors/[31]. For PRED-TMBB, the predictions were performed using the Viterbi and Posterior Decoding algorithms. The probability of the proteins to form a beta-helix was investigated with BetaWrap http://groups.csail.mit.edu/cb/betawrap/[65]. Lipoproteins were predicted using the LipoP 1.0 server with the 'short' output format selected http://www.cbs.dtu.dk/services/LipoP/[107]. Additional information about individual proteins was obtained from the PEDANT database http://pedant.gsf.de[108].

### *In silico *approach for prediction of outer membrane proteins

Starting with the *in silico *proteome of an organism, the first step was to select proteins according to general characteristics of outer membrane proteins also shared with periplasmic proteins (Figure 2). This should mainly exclude cytoplasmic or cytoplasmic membrane proteins. In detail, to exclude cytoplasmic membrane proteins, all proteins with more than one transmembrane helix predicted by TMHMM [104] were considered cytoplasmic membrane proteins and thus removed. Proteins with only one predicted transmembrane helix were not removed in this step as signal peptides are sometimes falsely recognized as transmembrane helix. From the remaining proteins, those labelled to be located in the cytoplasm, the cytoplasmic membrane, or the periplasm in cPsortdb [34], a database providing access to location predictions obtained by a variety of methods, were removed leaving only proteins with "unknown" or "outer membrane" location. All proteins designated "outer membrane" were automatically chosen for the first set of outer membrane protein candidates. All proteins with "unknown" location in cPsortdb were subsequently analyzed for the presence of a signal peptide using SignalP [103] and TargetP [109]. If both or only one of the two servers predicted the protein to be secreted, it was classified as containing a signal peptide. The presence of a signal peptide indicates that a protein is secreted (in a sec-dependent manner) and therefore potentially located in the cell envelope. Proteins without a predicted signal peptide were thus removed, resulting in the first set of outer membrane protein candidates, which should still include false positive proteins from the cell envelope, e.g. periplasmic proteins not recognized as such by the conservative approach of cPsortdb (Figure 2).

In the second step, the obtained initial set of putative outer membrane proteins was curated manually by taking into account the annotation of each protein as well as additional information provided by the PEDANT database [110], including hits to conserved protein domains and patterns derived from Interpro [111], PIR [112], SCOP [113], and hits to COG [114]. To remove false positives, proteins annotated as or showing evidence for cytoplasmic, cytoplasmic membrane or periplasmic proteins were omitted from the list of outer membrane protein candidates (Figure 2). This rather conservative approach could lead to generating false negatives due to an incorrect annotation. However, we regarded the sequence homology based annotation of proteins as strong evidence for functional conservation. In particular for the evolutionary well separated chlamydiae, it seems highly likely that a chlamydial protein still showing sequence similarity to a protein from other bacteria is functionally conserved and thus located in the same cellular compartment (with the exception of fusion proteins). In addition, in order to not miss any potential outer membrane proteins among those without any indication for their location, i.e. proteins labelled by cPsortdb as "unknown" and lacking a recognized signal peptide, their annotation was considered. Proteins annotated to function in the outer membrane were added to the list of outer membrane proteins, resulting in the second set of outer membrane candidates (Figure 2).

The third and final step aimed at the prediction of integral outer membrane proteins and outer membrane lipoproteins within the reduced set of outer membrane candidates. Integral outer membrane proteins generally form so called beta-barrels consisting of anti-parallel beta-sheets within the lipid bilayer [115]. Since recognizing this structure is still a challenge for prediction algorithms, four different beta-barrel predictors were used (BOMP, [105]; PRED-TMBB, [30]; MCMBB, [106]; and B2TMR-HMM, [31]), and already two out of four predictors identifying the proteins as beta-barrels were counted as positive. An additional structure often found in adhesins and toxins is the beta-helix. All outer membrane protein candidates were therefore subjected to the program BetaWrap [116] and results obtaining a p-value < 0.01 were counted as positive. A predicted beta-helix marked the protein as likely functioning as an adhesin located in the outer membrane. The final set of predicted integral outer membrane proteins thus contained proteins identified either as beta-barrel or beta-helix proteins. Furthermore, the program LipoP [107] was used to identify potential outer membrane lipoproteins as described by Seydel and coworkers [28]. A positive result from LipoP overruled any eventual beta-barrel predictions, because LipoP predictions were previously shown to be highly specific [107,117]. Proteins labelled by cPsortdb as "unknown", lacking a signal peptide and a conclusive annotation were also analyzed with LipoP and, if recognized, were included in the final set of predicted outer membrane lipoproteins. All proteins not predicted as beta-barrel, beta-helix or as outer membrane lipoproteins were assigned to a group designated cell envelope components, which are considered to be likely associated with the cell envelope, but for which no unambiguous prediction was obtained (Figure 2).

### Identification of clusters of orthologous outer membrane proteins

The Similarity Matrix of Proteins (SIMAP) database [118] provides a precalculated sequence similarity matrix for all proteins deposited at major public sequence databases. For the formation of orthologous clusters, bidirectional best hits (BBHs) with an E-value cut-off of 1^-08 ^and a length ratio cut-off of 0.5 were grouped. All chlamydiae (including the yet unfinished genomes of *Parachlamydia acanthamoebae *UV7, *Simkania negevensis *Z, and *Waddlia chondrophila *2032/99; ingroup 1) or a selection of *Proteobacteria *including *E. coli *K12 (ingroup 2) were considered as "ingroup" organisms in our analysis, respectively, whereas 438 and 427 representatives of other bacterial lineages were considered "outgroup" organisms, respectively; for a detailed list of ingroup and outgroup organisms see additional file 1: Supporting Information, Table S7. First, BBHs between proteins from ingroup organisms were merged to form one cluster if they shared at least one protein. Subsequently, outgroup proteins with BBHs to ingroup proteins were added to the clusters. As a last step, in-paralogues (i.e. paralogues that arose after diversification; [119] were added if they showed a higher similarity to a protein from the same organism than to proteins from other ingroup organisms.

When clustering was applied using the *Chlamydiae *as ingroup, 1,911 clusters were obtained in total, from which 190 contained at least one protein predicted as outer membrane protein. 81 of these clusters included two or more proteins from the five analysed chlamydiae, but not all of these proteins were predicted to be located in the outer membrane. These clusters were termed inconsistent clusters and analysed in more detail. 50 out of the 81 inconsistent clusters were not consistent with respect to results of signal peptide prediction, annotation, or both. Inconsistent clusters could either result from the failure of signal peptide prediction methods to reliably recognize secreted proteins of chlamydiae, or from loosely assembled clusters comprising non-orthologous proteins with different function and location.

To check whether the inconsistent clusters resulted from a low cut-off value used for cluster formation, the proteins from each of the inconsistent clusters were aligned by ClustalW and subjected to phylogenetic analysis. For this, a ClustalW alignment was generated with the program MEGA 4.0 using the default settings [120,121] and neighbour-joining and parsimony trees were calculated using default options. For neighbour-joining, either the Poisson or the p-distance correction was used. 1,000 bootstrap replicates were calculated for both treeing methods. In 64 out of the 81 inconsistent clusters all chlamydial proteins grouped together. We therefore assumed that in these clusters, function and location of these proteins are conserved and they were hence used for further analyses (Figure 1). In contrast, a non-monophyletic grouping could be caused either by proteins of different function/location or could be a consequence of lateral gene transfer. As this is difficult to resolve, we omitted the 17 clusters in which the chlamydial proteins did not cluster together from further analysis. From the resulting 173 clusters, those containing exclusively chlamydial proteins with uncertain predictions (i.e. classified as cell envelope components) were not further considered if no orthologues from other bacteria were clearly annotated as outer membrane proteins, as these most likely represent proteins from the periplasm or peptidoglycan-binding proteins. In addition, clusters containing chlamydial proteins predicted as outer membrane protein but also bacterial orthologues experimentally verified as cytoplasmic, cytoplasmic membrane or periplasmic proteins were omitted.

### Taxonomic profiles

Taxonomic profiles of chlamydial proteins based on the obtained clusters were analysed using a Bray-Curtis similarity matrix [122] and subsequent cluster analysis with complete linkage using the program PRIMER 5.0. The phylum *Proteobacteria *is by far the largest and most diverse phylum and was therefore treated separately as *Alpha*-, *Beta*-, *Gamma*-, *Delta*-, *Epsilon*- and unclassified *Proteobacteria*, respectively, for this analysis.

## Authors' contributions

EH and MH designed the study. EH performed the protein predictions, the manual curation steps and the regrouping of predicted proteins and clusters, and the analyses of the taxonomic profiles. PT and TR performed the cluster calculations, which were further analysed and interpreted by EH and MH. EH generated the underlying database, EH and MH designed, and MH set up the online database COMP. EH drafted the manuscript; all authors edited, read and approved the final manuscript.

## Supplementary Material

Additional file 1 **Supporting information**. PDF file containing Figures S1-S5, and Tables S1-S11. Click here for file
