# Supplementary material for: Comprehensive in silico prediction and analysis of chlamydial outer membrane proteins reflects evolution and life style of the Chlamydiae
Source: BMC Genomics. 2009 Dec 29;10:634. doi: 10.1186/1471-2164-10-634 (PMC2811131; doi:10.1186/1471-2164-10-634)
Supplement: Additional file 1 — Supporting information. PDF file containing Figures S1-S5, and Tables S1-S11. [file 1471-2164-10-634-S1.PDF]

Supporting information

Comprehensive *in silico* prediction and analysis of chlamydial outer membrane proteins reflects evolution and life style of the *Chlamydiae*

Heinz et al.

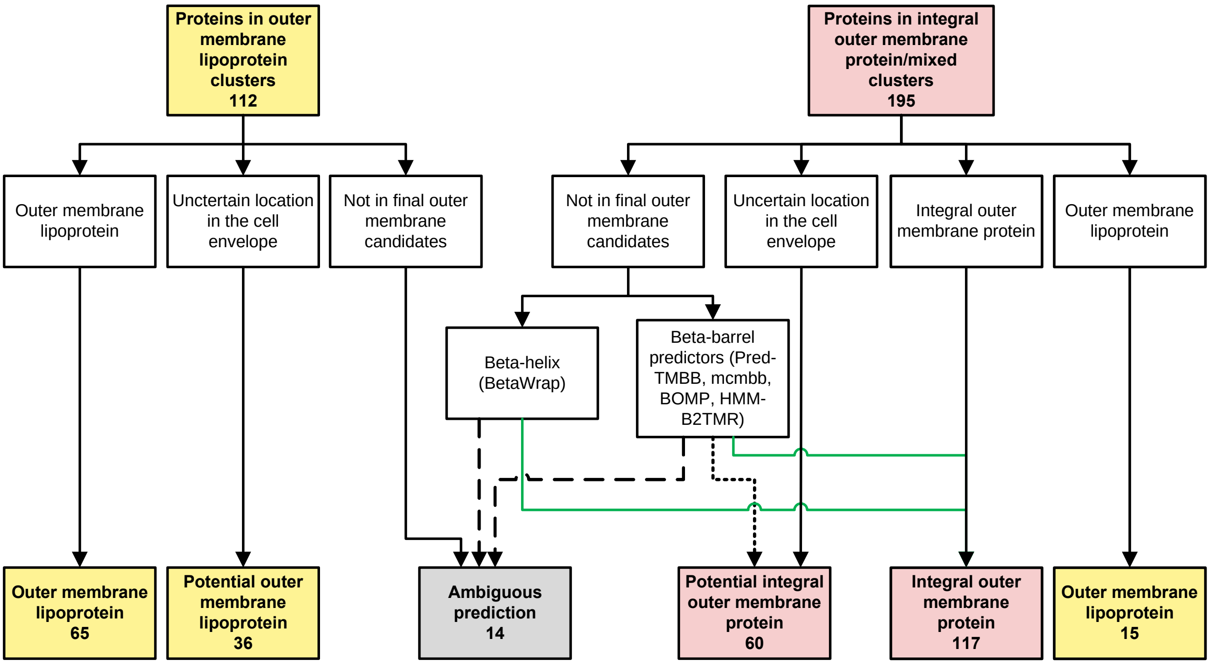

Figure S1: Reclassification of the outer membrane proteins based on orthologous clusters. Proteins with uncertain location were reclassified if they formed clusters with proteins with unambiguous location. Prediction programs were used in uncertain cases. Green arrows indicate a positive prediction result, broken arrows indicate a negative result of the respective prediction programs. Dotted line indicates that respective proteins were predicted to form a beta barrel by one out of four prediction programs. Numbers represent total respective protein numbers for all five chlamydial organisms investigated.

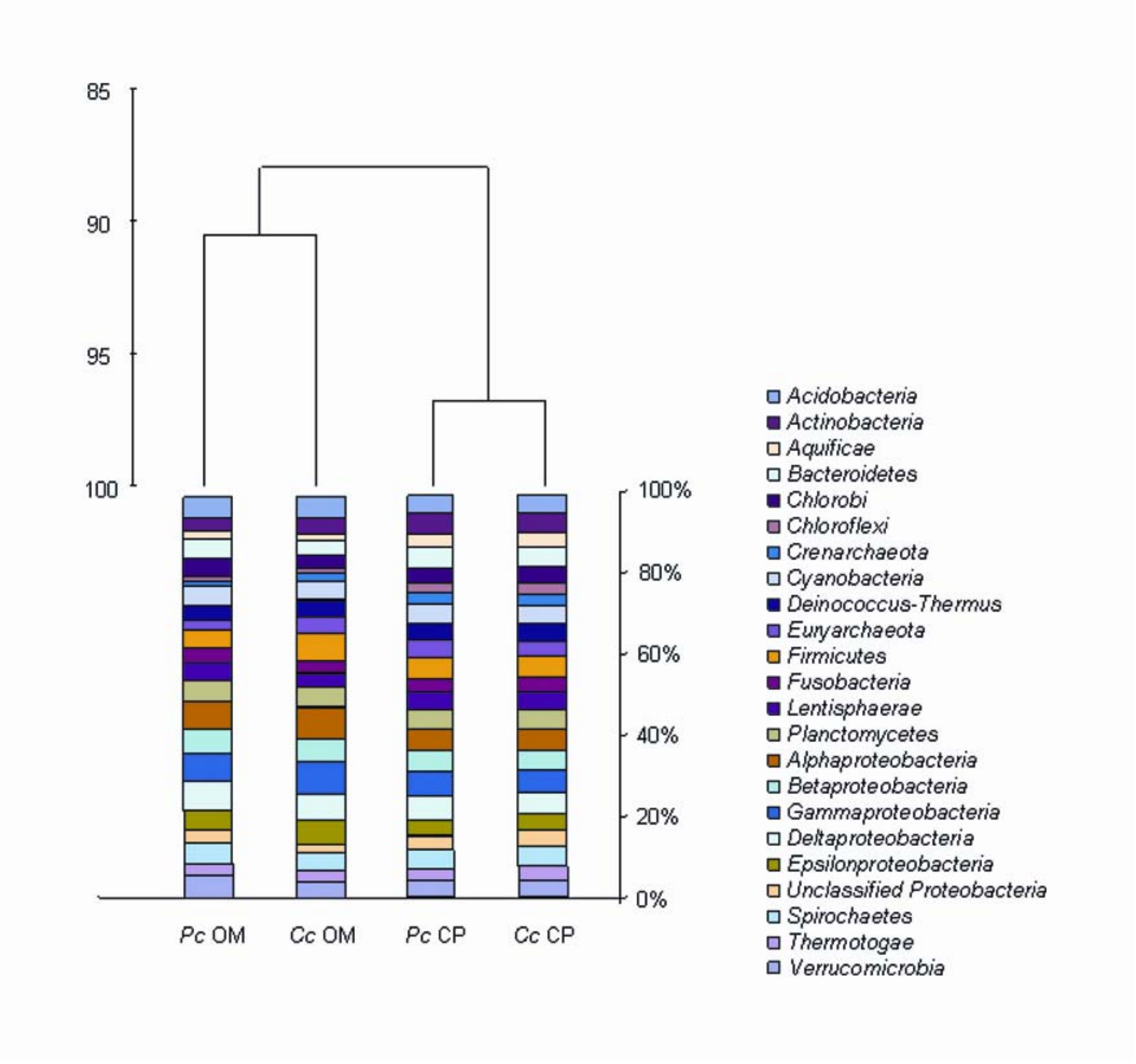

Figure S2: Taxonomic profiles of chlamydial proteins. Outer membrane protein clusters (OM) are compared with clusters containing proteins not located in the outer membrane (cytoplasm, periplasm, cytoplasmic membrane, extracellular; CP) for the *Chlamydiaceae* (Cc) and the *Parachlamydiaceae* (Pc). See Table S7 for details.

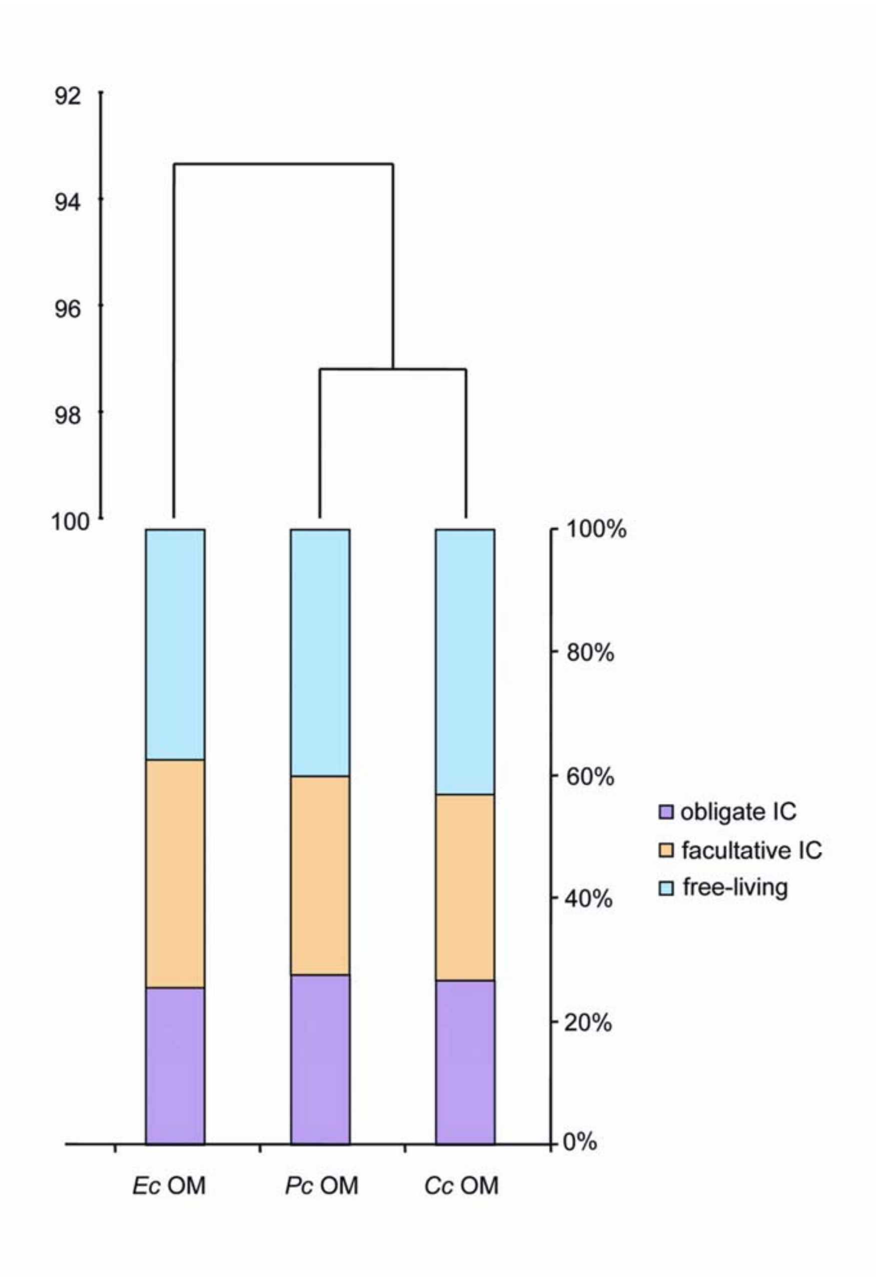

Figure S3: Classification of orthologous clusters based on life style. The distribution of orthologues from organisms with either an obligate intracellular (obligate IC), facultative intracellular (facultative IC) or free-living lifestyle is shown for clusters containing predicted outer membrane proteins from *Parachlamydiaceae* (*Pc* OM), from *Chlamydiaceae* (*Cc* OM) and from *E. coli* (*Ec* OM). The list of organisms and their lifestyles is available as Table S6. Details on the clusters are provided in Table S8.

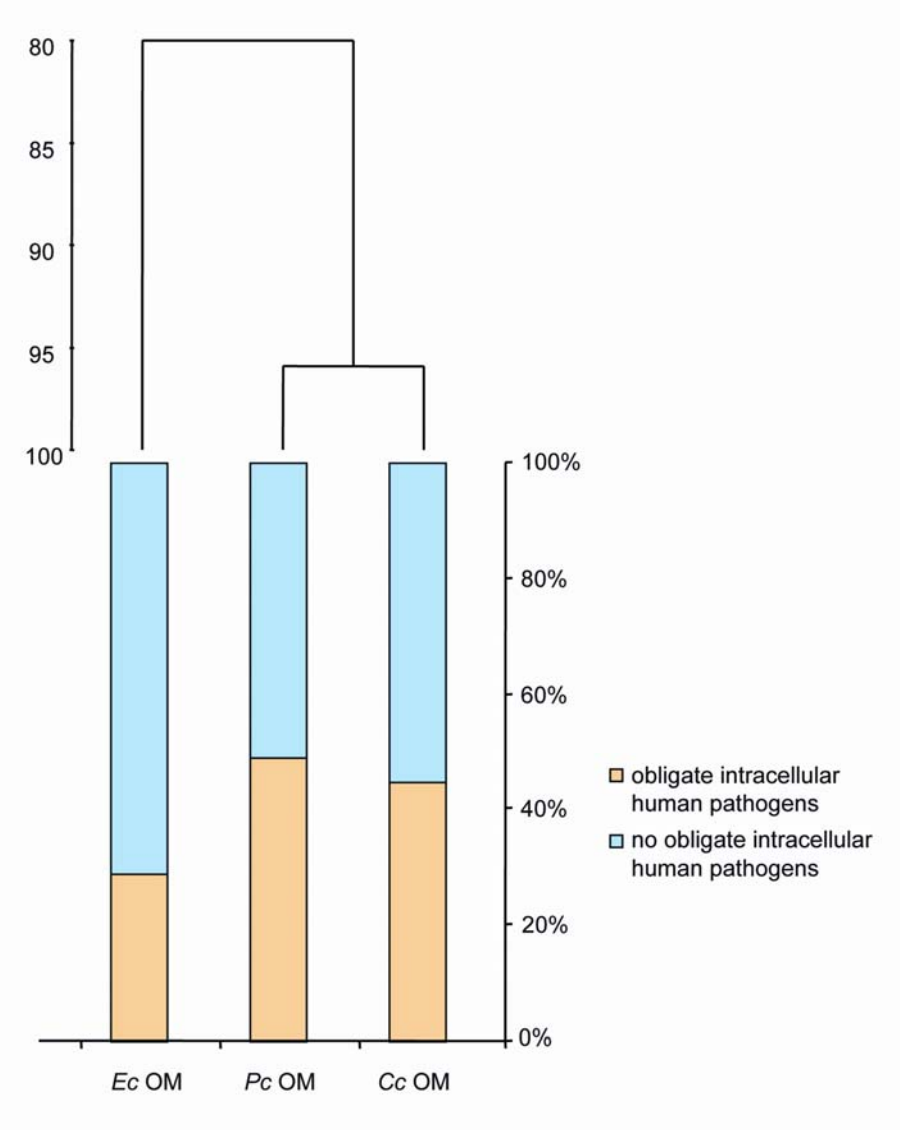

Figure S4: Classification of orthologous clusters based on pathogenicity. The distribution of orthologues from known obligate intracellular pathogens of humans (Table S6) is shown for clusters containing predicted outer membrane proteins from *Parachlamydiaceae* (*Pc* OM), from *Chlamydiaceae* (*Cc* OM) and from *E. coli* (*Ec* OM). Details on the clusters are provided in Table S9.

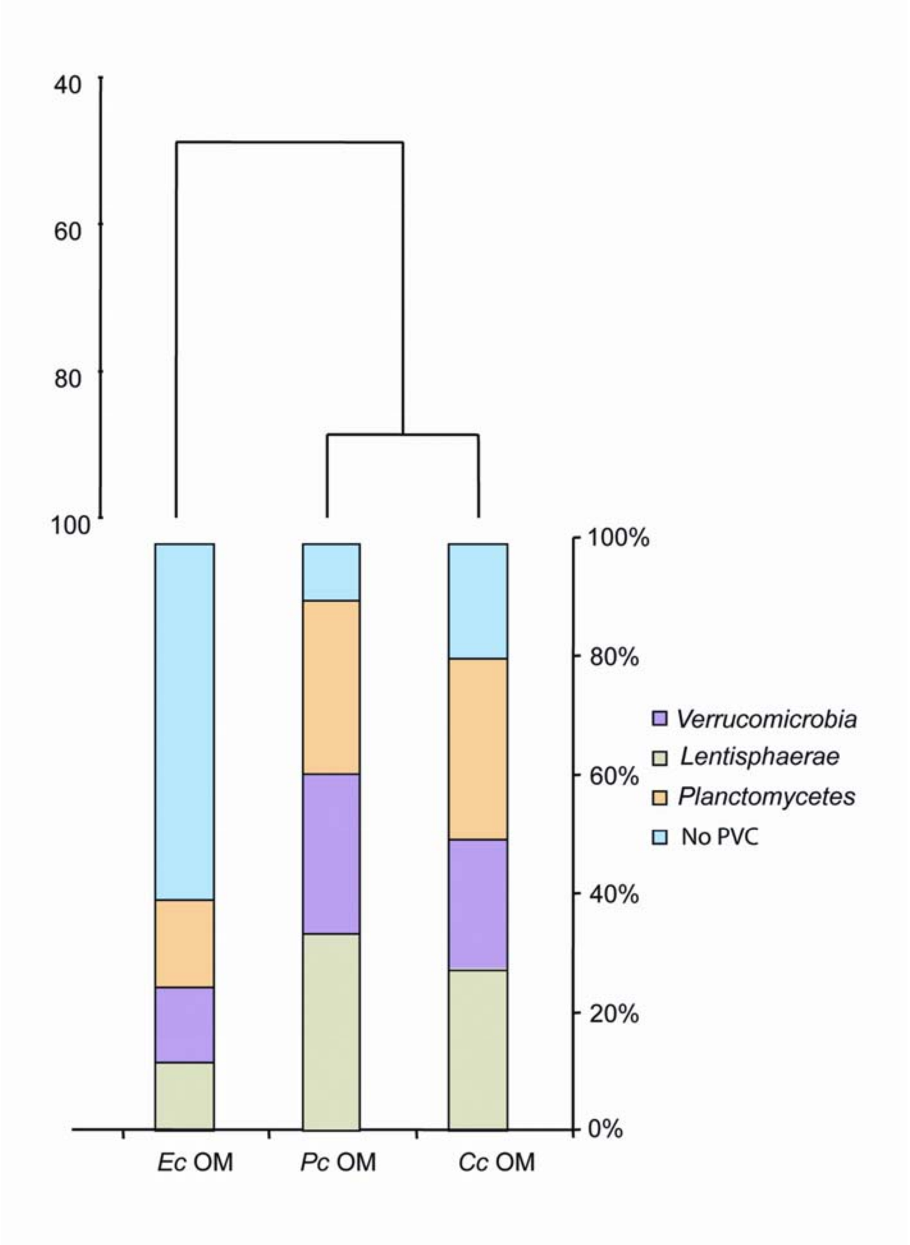

Figure S5: Representation of the PVC superphylum in the outer membrane clusters. The occurrence of *Verrucomicrobia*, *Lentisphaerae* and *Planctomycetes*, all members of the PVC superphylum, is shown for clusters containing outer membrane proteins from *Parachlamydiaceae* (*Pc* OM), from *Chlamydiaceae* (*Cc* OM) and from *E. coli* (*Ec* OM). Details on the clusters are provided in Table S10.

Table S1: Predicted outer membrane proteins from *E. coli* K12.

Gene	Gi	Annotation	TargetP	SignalP	TMHMM	Cpsortdb	Betawrap	MCMBB	BOMP	Pred-TMBB	HMBMM-B2R	LipoP	Experimental verification
**integral outer membrane proteins**													
flgD	16129038	flagellar hook assembly protein	Non-secretory protein	Signal peptide	No alpha-helix	unknown	p>0,01	0,053	-	2,851	No beta-barrel	No lipoprotein	EchoLocation
csgB	16129004	curlin nucleator protein, minor subunit	Signal peptide	Signal peptide	No alpha-helix	unknown	p>0,01	0,052	-	2,901	No beta-barrel	No lipoprotein	EchoLocation
btuB	16131804	vitamin B12/cobalamin outer membrane transporter	Signal peptide	Signal peptide	No alpha-helix	outer membrane	0,00071	0,059	3	2,91	Beta-barrel	No lipoprotein	EchoLocation
bglH	16131588	carbohydrate-specific outer membrane porin, cryptic	Signal peptide	Signal peptide	No alpha-helix	outer membrane	0,0026	0,02	4	2,957	Beta-barrel	No lipoprotein	EchoLocation
flu	49176177	CP4-44 prophage; antigen 43 (Ag43) phase-variable biofilm formation autotransporter	Non-secretory protein	Signal peptide	1 alpha-helix	outer membrane	0,0029	0,054	-	2,891	Beta-barrel	No lipoprotein	EchoLocation
fhuA	16128143	ferrichrome outer membrane transporter	Non-secretory protein	Signal peptide	No alpha-helix	outer membrane	0,0043	0,032	4	2,926	Beta-barrel	No lipoprotein	EchoLocation
yaeT	16128170	conserved protein	Signal peptide	Signal peptide	No alpha-helix	outer membrane	0,0074	0,032	5	2,954	Beta-barrel	No lipoprotein	EchoLocation
uidC	90111306	predicted outer membrane porin protein	Signal peptide	Signal peptide	1 alpha-helix	outer membrane	p>0,01	0,032	1	2,894	Beta-barrel	No lipoprotein	EchoLocation
ompA	16128924	outer membrane protein A	Signal peptide	Signal peptide	No alpha-helix	outer membrane	p>0,01	0,037	1	2,894	Beta-barrel	No lipoprotein	EchoLocation
fimH	16132141	minor component of type 1 fimbriae	Signal peptide	Signal peptide	No alpha-helix	unknown	p>0,01	0,03	1	2,913	Beta-barrel	No lipoprotein	EchoLocation
fhuE	16129065	ferric-rhodotorulic acid outer membrane transporter	Signal peptide	Signal peptide	No alpha-helix	outer membrane	p>0,01	0,046	1	2,932	Beta-barrel	No lipoprotein	EchoLocation
fecA	16132112	KpLE2 phage-like element; ferric citrate outer membrane transporter	Signal peptide	Signal peptide	No alpha-helix	outer membrane	p>0,01	0,043	1	2,934	Beta-barrel	No lipoprotein	EchoLocation
fiu	16128773	predicted iron outer membrane transporter	Signal peptide	Signal peptide	1 alpha-helix	outer membrane	p>0,01	0,05	2	2,943	Beta-barrel	No lipoprotein	EchoLocation
tsx	16128396	nucleoside channel, receptor of phage T6 and colicin K	Signal peptide	Signal peptide	No alpha-helix	outer membrane	p>0,01	0,056	3	2,944	Beta-barrel	No lipoprotein	EchoLocation
fimD	16132138	outer membrane usher protein, type 1 fimbrial synthesis	Non-secretory protein	Signal peptide	1 alpha-helix	outer membrane	p>0,01	0,046	3	2,949	Beta-barrel	No lipoprotein	EchoLocation
ompW	16129217	outer membrane protein W	Signal peptide	Signal peptide	No alpha-helix	outer membrane	p>0,01	0,036	4	2,9	Beta-barrel	No lipoprotein	EchoLocation
fepA	16128567	iron-enterobactin outer membrane transporter	Signal peptide	Signal peptide	No alpha-helix	outer membrane	p>0,01	0,04	4	2,921	Beta-barrel	No lipoprotein	EchoLocation
cirA	16130093	ferric ion-catecholate outer membrane transporter	Signal peptide	Signal peptide	No alpha-helix	outer membrane	p>0,01	0,015	4	2,922	Beta-barrel	No lipoprotein	EchoLocation
pldA	16131671	outer membrane phospholipase A	Signal peptide	Signal peptide	No alpha-helix	outer membrane	p>0,01	0,03	4	2,931	Beta-barrel	No lipoprotein	EchoLocation
ompX	16128782	outer membrane protein	Signal peptide	Signal peptide	1 alpha-helix	outer membrane	p>0,01	0,062	5	2,822	Beta-barrel	No lipoprotein	EchoLocation
ompN	16129338	outer membrane pore protein N, non-specific	Signal peptide	Signal peptide	No alpha-helix	outer membrane	p>0,01	0,061	5	2,841	Beta-barrel	No lipoprotein	EchoLocation
ompC	16130152	outer membrane porin protein C	Signal peptide	Signal peptide	No alpha-helix	outer membrane	p>0,01	0,066	5	2,844	Beta-barrel	No lipoprotein	EchoLocation
ompF	16128896	outer membrane porin 1° (Ia;b;F)	Signal peptide	Signal peptide	No alpha-helix	outer membrane	p>0,01	0,067	5	2,847	Beta-barrel	No lipoprotein	EchoLocation
phoE	16128227	outer membrane phosphoporin protein	Signal peptide	Signal peptide	No alpha-helix	outer membrane	p>0,01	0,05	5	2,849	Beta-barrel	No lipoprotein	EchoLocation
mipA	16129736	scaffolding protein for murein synthesizing machinery	Signal peptide	Signal peptide	No alpha-helix	outer membrane	p>0,01	0,044	5	2,862	Beta-barrel	No lipoprotein	EchoLocation
ompG	16129280	outer membrane porin	Signal peptide	Signal peptide	No alpha-helix	outer membrane	p>0,01	0,026	5	2,871	Beta-barrel	No lipoprotein	EchoLocation
fadL	145698292	long-chain fatty acid outer membrane transporter	Signal peptide	Signal peptide	No alpha-helix	outer membrane	p>0,01	0,051	5	2,881	Beta-barrel	No lipoprotein	EchoLocation

ompT	16128548	DLP12prophage; outer membrane protease VII (outer membrane protein 3b)	Signal peptide	Signal peptide	No alpha-helix	outer membrane	p>0,01	0,047	5	2,903	Beta-barrel	No lipoprotein	EchoLocation
ompL	16131715	predicted outer membrane porin L	Signal peptide	Signal peptide	No alpha-helix	outer membrane	p>0,01	0,003	5	2,938	Beta-barrel	No lipoprotein	EchoLocation
lamB	16131862	maltose outer membrane porin (maltoporin)	Signal peptide	Signal peptide	No alpha-helix	outer membrane	p>0,01	0,027	4	>2,965	Beta-barrel	No lipoprotein	EchoLocation
imp	16128048	exported protein required for envelope	Signal peptide	Signal peptide	No alpha-helix	outer membrane	p>0,01	0,022	1	>2,965	Beta-barrel	No lipoprotein	EchoLocation
pagP	16128605	palmitoyl transferase for Lipid A	Signal peptide	Signal peptide	No alpha-helix	outer membrane	p>0,01	0,014	5	>2,965	Beta-barrel	No lipoprotein	EchoLocation
tolC	90111528	transport channel	Signal peptide	Signal peptide	No alpha-helix	outer membrane	p>0,01	0,063	-	2,9	Beta-barrel	No lipoprotein	EchoLocation
gspD	90111569	general secretory pathway component, cryptic	Signal peptide	Signal peptide	No alpha-helix	outer membrane	p>0,01	0,012	-	2,924	No beta-barrel	No lipoprotein	EchoLocation
ytfM	16132042	predicted outer membrane protein and surface antigen	Signal peptide	Signal peptide	No alpha-helix	outer membrane	p>0,01	0,013	1	2,944	Beta-barrel	No lipoprotein	Stegmeier, 2007
yfaL	16130168	adhesin	Signal peptide	Signal peptide	No alpha-helix	outer membrane	5,90E-05	0,042	-	2,902	Beta-barrel	No lipoprotein	Marani 2006
yfaZ	90111407	predicted outer membrane porin protein	Signal peptide	Signal peptide	No alpha-helix	unknown	p>0,01	0,063	1	2,775	Beta-barrel	No lipoprotein	Marani 2006
matB	16128278	conserved protein	Signal peptide	Signal peptide	No alpha-helix	unknown	p>0,01	0,063	-	2,867	Beta-barrel	No lipoprotein	Marani 2006
nanC	90111728	N-acetylnuraminic acid outer membrane channel protein	Signal peptide	Signal peptide	No alpha-helix	outer membrane	p>0,01	0,035	3	2,93	Beta-barrel	No lipoprotein	Condemine 2005
nfrA	16128551	bacteriophage N4 receptor, outer membrane subunit	Signal peptide	Signal peptide	No alpha-helix	outer membrane	p>0,01	0,02	2	>2,965	Beta-barrel	No lipoprotein	Kiino 1993
bhsA	16129075	predicted protein	Signal peptide	Signal peptide	No alpha-helix	unknown	p>0,01	0,013	-	2,913	No beta-barrel	No lipoprotein	Zhang 2007
ypjA	90111474	adhesin-like autotransporter	Signal peptide	Signal peptide	1 alpha-helix	unknown	0,0068	0,04	4	2,898	Beta-barrel	No lipoprotein	Roux 2005
ycgV	16129165	predicted adhesin			No alpha-helix	unknown	0,0049	0,059	1	2,904	Beta-barrel	No lipoprotein	Roux, 2005
ydiY	16129676	conserved protein	Signal peptide	Signal peptide	No alpha-helix	outer membrane	p>0,01	0,057	1	2,871	Beta-barrel	No lipoprotein	
yagX	16128276	predicted aromatic compound dioxygenase	Signal peptide	Signal peptide	No alpha-helix	outer membrane	p>0,01	0,045	1	2,931	Beta-barrel	No lipoprotein	
yddB	16129454	predicted porin protein	Signal peptide	Signal peptide	No alpha-helix	outer membrane	p>0,01	0,039	4	2,959	Beta-barrel	No lipoprotein	
yfeN	16130334	conserved outer membrane protein	Signal peptide	Signal peptide	No alpha-helix	cytoplasmic membrane	p>0,01	0,033	5	2,914	Beta-barrel	No lipoprotein	
yjbE	16131852	predicted protein	Signal peptide	Signal peptide	No alpha-helix	unknown	p>0,01	0,07	-	2,707	No beta-barrel	No lipoprotein	
yjbG	16131854	conserved protein	Signal peptide	Signal peptide	No alpha-helix	unknown	p>0,01	0,019	-	2,908	No beta-barrel	No lipoprotein	
gfcD	16128950	conserved protein	Signal peptide	Signal peptide	No alpha-helix	outer membrane	p>0,01	0,015	1	>2,965	Beta-barrel	No lipoprotein	
gfcC	16128951	conserved protein	Signal peptide	Signal peptide	No alpha-helix	unknown	p>0,01	0,017	-	2,956	No beta-barrel	No lipoprotein	
pgaA	16128988	predicted outer membrane protein	Signal peptide	Signal peptide	No alpha-helix	unknown	p>0,01	0,009	2	>2,965	Beta-barrel	No lipoprotein	
ytfN	16132043	conserved protein	Signal peptide	Signal peptide	1 alpha-helix	outer membrane	0,0094	0,016	2	2,907	Beta-barrel	No lipoprotein	
yhcD	16131106	predicted outer membrane protein	Non-secretory protein	Signal peptide	No alpha-helix	outer membrane	0,00095	0,032	1	2,939	Beta-barrel	No lipoprotein	
htrE	16128132	predicted outer membrane usher protein	Non-secretory protein	Signal peptide	No alpha-helix	outer membrane	0,0056	0,04	3	2,949	Beta-barrel	No lipoprotein	
yncD	16129410	predicted iron outer membrane transporter	Signal peptide	Signal peptide	No alpha-helix	outer membrane	p>0,01	0,037	1	2,931	Beta-barrel	No lipoprotein	
ycbS	16128907	predicted outer membrane usher protein	Signal peptide	Signal peptide	1 alpha-helix	outer membrane	p>0,01	0,044	3	2,92	Beta-barrel	No lipoprotein	
ybfM	16128657	predicted otuer membrane porin	Non-secretory protein	Signal peptide	No alpha-helix	outer membrane	p>0,01	0,021	3	2,939	Beta-barrel	No lipoprotein	
sfmD	16128516	predicted outer membrane usher protein	Signal peptide	Signal peptide	1 alpha-helix	outer membrane	p>0,01	0,045	3	2,956	Beta-barrel	No lipoprotein	
yehB	16130047	predicted outer membrane protein	Signal peptide	Signal peptide	No alpha-helix	outer membrane	p>0,01	0,04	3	2,956	Beta-barrel	No lipoprotein	

yraJ	16131036	predicted outer membrane protein	Non-secretory protein	Signal peptide	No alpha-helix	outer membrane	p>0,01	0,047	3	2,962	Beta-barrel	No lipoprotein	
yiaT	16131455	predicted protein	Signal peptide	Signal peptide	No alpha-helix	outer membrane	p>0,01	0,027	2	2,92	Beta-barrel	No lipoprotein	
ybgQ	90111165	predicted outer membrane protein	Signal peptide	Signal peptide	No alpha-helix	outer membrane	p>0,01	0,033	-	2,952	Beta-barrel	No lipoprotein	
yadN	16128134	predicted fimbrial-like adhesin protein	Signal peptide	Signal peptide	No alpha-helix	unknown	0,0063	0,041	-	2,915	No beta-barrel	No lipoprotein	
yraK	16131037	predicted fimbrial-like adhesin protien	Signal peptide	Signal peptide	No alpha-helix	unknown	0,0073	0,007	-	2,959	Beta-barrel	No lipoprotein	
ycgK	16129141	predicted protein	Signal peptide	Signal peptide	No alpha-helix	unknown	p>0,01	0,019	1	2,88	Beta-barrel	No lipoprotein	
yehA	16130046	predicted fimbrial-like adhesin protein	Signal peptide	Signal peptide	No alpha-helix	outer membrane	p>0,01	0,023	1	2,912	Beta-barrel	No lipoprotein	
yfaP	16130162	conserved protein	Signal peptide	Signal peptide	No alpha-helix	unknown	p>0,01	0,018	2	2,946	Beta-barrel	No lipoprotein	
ycbQ	90111190	predicted fimbrial-like adhesin protein	Signal peptide	Signal peptide	No alpha-helix	extracellular	p>0,01	0,016	-	2,91	Beta-barrel	No lipoprotein	
ygiL	16130939	predicted fimbrial-like adhesin protein	Signal peptide	Signal peptide	No alpha-helix	extracellular	p>0,01	0,008	-	2,931	Beta-barrel	No lipoprotein	
ydeQ	16129461	predicted fimbrial-like adhesin protein	Signal peptide	Signal peptide	No alpha-helix	unknown	p>0,01	0,024	-	2,933	Beta-barrel	No lipoprotein	
yadL	16128130	predicted fimbrial-like adhesin protein	Signal peptide	Signal peptide	1 alpha-helix	unknown	p>0,01	0,019	-	2,934	Beta-barrel	No lipoprotein	
yehD	16130049	predicted fimbrial-like adhesin protein	Signal peptide	Signal peptide	1 alpha-helix	unknown	p>0,01	0,027	-	2,943	Beta-barrel	No lipoprotein	
yagW	16128275	predicted receptor	Signal peptide	Signal peptide	No alpha-helix	unknown	p>0,01	0,013	1	>2,965	Beta-barrel	No lipoprotein	
ycbT	16128908	predicted fimbrial-like adhesin protein	Signal peptide	Signal peptide	1 alpha-helix	unknown	p>0,01	<0	-	2,947	Beta-barrel	No lipoprotein	
ycbV	90111191	predicted fimbrial-like adhesin protein	Signal peptide	Signal peptide	No alpha-helix	unknown	p>0,01	<0	-	2,962	Beta-barrel	No lipoprotein	
ydeR	16129462	predicted fimbrial-like adhesin protein	Signal peptide	Signal peptide	No alpha-helix	unknown	p>0,01	0,025	-	>2,965	Beta-barrel	No lipoprotein	
yadC	16128128	predicted fimbrial-like adhesin protein	Signal peptide	Signal peptide	1 alpha-helix	unknown	p>0,01	0,007	-	>2,965	Beta-barrel	No lipoprotein	
yfcP	16130268	predicted fimbrial-like adhesin protein	Signal peptide	Signal peptide	1 alpha-helix	unknown	p>0,01	0,016	-	2,872	No beta-barrel	No lipoprotein	
yraH	16131034	predicted fimbrial-like adhesin protein	Signal peptide	Signal peptide	No alpha-helix	extracellular	p>0,01	0,004	-	2,901	No beta-barrel	No lipoprotein	
hofQ	16131268	predicted fimbrial transporter	Signal peptide	Signal peptide	No alpha-helix	outer membrane	p>0,01	0,017	-	2,909	No beta-barrel	No lipoprotein	
yfcR	16130270	predicted fimbrial-like adhesin protein	Signal peptide	Signal peptide	No alpha-helix	unknown	p>0,01	0,019	-	2,936	No beta-barrel	No lipoprotein	
ybgD	16128694	predicted fimbrial-like adhesin protein	Signal peptide	Signal peptide	No alpha-helix	extracellular	p>0,01	0,017	-	2,963	No beta-barrel	No lipoprotein	
ygjJ	16130974	conserved protein	Signal peptide	Signal peptide	No alpha-helix	unknown	p>0,01	<0	1	>2,965	Beta-barrel	No lipoprotein	
yacH	16128110	predicted protein	Signal peptide	Signal peptide	No alpha-helix	unknown	p>0,01	0,004	-	>2,965	Beta-barrel	No lipoprotein	
yfjT	16130554	CP4-57 prophage; predicted protein	Signal peptide	Signal peptide	No alpha-helix	unknown	p>0,01	0,004	-	>2,965	Beta-barrel	No lipoprotein	
ykfB	16128235	CP4-6 prophage; predicted protein	Signal peptide	Signal peptide	No alpha-helix	unknown	p>0,01	0,013	-	>2,965	Beta-barrel	No lipoprotein	
yjfN	90111700	predicted protein	Non-secretory protein	Signal peptide	No alpha-helix	unknown	p>0,01	0,003	-	2,879	No beta-barrel	No lipoprotein	
yeeJ	145698281	adhesin	Non-secretory protein	Signal peptide	No alpha-helix	outer membrane	0,0025	0,045	-	2,902	Beta-barrel	No lipoprotein	
ychO	145698248	predicted invasin	Signal peptide	Signal peptide	No alpha-helix	outer membrane	p>0,01	0,049	-	>2,965	Beta-barrel	No lipoprotein	
flgK	16129045	flagellar hook-filament junction protein 1	Non-secretory protein	Non-secretory protein	No alpha-helix	outer membrane	p>0,01	0,062	-	2,895	No beta-barrel	No lipoprotein	
flgL	16129046	flagellar hook-filament junction protein	Non-secretory protein	Non-secretory protein	No alpha-helix	outer membrane	p>0,01	0,04	-	2,93	No beta-barrel	No lipoprotein	

ydgH	16129562	predicted protein	Signal peptide	Signal peptide	No alpha-helix	unknown	p>0,01	0,031	-	2,929	Beta-barrel	No lipoprotein	
bcsC	145698324	cellulose synthase subunit	Non-secretory protein	Non-secretory protein	No alpha-helix	outer membrane	p>0,01	0,033	-	2,96	Beta-barrel	No lipoprotein	
**False positives**													
amiB	16131991	N-acetylmuramoyl-L-alanine amidase II	Signal peptide	Signal peptide	No alpha-helix	unknown	p>0,01	0,005	-	2,916	No beta-barrel	No lipoprotein	EchoLocation
csgA	16129005	cryptic curlin major subunit	Signal peptide	Signal peptide	No alpha-helix	unknown	p>0,01	0,086	-	2,874	No beta-barrel	No lipoprotein	EchoLocation
**False negatives**													
rffD	49176410	UDP-N-acetyl-D-mannosaminuronic acid dehydrogenase	Signal peptide	Signal peptide	No alpha-helix	cytoplasmic	p>0,01	<0	-	>2,965	No beta-barrel	No lipoprotein	EchoLocation
yliI	16128805	predicted dehydrogenase	Signal peptide	Signal peptide	No alpha-helix	unknown	p>0,01	<0	-	>2,965	No beta-barrel	No lipoprotein	Marani 2007
yaiO	16128343	predicted protein	Signal peptide	Signal peptide	1 alpha-helix	unknown	p>0,01	<0	-	>2,965	No beta-barrel	No lipoprotein	Marani 2007
**Outer membrane lipoproteins**													
gfcE	16128949	predicted exopolysaccharide export protein	Signal peptide	Signal peptide	No alpha-helix	outer membrane	p>0,01	<0	-	>2,965	No beta-barrel	Outer membrane lipoprotein	EchoLocation
yfiB	16130526	predicted outer membrane lipoprotein	Signal peptide	Signal peptide	No alpha-helix	outer membrane	p>0,01	<0	-	>2,965	No beta-barrel	Outer membrane lipoprotein	EchoLocation
borD	16128540	DLP12 prophage; predicted lipoprotein	Signal peptide	Signal peptide	No alpha-helix	unknown	p>0,01	<0	-	>2,965	No beta-barrel	Outer membrane lipoprotein	EchoLocation
ecnA	49176462	entericidin A membrane lipoprotein, antidote entericidin B	Signal peptide	Signal peptide	No alpha-helix	unknown	p>0,01	<0	-	>2,965	No beta-barrel	Outer membrane lipoprotein	EchoLocation
gfcB	16128952	predicted outer membrane lipoprotein	Signal peptide	Signal peptide	No alpha-helix	unknown	p>0,01	<0	-	>2,965	No beta-barrel	Outer membrane lipoprotein	EchoLocation
hslJ	16129340	heat-inducible protein	Signal peptide	Signal peptide	No alpha-helix	unknown	p>0,01	<0	-	>2,965	No beta-barrel	Outer membrane lipoprotein	EchoLocation
mltC	90111520	membrane-bound lytic murein transglycosylase C	Signal peptide	Signal peptide	No alpha-helix	unknown	p>0,01	<0	-	>2,965	No beta-barrel	Outer membrane lipoprotein	EchoLocation
nleP	16128185	lipoprotein involved with copper homeostasis and adhesion	Signal peptide	Signal peptide	No alpha-helix	unknown	p>0,01	<0	-	>2,965	No beta-barrel	Outer membrane lipoprotein	EchoLocation
rcsF	16128189	predicted outer membrane protein, signal	Signal peptide	Signal peptide	No alpha-helix	unknown	p>0,01	<0	-	>2,965	No beta-barrel	Outer membrane lipoprotein	EchoLocation
spr	16130113	predicted peptidase, outer membrane lipoprotein	Signal peptide	Signal peptide	No alpha-helix	unknown	p>0,01	<0	-	>2,965	No beta-barrel	Outer membrane lipoprotein	EchoLocation
vacJ	16130279	predicted lipoprotein	Signal peptide	Signal peptide	No alpha-helix	unknown	p>0,01	<0	-	>2,965	No beta-barrel	Outer membrane lipoprotein	EchoLocation
ybfN	16128658	predicted lipoprotein	Signal peptide	Signal peptide	No alpha-helix	unknown	p>0,01	<0	-	>2,965	No beta-barrel	Outer membrane lipoprotein	EchoLocation
ybjP	16128833	predicted lipoprotein	Signal peptide	Signal peptide	No alpha-helix	unknown	p>0,01	<0	-	>2,965	No beta-barrel	Outer membrane lipoprotein	EchoLocation
yceB	16129026	predicted lipoprotein	Signal peptide	Signal peptide	No alpha-helix	unknown	p>0,01	<0	-	>2,965	No beta-barrel	Outer membrane lipoprotein	EchoLocation
ydcL	16129390	predicted lipoprotein	Signal peptide	Signal peptide	No alpha-helix	unknown	p>0,01	<0	-	>2,965	No beta-barrel	Outer membrane lipoprotein	EchoLocation
yecR	16129854	predicted protein	Signal peptide	Signal peptide	No alpha-helix	unknown	p>0,01	<0	-	>2,965	No beta-barrel	Outer membrane lipoprotein	EchoLocation
yedD	16129875	predicted protein	Signal peptide	Signal peptide	No alpha-helix	unknown	p>0,01	<0	-	>2,965	No beta-barrel	Outer membrane lipoprotein	EchoLocation
yfiO	16130516	predicted lipoprotein	Signal peptide	Signal peptide	No alpha-helix	unknown	p>0,01	<0	-	>2,965	No beta-barrel	Outer membrane lipoprotein	EchoLocation
yfhL	16131247	conserved secreted peptide	Signal peptide	Signal peptide	No alpha-helix	unknown	p>0,01	<0	-	>2,965	No beta-barrel	Outer membrane lipoprotein	EchoLocation

yidQ	90111638	conserved outer membrane protein	Signal peptide	Signal peptide	No alpha-helix	unknown	p>0,01	<0	-	>2,965	No beta-barrel	Outer membrane lipoprotein	EchoLocation
nlpC	16129664	predicted lipoprotein	Signal peptide	Non-secretory protein	No alpha-helix	unknown	p>0,01	<0	-	>2,965	No beta-barrel	Outer membrane lipoprotein	EchoLocation
yoaF	16129747	conserved outer membrane protein	Signal peptide	Non-secretory protein	No alpha-helix	unknown	p>0,01	<0	-	>2,965	No beta-barrel	Outer membrane lipoprotein	EchoLocation
yhdV	16131155	predicted outer membrane protein	Signal peptide	Signal peptide	1 alpha-helix	unknown	p>0,01	<0	-	>2,965	No beta-barrel	Outer membrane lipoprotein	EchoLocation
yghH	16130912	predicted outer membrane lipoprotein	Signal peptide	Signal peptide	1 alpha-helix	unknown	p>0,01	<0	-	>2,965	No beta-barrel	Outer membrane lipoprotein	EchoLocation
mltB	16130608	membrane-bound lytic murein transglycosylase B	Signal peptide	Signal peptide	No alpha-helix	cytoplasmic membrane	p>0,01	<0	-	>2,965	No beta-barrel	Outer membrane lipoprotein	EchoLocation
mltA	16130720	membrane-bound lytic murein transglycosylase A	Signal peptide	Signal peptide	No alpha-helix	unknown	p>0,01	0,004	-	>2,965	No beta-barrel	Outer membrane lipoprotein	EchoLocation
yjbF	90111676	predicted lipoprotein	Signal peptide	Signal peptide	No alpha-helix	unknown	p>0,01	0,004	-	>2,965	No beta-barrel	Outer membrane lipoprotein	EchoLocation
lolB	16129172	chaperone for lipoproteins	Signal peptide	Signal peptide	No alpha-helix	unknown	p>0,01	0,012	-	>2,965	No beta-barrel	Outer membrane lipoprotein	EchoLocation
yddW	16129450	predicted lipoprotein	Signal peptide	Signal peptide	No alpha-helix	unknown	p>0,01	<0	-	>2,965	Beta-barrel	Outer membrane lipoprotein	EchoLocation
yfeY	16130357	predicted protein	Signal peptide	Signal peptide	No alpha-helix	unknown	p>0,01	<0	-	>2,965	Beta-barrel	Outer membrane lipoprotein	EchoLocation
yeaY	16129760	predicted lipoprotein	Signal peptide	Non-secretory protein	No alpha-helix	unknown	p>0,01	<0	-	>2,965	Beta-barrel	Outer membrane lipoprotein	EchoLocation
yfiL	90111465	predicted protein	Signal peptide	Non-secretory protein	No alpha-helix	unknown	p>0,01	<0	-	>2,965	Beta-barrel	Outer membrane lipoprotein	EchoLocation
yjeI	90111692	conserved protein	Signal peptide	Signal peptide	No alpha-helix	unknown	p>0,01	<0	-	2,913	No beta-barrel	Outer membrane lipoprotein	EchoLocation
emtA	90111231	lytic murein endotransglycosylase E	Signal peptide	Signal peptide	No alpha-helix	unknown	p>0,01	<0	-	2,927	No beta-barrel	Outer membrane lipoprotein	EchoLocation
ynbE	16129343	predicted lipoprotein	Signal peptide	Signal peptide	No alpha-helix	unknown	p>0,01	<0	-	2,927	No beta-barrel	Outer membrane lipoprotein	EchoLocation
ycaL	90111186	predicted peptidase with chaperone function	Signal peptide	Signal peptide	No alpha-helix	unknown	p>0,01	<0	-	2,935	No beta-barrel	Outer membrane lipoprotein	EchoLocation
yfgH	16130430	predicted outer membrane lipoprotein	Signal peptide	Signal peptide	No alpha-helix	unknown	p>0,01	<0	-	2,944	No beta-barrel	Outer membrane lipoprotein	EchoLocation
csgG	16129000	outer membrane lipoprotein	Signal peptide	Signal peptide	No alpha-helix	unknown	p>0,01	<0	-	2,948	No beta-barrel	Outer membrane lipoprotein	EchoLocation
wza	16130002	lipoprotein required for capsular polysaccharide translocaion through the outer membrane	Signal peptide	Signal peptide	No alpha-helix	outer membrane	p>0,01	<0	2	>2,965	No beta-barrel	Outer membrane lipoprotein	EchoLocation
blc	16131974	outer membrane lipoprotein (lipocalin)	Signal peptide	Signal peptide	No alpha-helix	unknown	p>0,01	<0	-	>2,965	Beta-barrel	Outer membrane lipoprotein	EchoLocation
yafT	16128203	predicted aminopeptidase	Signal peptide	Signal peptide	No alpha-helix	unknown	p>0,01	<0	-	>2,965	Beta-barrel	Outer membrane lipoprotein	EchoLocation
slyB	49176129	outer membrane lipoprotein	Signal peptide	Signal peptide	No alpha-helix	unknown	p>0,01	0,037	-	2,841	No beta-barrel	Outer membrane lipoprotein	EchoLocation
yiaD	49176370	predicted outer membrane lipoprotein	Signal peptide	Signal peptide	3 alpha-helices	outer Membrane	p>0,01	0,034	-	2,842	No beta-barrel	Outer membrane lipoprotein	EchoLocation
ybaY	49176025	predicted outer membrane lipoprotein	Signal peptide	Signal peptide	No alpha-helix	unknown	p>0,01	0,009	-	2,857	No beta-barrel	Outer membrane lipoprotein	EchoLocation
cusC	16128555	copper/silver efflux system, outer membrane component	Signal peptide	Non-secretory protein	No alpha-helix	outer membrane	p>0,01	0,047	-	2,877	No beta-barrel	Outer membrane lipoprotein	EchoLocation
nlpB	90111442	lipoprotein	Signal peptide	Signal peptide	No alpha-helix	unknown	p>0,01	0,039	-	2,907	No beta-barrel	Outer membrane lipoprotein	EchoLocation

flgH	16129042	flagellar protein of basal-body outer-membrane L ring	Signal peptide	Signal peptide	No alpha-helix	unknown	p>0,01	0,041	-	2,913	No beta-barrel	Outer membrane lipoprotein	EchoLocation
yfgL	16130437	protein assembly complex, lipoprotein component	Signal peptide	Signal peptide	No alpha-helix	unknown	p>0,01	0,007	-	2,92	No beta-barrel	Outer membrane lipoprotein	EchoLocation
nlpD	16130649	predicted outer membrane lipoprotein	Signal peptide	Signal peptide	No alpha-helix	unknown	p>0,01	0,033	-	2,926	No beta-barrel	Outer membrane lipoprotein	EchoLocation
ycfM	16129068	predicted outer membrane lipoprotein	Signal peptide	Signal peptide	No alpha-helix	unknown	p>0,01	0,008	-	2,93	No beta-barrel	Outer membrane lipoprotein	EchoLocation
lpp	16129633	murein lipoprotein	Signal peptide	Signal peptide	No alpha-helix	unknown	p>0,01	0,007	-	2,935	No beta-barrel	Outer membrane lipoprotein	EchoLocation
pal	16128716	peptidoglycan-associated outer membrane lipoprotein	Signal peptide	Signal peptide	No alpha-helix	outer membrane	p>0,01	0,001	-	2,937	No beta-barrel	Outer membrane lipoprotein	EchoLocation
yajG	90111134	predicted lipoprotein	Signal peptide	Signal peptide	No alpha-helix	unknown	p>0,01	0,009	-	2,943	No beta-barrel	Outer membrane lipoprotein	EchoLocation
yghG	16130871	predicted protein	Signal peptide	Signal peptide	No alpha-helix	unknown	p>0,01	0,003	-	2,958	No beta-barrel	Outer membrane lipoprotein	EchoLocation
slp	90111603	outer membrane lipoprotein	Signal peptide	Signal peptide	No alpha-helix	unknown	p>0,01	0,007	-	>2,965	Beta-barrel	Outer membrane lipoprotein	EchoLocation
yehR	90111391	conserved protein	Signal peptide	Signal peptide	No alpha-helix	unknown	p>0,01	<0	-	2,958	Beta-barrel	Outer membrane lipoprotein	EchoLocation
ybhC	16128740	predicted pectinesterase	Signal peptide	Signal peptide	No alpha-helix	unknown	0,00026	0,033	-	>2,965	Beta-barrel	Outer membrane lipoprotein	EchoLocation
yceK	16129013	predicted lipoprotein	Signal peptide	Signal peptide	No alpha-helix	unknown	p>0,01	<0	-	>2,965	No beta-barrel	Outer membrane lipoprotein	EchoLocation
rlpB	16128624	minor lipoprotein	Signal peptide	Signal peptide	No alpha-helix	unknown	p>0,01	<0	-	>2,965	No beta-barrel	Outer membrane lipoprotein	EchoLocation
rlpA	16128616	minor lipoprotein	Signal peptide	Signal peptide	No alpha-helix	unknown	p>0,01	<0	-	>2,965	No beta-barrel	Outer membrane lipoprotein	EchoLocation
pgaB	16128987	predicted enzyme associated with biofilm formation	Signal peptide	Signal peptide	No alpha-helix	unknown	p>0,01	<0	-	>2,965	No beta-barrel	Outer membrane lipoprotein	EchoLocation
osmB	16129244	lipoprotein	Signal peptide	Signal peptide	No alpha-helix	unknown	p>0,01	<0	-	>2,965	No beta-barrel	Outer membrane lipoprotein	EchoLocation
nlpI	16131055	conserved protein	Signal peptide	Signal peptide	No alpha-helix	unknown	p>0,01	<0	-	>2,965	No beta-barrel	Outer membrane lipoprotein	EchoLocation
yraP	16131042	predicted protein	Signal peptide	Signal peptide	No alpha-helix	Periplasmic	p>0,01	<0	-	>2,965	No beta-barrel	Outer membrane lipoprotein	EchoLocation
ycjN	16129271	predicted sugar transporter subunit: periplasmic-binding component of ABC system	Signal peptide	Signal peptide	No alpha-helix	Periplasmic	p>0,01	<0	-	>2,965	No beta-barrel	Outer membrane lipoprotein	EchoLocation
ybfP	16128665	predicted protein	Signal peptide	Signal peptide	1 alpha-helix	unknown	p>0,01	<0	-	>2,965	No beta-barrel	Outer membrane lipoprotein	EchoLocation
ygdI	90111491	predicted protein	Signal peptide	Signal peptide	1 alpha-helix	unknown	p>0,01	<0	-	>2,965	No beta-barrel	Outer membrane lipoprotein	EchoLocation
ygdR	16130737	predicted protein	Signal peptide	Signal peptide	1 alpha-helix	unknown	p>0,01	<0	-	>2,965	No beta-barrel	Outer membrane lipoprotein	EchoLocation
yifL	94541130	predicted lipoprotein	Signal peptide	Signal peptide	1 alpha-helix	unknown	p>0,01	<0	-	>2,965	No beta-barrel	Outer membrane lipoprotein	EchoLocation
ecnB	49176463	entericidin B membrane lipoprotein	Signal peptide	Signal peptide	1 alpha-helix	unknown	p>0,01	<0	-	>2,965	No beta-barrel	Outer membrane lipoprotein	EchoLocation
smpA	90111468	small membrane lipoprotein	Signal peptide	Signal peptide	No alpha-helix	unknown	p>0,01	0,022	-	>2,965	No beta-barrel	Outer membrane lipoprotein	Sklar 2007
yfhG	16130480	conserved protein	Signal peptide	Signal peptide	No alpha-helix	unknown	p>0,01	<0	-	>2,965	No beta-barrel	Outer membrane lipoprotein	
yggG	90111514	predicted peptidase	Signal peptide	Signal peptide	No alpha-helix	unknown	p>0,01	0,001	-	2,943	No beta-barrel	Outer membrane lipoprotein	

ydeK	16129469	predicted lipoprotein	Non-secretory protein	Signal peptide	No alpha-helix	outer membrane	0,0031	0,055	-	2,876	No beta-barrel	Outer membrane lipoprotein	
ydhA	90111309	predicted lipoprotein	Signal peptide	Signal peptide	No alpha-helix	unknown	p>0,01	<0	-	>2,965	No beta-barrel	Outer membrane lipoprotein	
yidX	49176393	predicted lipoprotein C	Signal peptide	Signal peptide	1 alpha-helix	unknown	p>0,01	<0	-	>2,965	No beta-barrel	Outer membrane lipoprotein	
yjbH	16131855	predicted porin	Signal peptide	Signal peptide	No alpha-helix	outer membrane	p>0,01	0,033	1	2,955	Beta-barrel	Outer membrane lipoprotein	
rzoD	94541098	DLP12 prophage; predicted lipoprotein	Signal peptide	Signal peptide	No alpha-helix	unknown	p>0,01	<0	-	>2,965	No beta-barrel	Outer membrane lipoprotein	
rzoR	94541110	Rac prophage; predicted lipoprotein	Signal peptide	Signal peptide	No alpha-helix	unknown	p>0,01	<0	-	>2,965	No beta-barrel	Outer membrane lipoprotein	
yegR	90111383	predicted protein	Signal peptide	Signal peptide	No alpha-helix	unknown	p>0,01	<0	-	>2,965	No beta-barrel	Outer membrane lipoprotein	
yaeF	90111098	predicted lipoprotein	Signal peptide	Non-secretory protein	No alpha-helix	unknown	p>0,01	<0	-	>2,965	No beta-barrel	Outer membrane lipoprotein	
ybeT	16128630	conserved outer membrane protein	Signal peptide	Signal peptide	1 alpha-helix	unknown	p>0,01	<0	-	>2,965	No beta-barrel	Outer membrane lipoprotein	
ybbC	16128482	predicted protein	Signal peptide	Signal peptide	No alpha-helix	unknown	p>0,01	<0	-	>2,965	Beta-barrel	Outer membrane lipoprotein	
yjaH	16131831	conserved protein	Signal peptide	Signal peptide	No alpha-helix	unknown	p>0,01	<0	-	>2,965	Beta-barrel	Outer membrane lipoprotein	
ydbJ	94541111	predicted protein	Signal peptide	Signal peptide	No alpha-helix	unknown	p>0,01	<0	-	2,909	No beta-barrel	Outer membrane lipoprotein	
mdtP	16131906	predicted outer membrane factor of efflux pump	Signal peptide	Signal peptide	No alpha-helix	outer membrane	p>0,01	0,012	-	2,922	No beta-barrel	Outer membrane lipoprotein	
yajI	90111129	predicted lipoprotein	Signal peptide	Signal peptide	No alpha-helix	unknown	p>0,01	0,031	-	2,936	No beta-barrel	Outer membrane lipoprotein	
ypdI	16130308	predicted lipoprotein involved in colanic acid biosynthesis	Signal peptide	Signal peptide	1 alpha-helix	unknown	p>0,01	<0	-	>2,965	No beta-barrel	Outer membrane lipoprotein	
yecT	90111353	predicted protein	Signal peptide	Signal peptide	No alpha-helix	unknown	p>0,01	<0	-	>2,965	No beta-barrel	No lipoprotein	
yraM	16131039	coserved protein	Signal peptide	Signal peptide	No alpha-helix	outer membrane	p>0,01	0,021	-	2,899	No beta-barrel	Outer membrane lipoprotein	
yfbK	16130205	conserved protein	Signal peptide	Signal peptide	No alpha-helix	unknown	p>0,01	0,014	-	>2,965	No beta-barrel	Outer membrane lipoprotein	
ygiB	145698308	conserved outer membrane protein	Non-secretory protein	Signal peptide	No alpha-helix	unknown	p>0,01	<0	-	>2,965	No beta-barrel	Outer membrane lipoprotein	
ymbA	145698237	conserved protein	Signal peptide	Signal peptide	No alpha-helix	unknown	p>0,01	0,032	-	>2,965	No beta-barrel	Outer membrane lipoprotein	
yfiM	145698302	predicted protein	Signal peptide	Signal peptide	No alpha-helix	unknown	p>0,01	<0	-	>2,965	Beta-barrel	Outer membrane lipoprotein	
ysaB	94541126	predicted protein	Signal peptide	Signal peptide	No alpha-helix	unknown	p>0,01	<0	-	>2,965	No beta-barrel	Outer membrane lipoprotein	
ycfL	16129067	predicted protein	Signal peptide	Signal peptide	No alpha-helix	unknown	p>0,01	<0	-	>2,965	No beta-barrel	Outer membrane lipoprotein	
yjfO	90111701	conserved protein	Signal peptide	Signal peptide	1 alpha-helix	unknown	p>0,01	<0	-	>2,965	No beta-barrel	Outer membrane lipoprotein	
ydhO	16129613	predicted lipoprotein	Signal peptide	Signal peptide	No alpha-helix	unknown	p>0,01	<0	-	>2,965	No beta-barrel	No lipoprotein	
yafL	16128213	predicted lipoprotein and C40 family peptidase	Signal peptide	Signal peptide	1 alpha-helix	unknown	p>0,01	<0	-	>2,965	No beta-barrel	No lipoprotein	
yiaF	90111615	conserved protein	Signal peptide	Non-secretory protein	1 alpha-helix	unknown	p>0,01	<0	-	>2,965	No beta-barrel	Outer membrane lipoprotein	
**False negatives**													

osmE	16129693	DNA-binding transcriptional activator	Signal peptide	Signal peptide	1 alpha-helix	unknown	p>0,01	<0	-	>2,965	No beta-barrel	Outer membrane lipoprotein	EchoLocation
yqJA	16130990	conserved inner membrane protein	Signal peptide	Non-secretory protein	6 alpha-helices	cytoplasmic membrane	p>0,01	<0	-	>2,965	No beta-barrel		EchoLocation
ygeR	90111503	tetratricopeptide repeat transcriptional regulator	Signal peptide	Signal peptide	No alpha-helix	unknown	p>0,01	<0	-	>2,965	No beta-barrel	Outer membrane lipoprotein	EchoLocation
ybjR	16128835	predicted amidase and lipoprotein	Signal peptide	Signal peptide	No alpha-helix	unknown	p>0,01	<0	-	>2,965	No beta-barrel	Outer membrane lipoprotein	EchoLocation
mtlD	16128198	predicted membrane-bound lytic murein transglycosylase D	Signal peptide	Signal peptide	No alpha-helix	unknown	p>0,01	<0	-	>2,965	No beta-barrel	Outer membrane lipoprotein	EchoLocation
**cell wall components**													
csgE	16129002	predicted transport protein	Signal peptide	Signal peptide	No alpha-helix	unknown	p>0,01	<0	2	>2,965	No beta-barrel	No lipoprotein	EchoLocation
pbpC	16130444	fused transglycosylase/transpeptidase	Signal peptide	Signal peptide	1 alpha-helix	cytoplasmic membrane	p>0,01	<0	-	2,96	No beta-barrel	No lipoprotein	EchoLocation
amiC	90111492	N-acetylmuramoyl-L-alanine amidase	Non-secretory protein	Signal peptide	No alpha-helix	unknown	p>0,01	<0	-	2,963	No beta-barrel	No lipoprotein	EchoLocation
skp	16128171	periplasmic chaperone	Signal peptide	Signal peptide	No alpha-helix	outer membrane	p>0,01	0,022	-	>2,965	No beta-barrel	No lipoprotein	EchoLocation
ynfD	90111300	predicted protein	Signal peptide	Signal peptide	No alpha-helix	unknown	p>0,01	<0	-	2,937	No beta-barrel	No lipoprotein	EchoLocation
yfdX	16130307	predicted protein	Signal peptide	Signal peptide	No alpha-helix	unknown	p>0,01	<0	-	2,94	No beta-barrel	No lipoprotein	EchoLocation
amiA	16130360	Nacetylmuramoyl-l-alanine amidase I	Signal peptide	Signal peptide	No alpha-helix	cytoplasmic membrane	p>0,01	<0	-	>2,965	No beta-barrel	No lipoprotein	EchoLocation
dacA	16128615	D-alanyl-D-alanine carboxypeptidase (penicillin-binding protein 5)	Signal peptide	Signal peptide	No alpha-helix	cytoplasmic membrane	p>0,01	<0	-	>2,965	No beta-barrel	No lipoprotein	EchoLocation
dacC	16128807	D-alanyl-D-alanine carboxypeptidase (penicillin-binding protein 6a)	Signal peptide	Signal peptide	No alpha-helix	cytoplasmic membrane	p>0,01	<0	-	>2,965	No beta-barrel	No lipoprotein	EchoLocation
dacD	90111369	D-alanyl-D-alanine carboxypeptidase (penicillin-binding protein 6b)	Signal peptide	Signal peptide	No alpha-helix	cytoplasmic membrane	p>0,01	<0	-	>2,965	No beta-barrel	No lipoprotein	EchoLocation
pbpG	90111393	D-alanyl-D-alanine endopeptidase	Signal peptide	Signal peptide	No alpha-helix	Periplasmic	p>0,01	<0	-	>2,965	No beta-barrel	No lipoprotein	EchoLocation
ampC	16131975	beta-lactamase/D-alanine carboxypeptidase	Signal peptide	Signal peptide	No alpha-helix	Periplasmic	p>0,01	<0	-	>2,965	No beta-barrel	No lipoprotein	EchoLocation
yggN	16130859	predicted protein	Signal peptide	Signal peptide	No alpha-helix	unknown	p>0,01	<0	-	>2,965	No beta-barrel	No lipoprotein	EchoLocation
ddlA	16128366	D-alanina-D-alanine ligase A	Non-secretory protein	Non-secretory protein	No alpha-helix	cytoplasmic	p>0,01	<0	-	2,942	No beta-barrel	No lipoprotein	Zawadzke 1991
iap	16130660	aminopeptidase in alkaline phosphatase isozyme conversion	Signal peptide	Signal peptide	No alpha-helix	unknown	p>0,01	<0	-	>2,965	No beta-barrel	No lipoprotein	Bucevic-Popovic 2004
ampH	16128361	beta-lactamase/D-alanine carboxypeptidase	Signal peptide	Signal peptide	No alpha-helix	Periplasmic	p>0,01	<0	-	>2,965	No beta-barrel	No lipoprotein	Henderson 1997
yfeW	90111432	predicted periplasmic esterase	Signal peptide	Signal peptide	No alpha-helix	Periplasmic	p>0,01	<0	-	>2,965	No beta-barrel	No lipoprotein	Vega 2005
ddlB	16128085	D-alanine:Dalanine ligase	Non-secretory protein	Non-secretory protein	No alpha-helix	cytoplasmic	p>0,01	<0	-	2,906	No beta-barrel	No lipoprotein	Zawadzke 1991
hflD	16129095	predicted lysogenization regulator	Non-secretory protein	Non-secretory protein	No alpha-helix	unknown	p>0,01	0,016	-	2,894	No beta-barrel	No lipoprotein	Kihara 2001
yaaI	16128007	predicted protein	Signal peptide	Signal peptide	No alpha-helix	unknown	p>0,01	<0	-	>2,965	Beta-barrel	No lipoprotein	
ybgO	90111164	predicted fimbrial-like adhesin protein	Signal peptide	Signal peptide	No alpha-helix	unknown	p>0,01	<0	-	>2,965	Beta-barrel	No lipoprotein	

yfcQ	16130269	predicted fimbrial-like adhesin protein	Signal peptide	Signal peptide	No alpha-helix	unknown	p>0,01	<0	-	2,948	No beta-barrel	No lipoprotein	
yadM	90111089	predicted fimbrial-like adhesin protein	Signal peptide	Signal peptide	No alpha-helix	unknown	p>0,01	<0	-	2,959	No beta-barrel	No lipoprotein	
ybfC	16128679	predicted protein	Signal peptide	Signal peptide	No alpha-helix	unknown	p>0,01	<0	-	>2,965	Beta-barrel	No lipoprotein	
yfaQ	16130163	predicted protein	Signal peptide	Signal peptide	No alpha-helix	unknown	p>0,01	<0	-	>2,965	Beta-barrel	No lipoprotein	
yfaT	90111404	predicted protein	Signal peptide	Signal peptide	No alpha-helix	unknown	p>0,01	<0	-	>2,965	Beta-barrel	No lipoprotein	
yijF	16131782	conserved protein	Signal peptide	Signal peptide	No alpha-helix	unknown	p>0,01	<0	-	>2,965	Beta-barrel	No lipoprotein	
ynfB	16129541	predicted protein	Signal peptide	Signal peptide	No alpha-helix	unknown	p>0,01	<0	-	>2,965	Beta-barrel	No lipoprotein	
ypeC	16130322	conserved protein	Signal peptide	Signal peptide	No alpha-helix	unknown	p>0,01	<0	-	>2,965	Beta-barrel	No lipoprotein	
yqhG	49176301	conserved protein	Signal peptide	Signal peptide	No alpha-helix	unknown	p>0,01	<0	-	>2,965	Beta-barrel	No lipoprotein	
yqiI	16130944	conserved protein	Signal peptide	Signal peptide	No alpha-helix	unknown	p>0,01	<0	-	>2,965	Beta-barrel	No lipoprotein	
flhE	16129830	conserved protein	Signal peptide	Signal peptide	No alpha-helix	unknown	p>0,01	<0	-	2,928	No beta-barrel	No lipoprotein	
yahO	16128314	predicted protein	Signal peptide	Signal peptide	No alpha-helix	unknown	p>0,01	<0	-	2,941	No beta-barrel	No lipoprotein	
yjjA	90111742	conserved protein	Signal peptide	Signal peptide	No alpha-helix	unknown	p>0,01	<0	-	2,944	No beta-barrel	No lipoprotein	
ydcA	16129378	predicted protein	Signal peptide	Signal peptide	No alpha-helix	unknown	p>0,01	<0	-	2,945	No beta-barrel	No lipoprotein	
yccT	16128931	conserved protein	Signal peptide	Signal peptide	No alpha-helix	unknown	p>0,01	<0	-	2,951	No beta-barrel	No lipoprotein	
yffQ	145698297	CPZ-55 prophage; predicted protein	Signal peptide	Signal peptide	No alpha-helix	unknown	p>0,01	<0	-	2,959	No beta-barrel	No lipoprotein	
yiiQ	16131758	conserved protein	Signal peptide	Signal peptide	No alpha-helix	unknown	p>0,01	<0	-	2,96	No beta-barrel	No lipoprotein	
ykgI	90111111	predicted protein	Signal peptide	Signal peptide	No alpha-helix	unknown	p>0,01	0,005	-	>2,965	No beta-barrel	No lipoprotein	
matC	16128277	predicted protein	Signal peptide	Signal peptide	No alpha-helix	unknown	p>0,01	0,018	-	>2,965	No beta-barrel	No lipoprotein	
bax	49176373	conserved protein	Signal peptide	Signal peptide	No alpha-helix	unknown	p>0,01	<0	-	>2,965	No beta-barrel	No lipoprotein	
fryB	16130319	predicted enzyme IIB component of PTS	Signal peptide	Signal peptide	No alpha-helix	unknown	p>0,01	<0	-	>2,965	No beta-barrel	No lipoprotein	
ybcS	16128538	DLP12 prophage; predicted lysozyme	Signal peptide	Signal peptide	No alpha-helix	unknown	p>0,01	<0	-	>2,965	No beta-barrel	No lipoprotein	
yiiX	16131775	predicted peptidoglycan peptidase	Signal peptide	Signal peptide	No alpha-helix	unknown	p>0,01	<0	-	>2,965	No beta-barrel	No lipoprotein	
yadK	16128129	predicted fimbrial-like adhesin protein	Signal peptide	Signal peptide	1 alpha-helix	unknown	p>0,01	<0	-	>2,965	No beta-barrel	No lipoprotein	
creA	16132214	conserved protein	Signal peptide	Signal peptide	No alpha-helix	unknown	p>0,01	<0	-	>2,965	No beta-barrel	No lipoprotein	
ycgJ	16129140	predicted protein	Signal peptide	Signal peptide	No alpha-helix	unknown	p>0,01	<0	-	>2,965	No beta-barrel	No lipoprotein	
yegJ	16130011	predicted protein	Signal peptide	Signal peptide	No alpha-helix	unknown	p>0,01	<0	-	>2,965	No beta-barrel	No lipoprotein	
yehE	16130050	predicted protein	Signal peptide	Signal peptide	No alpha-helix	unknown	p>0,01	<0	-	>2,965	No beta-barrel	No lipoprotein	
yfgI	16130431	conserved protein	Signal peptide	Signal peptide	No alpha-helix	unknown	p>0,01	<0	-	>2,965	No beta-barrel	No lipoprotein	
yfiR	16130524	predicted protein	Signal peptide	Signal peptide	No alpha-helix	unknown	p>0,01	<0	-	>2,965	No beta-barrel	No lipoprotein	
yibG	16131467	conserved protein	Signal peptide	Signal peptide	No alpha-helix	unknown	p>0,01	<0	-	>2,965	No beta-barrel	No lipoprotein	
yicS	94541128	predicted protein	Signal peptide	Signal peptide	No alpha-helix	unknown	p>0,01	<0	-	>2,965	No beta-barrel	No lipoprotein	

yjcO	15131904	conserved protein	Signal peptide	Signal peptide	No alpha-helix	unknown	p>0,01	<0	-	>2,965	No beta-barrel	No lipoprotein	
yjdP	49176458	conserved protein	Signal peptide	Signal peptide	No alpha-helix	unknown	p>0,01	<0	-	>2,965	No beta-barrel	No lipoprotein	
yjfY	16132021	predicted protein	Signal peptide	Signal peptide	No alpha-helix	unknown	p>0,01	<0	-	>2,965	No beta-barrel	No lipoprotein	
ymgD	90111226	predicted protein	Signal peptide	Signal peptide	No alpha-helix	unknown	p>0,01	<0	-	>2,965	No beta-barrel	No lipoprotein	
ynjH	16129714	predicted protein	Signal peptide	Signal peptide	No alpha-helix	unknown	p>0,01	<0	-	>2,965	No beta-barrel	No lipoprotein	
ycbB	16128892	predicted carboxypeptidase	Signal peptide	Signal peptide	No alpha-helix	unknown	0,0099	<0	-	>2,965	No beta-barrel	No lipoprotein	
csgC	16129006	predicted curli production protein	Signal peptide	Signal peptide	No alpha-helix	unknown	p>0,01	<0	-	2,943	No beta-barrel	No lipoprotein	
marB	16129491	predicted protein	Signal peptide	Signal peptide	No alpha-helix	unknown	p>0,01	<0	-	2,945	No beta-barrel	No lipoprotein	
ydgD	16129556	predicted peptidase	Signal peptide	Signal peptide	No alpha-helix	unknown	p>0,01	<0	-	>2,965	No beta-barrel	No lipoprotein	
yncJ	16129395	predicted protein	Signal peptide	Signal peptide	No alpha-helix	unknown	p>0,01	<0	-	>2,965	No beta-barrel	No lipoprotein	
ydfD	16129534	Qin prophage; predicted protein	Signal peptide	Non-secretory protein	No alpha-helix	unknown	p>0,01	<0	-	>2,965	No beta-barrel	No lipoprotein	
ydfW	145698265	Qin prophage; predicted protein	Signal peptide	Non-secretory protein	No alpha-helix	unknown	p>0,01	<0	-	>2,965	No beta-barrel	No lipoprotein	
sfmH	145698227	predicted fimbrial-like adhesin protein	Signal peptide	Signal peptide	No alpha-helix	unknown	p>0,01	<0	-	>2,965	Beta-barrel	No lipoprotein	
yebW	90111343	predicted protein	Signal peptide	Non-secretory protein	No alpha-helix	unknown	p>0,01	<0	-	>2,965	Beta-barrel	No lipoprotein	
yffR	16130374	CPZ-55 prophage; predicted protein	Signal peptide	Non-secretory protein	No alpha-helix	unknown	p>0,01	<0	-	>2,965	Beta-barrel	No lipoprotein	
yahM	90111114	predicted protein	Signal peptide	Non-secretory protein	No alpha-helix	unknown	p>0,01	0,015	-	>2,965	No beta-barrel	No lipoprotein	
yjfI	16132003	conserved protein	Signal peptide	Non-secretory protein	No alpha-helix	unknown	p>0,01	<0	-	>2,965	No beta-barrel	No lipoprotein	
yncH	16129414	predicted protein	Signal peptide	Non-secretory protein	No alpha-helix	unknown	p>0,01	<0	-	>2,965	No beta-barrel	No lipoprotein	
ymgI	145698243	hypothetical protein b4593	Signal peptide	Non-secretory protein	No alpha-helix	unknown	p>0,01	<0	-	>2,965	No beta-barrel	No lipoprotein	
hofP	90111582	predicted protein	Signal peptide	Non-secretory protein	No alpha-helix	unknown	p>0,01	<0	-	>2,965	No beta-barrel	No lipoprotein	
**False positives**													
cusB	16128557	copper/silver efflux system, membrane fusion protein	Signal peptide	Signal peptide	1 alpha-helix	unknown	p>0,01	<0	-	2,939	No beta-barrel	No lipoprotein	Silver 2003
yebF	145698278	predicted protein	Signal peptide	Signal peptide	No alpha-helix	unknown	p>0,01	<0	-	>2,965	No beta-barrel	No lipoprotein	EchoLocation
lolD	90111215	outer membrane-specific lipoprotein transporter subunit	Non-secretory protein	Non-secretory protein	No alpha-helix	cytoplasmic membrane	p>0,01	<0	-	2,937	No beta-barrel	No lipoprotein	Tokuda 2004
yacC	90111087	predicted protein	Signal peptide	Signal peptide	No alpha-helix	unknown	p>0,01	<0	-	>2,965	No beta-barrel	No lipoprotein	Hardie 1996

Table S2: *E. coli* K12 outer membrane proteins predicted in this study and their orthologues in *Escherichia coli* (UPEC) which were detected in outer membrane fractions in Walters and Mobley (2009). If the same gene/protein name could not be found, the closest BLAST hit was used. Details of the prediction results for the *E. coli* K12 proteins are shown in Table S1.

**Protein (*E. coli* K12)**	**gi (*E. coli* K12)**	**Protein (UPEC)**	**Pred. location (UPEC) according to Walters and Mobley (2009)**
Imp	16128048	Imp	Outer membrane
FhuA	16128143	FhuA	Outer membrane
YaeT	16128170	YaeT	Outer membrane
Tsx	16128396	Tsx	Outer membrane
OmpT	16128548	OmpT	Outer membrane
FepA	16128567	FepA	Outer membrane
Pal	16128716	Pal	Outer membrane
OmpF	16128896	OmpF	Outer membrane
OmpA	16128924	OmpA	Outer membrane
FimD	16132138	FocD	Outer membrane
FepA	16128567	IroN	Outer membrane
OmpN	16129338	C2348	Outer membrane
CirA	16130093	Hma	Outer membrane
OmpC	16130152	OmpC	Outer membrane
FadL	145698292	FadL	Outer membrane
CirA	16130093	C3610	Outer membrane
Flu	49176177	C3655	Outer membrane
TolC	90111528	TolC	Outer membrane
CirA	16130093	ChuA	Outer membrane
YiaD	49176370	YiaD	Outer membrane
BtuB	16131804	BtuB	Outer membrane
CirA	16130093	C5174	Outer membrane

NanC	90111728	C5389	Outer membrane
LolD*	90111215	LolD*	Inner membrane
RcsF	16128189	RcsF	Unknown
RlpA	16128616	RlpA	Unknown
RlpB	16128624	RlpB	Unknown
YbhC	16128740	YbhC	Unknown
YbjP	16128833	YbjP	Unknown
EmtA	90111231	MltE	Unknown
LolB	16129172	LolB	Unknown
SlyB	49176129	SlyB	Unknown
Lpp	16129633	Lpp	Unknown
YeaY	16129760	YeaY	Unknown
YfaZ	90111407	YfaZ	Unknown
VacJ	16130279	VacJ	Unknown
MltA	16130720	MltA	Unknown
Slp	90111603	Slp	Unknown
YifL	94541130	C4729	Unknown

* LolD is one of the four false positives in the prediction of *E. coli* K12 outer membrane proteins in this study.

Table S3: Predicted integral outer membrane proteins and outer membrane lipoproteins for *C. caviae* GPIC, *C. pneumoniae* AR39, *C. trachomatis* D/UW-3/CX, *C. muridarum* Nigg, and *P. amoebophila* UWE25.

*pCOMP cluster refers to the database pCOMP (www.microbial-ecology.net/pcomp)

LocusTag	Gi	Gene	Annotation	Location	COMP cluster*	TargetP	SignalP	TMHMM	Cpsortdb	Betawrap	MCMBB	BOMP	Pred-TMBB	HMBMM-B2R	LipoP
***Chlamydophila caviae* GPIC**															
CCA00014	29834130		hypothetical protein	integral	061	Signal peptide	Signal peptide	No alpha-helix	unknown	0.0012	<0	1	>2,965	Beta-barrel	No lipoprotein
CCA00017	29834133		hypothetical protein	integral	043	Non-secretory protein	Non-secretory protein	No alpha-helix	outer membrane	p>0,01	0,004	-	2,963	No beta-barrel	No lipoprotein
CCA00020	29834136		hypothetical protein	lipoprotein	072	Signal peptide	Signal peptide	1 alpha-helix	unknown	p>0,01	<0	-	>2,965	No beta-barrel	Outer membrane lipoprotein
CCA00040	29834155	sctC	type III secretion protein SctC	integral	020	Non-secretory protein	Non-secretory protein	1 alpha-helix	outer membrane	0.0043	<0	-	>2,965	No beta-barrel	No lipoprotein
CCA00047	29834162	ompA	major outer membrane protein, porin	integral	064	Signal peptide	Signal peptide	No alpha-helix	outer membrane	p>0,01	<0	2	2,963	Beta-barrel	No lipoprotein
CCA00062	29834177		hypothetical protein	integral	055	Non-secretory protein	Non-secretory protein	No alpha-helix	outer membrane	p>0,01	0,026	-	2,929	No beta-barrel	No lipoprotein
CCA00070	29834185		hypothetical protein	lipoprotein	060	Signal peptide	Signal peptide	No alpha-helix	cytoplasmic	p>0,01	<0	-	>2,965	No beta-barrel	Outer membrane lipoprotein
CCA00071	29834186		TPR domain protein	lipoprotein	040	Signal peptide	Signal peptide	No alpha-helix	unknown	p>0,01	<0	-	>2,965	No beta-barrel	Unknown membrane lipoprotein
CCA00078	29834193	mip	peptidyl-prolyl cis-trans isomerase Mip	integral (putative)	019	Signal peptide	Signal peptide	No alpha-helix	outer membrane	p>0,01	<0	-	>2,965	No beta-barrel	Cytoplasmic membrane lipoprotein
CCA00136	29834251		periplasmic amino acid-binding protein, putative	lipoprotein	002	Signal peptide	Signal peptide	1 alpha-helix	unknown	p>0,01	<0	-	>2,965	No beta-barrel	Outer membrane lipoprotein
CCA00146	29834261		hypothetical protein	integral (putative)	049	Signal peptide	Signal peptide	No alpha-helix	unknown	p>0,01	<0	-	>2,965	Beta-barrel	No lipoprotein
CCA00151	29834266		hypothetical protein	integral (putative)	070	Signal peptide	Signal peptide	No alpha-helix	unknown	p>0,01	<0	-	>2,965	Beta-barrel	No lipoprotein
CCA00159	29834274		hypothetical protein	lipoprotein	059	Signal peptide	Signal peptide	No alpha-helix	unknown	p>0,01	<0	-	>2,965	No beta-barrel	Outer membrane lipoprotein
CCA00170	29834284		hypothetical protein	integral	027	Non-secretory protein	Non-secretory protein	No alpha-helix	outer membrane	p>0,01	0,028	-	2,896	Beta-barrel	No lipoprotein
CCA00183	29834297		hypothetical protein	lipoprotein	078	Signal peptide	Signal peptide	No alpha-helix	unknown	p>0,01	<0	-	>2,965	No beta-barrel	Outer membrane lipoprotein
CCA00184	29834298	omcA	OmcA	integral (putative)	068	Signal peptide	Non-secretory protein	No alpha-helix	unknown	p>0,01	<0	-	>2,965	Beta-barrel	No lipoprotein
CCA00185	29834299	omcB	60 kDa outer membrane protein	integral (putative)	044	Signal peptide	Signal peptide	No alpha-helix	outer membrane	p>0,01	<0	-	>2,965	No beta-barrel	No lipoprotein
CCA00186	29834300	srp	Srp	integral (putative)	079	Non-secretory protein	Non-secretory protein	2 alpha-helices	outer membrane	p>0,01	<0	-	>2,965	No beta-barrel	No lipoprotein
CCA00205	29834319		polymorphic outer membrane protein B/C family protein/autotransporter, putative	integral	005	Signal peptide	Signal peptide	No alpha-helix	unknown	0.00013	0,015	-	2,945	Beta-barrel	No lipoprotein
CCA00206	29834320		polymorphic outer membrane protein A family protein/autotransporter, putative	integral	045	Signal peptide	Signal peptide	No alpha-helix	outer membrane	1.0e-07	<0	1	>2,965	Beta-barrel	No lipoprotein

CCA00262	29834376	artJ	amino acid ABC transporter, periplasmic amino acid-binding protein	lipoprotein	003	Signal peptide	Signal peptide	No alpha-helix	periplasmic	p>0,01	<0	-	>2,965	No beta-barrel	Outer membrane lipoprotein
CCA00270	29834384		hypothetical protein	integral (putative)	057	Non-secretory protein	Non-secretory protein	No alpha-helix	unknown	p>0,01	<0	-	>2,965	Beta-barrel	No lipoprotein
CCA00271	29834385		polymorphic outer membrane protein E/F family protein/autotransporter, putative	integral	037	Signal peptide	Signal peptide	No alpha-helix	unknown	3.0e-05	<0	-	>2,965	Beta-barrel	No lipoprotein
CCA00272	29834386		polymorphic outer membrane protein E/F family protein/autotransporter, putative	integral	037	Signal peptide	Non-secretory protein	No alpha-helix	outer membrane	1.3e-05	<0	-	>2,965	Beta-barrel	No lipoprotein
CCA00273	29834387		polymorphic outer membrane protein E/F family protein/autotransporter, putative	integral	037	Signal peptide	Non-secretory protein	No alpha-helix	outer membrane	0.00014	<0	1	>2,965	Beta-barrel	No lipoprotein
CCA00274	29834388		polymorphic outer membrane protein E/F family protein/autotransporter, putative	integral	046	Signal peptide	Signal peptide	No alpha-helix	outer membrane	1.9e-06	<0	-	>2,965	Beta-barrel	Outer membrane lipoprotein
CCA00275	29834390		polymorphic outer membrane protein E/F family protein, putative	integral	086	Signal peptide	Signal peptide	No alpha-helix	unknown	p>0,01	<0	-	2,959	Beta-barrel	No lipoprotein
CCA00277	29834392		polymorphic outer membrane protein H family protein	integral	007	Signal peptide	Signal peptide	No alpha-helix	unknown	0.00024	<0	1	>2,965	Beta-barrel	No lipoprotein
CCA00278	29834393		polymorphic outer membrane protein G family protein/autotransporter	integral	007	Signal peptide	Signal peptide	No alpha-helix	unknown	7.7e-05	<0	-	2,957	Beta-barrel	No lipoprotein
CCA00279	29834394		polymorphic outer membrane protein G family protein	integral	088	Signal peptide	Signal peptide	No alpha-helix	outer membrane	0.0012	<0	-	>2,965	Beta-barrel	No lipoprotein
CCA00280	29834395		polymorphic outer membrane protein G family protein/autotransporter, putative	integral	076	Signal peptide	Signal peptide	No alpha-helix	unknown	0.00019	<0	4	>2,965	Beta-barrel	No lipoprotein
CCA00281	29834396		polymorphic outer membrane protein G family protein/autotransporter, putative	integral	056	Signal peptide	Signal peptide	No alpha-helix	outer membrane	p>0,01	0,004	3	2,955	Beta-barrel	No lipoprotein
CCA00282	29834397		polymorphic outer membrane protein G family protein/autotransporter, putative	integral	007	Signal peptide	Signal peptide	No alpha-helix	extracellular	p>0,01	<0	1	2,945	Beta-barrel	No lipoprotein
CCA00283	29834398		polymorphic outer membrane protein G family protein/autotransporter	integral	042	Signal peptide	Signal peptide	No alpha-helix	unknown	0.0025	0,015	-	2,96	Beta-barrel	No lipoprotein
CCA00284	29834399		polymorphic outer membrane protein G family protein/autotransporter	integral	007	Signal peptide	Signal peptide	No alpha-helix	unknown	4.1e-08	0,009	2	2,944	Beta-barrel	No lipoprotein
CCA00292	29834405		hypothetical protein	integral	050	Signal peptide	Non-secretory protein	No alpha-helix	unknown	p>0,01	0,01	1	>2,965	Beta-barrel	No lipoprotein
CCA00389	29834500		hypothetical protein	lipoprotein (putative)	065	Non-secretory protein	Non-secretory protein	No alpha-helix	unknown	p>0,01	<0	-	2,951	No beta-barrel	No lipoprotein
CCA00390	29834501		hypothetical protein	lipoprotein (putative)	065	Signal peptide	Non-secretory protein	No alpha-helix	outer membrane	p>0,01	<0	-	2,87	No beta-barrel	No lipoprotein
CCA00395	29834505		hypothetical protein	lipoprotein	066	Signal peptide	Signal peptide	No alpha-helix	unknown	p>0,01	<0	-	>2,965	Beta-barrel	Outer membrane lipoprotein
CCA00417	29834527		hypothetical protein	integral	047	Non-secretory protein	Non-secretory protein	No alpha-helix	unknown	p>0,01	0,027	-	2,897	No beta-barrel	No lipoprotein
CCA00437	29834547		ABC transporter, periplasmic substrate-binding protein, putative	lipoprotein	010	Signal peptide	Signal peptide	No alpha-helix	unknown	p>0,01	<0	-	>2,965	No beta-barrel	Outer membrane lipoprotein
CCA00447	29834557	apbE	thiamine biosynthesis lipoprotein	lipoprotein	013	Signal peptide	Non-secretory protein	No alpha-helix	unknown	p>0,01	<0	-	>2,965	No beta-barrel	Outer membrane lipoprotein

CCA00451	29834561		hypothetical protein	integral	054	Non-secretory protein	Non-secretory protein	No alpha-helix	outer membrane	p>0,01	0,002	-	2,934	Beta-barrel	No lipoprotein
CCA00481	29834589		cationic outer membrane protein OmpH, putative	integral (putative)	030	Signal peptide	Signal peptide	No alpha-helix	unknown	p>0,01	<0	-	>2,965	No beta-barrel	No lipoprotein
CCA00482	29834590		outer membrane protein, putative	integral (putative)	009	Signal peptide	Signal peptide	No alpha-helix	outer membrane	p>0,01	<0	-	>2,965	Beta-barrel	No lipoprotein
CCA00523	29834630		hypothetical protein	integral	069	Non-secretory protein	Non-secretory protein	1 alpha-helix	unknown	0,01	0,034	-	>2,965	Beta-barrel	No lipoprotein
CCA00541	29834649		peptide ABC transporter, periplasmic peptide-binding protein, putative	lipoprotein	001	Signal peptide	Signal peptide	No alpha-helix	periplasmic	p>0,01	<0	-	>2,965	No beta-barrel	Outer membrane lipoprotein
CCA00575	29834682		hypothetical protein	lipoprotein	067	Signal peptide	Signal peptide	No alpha-helix	cytoplasmic membrane	p>0,01	<0	-	>2,965	No beta-barrel	Outer membrane lipoprotein
CCA00599	29834706		peptide ABC transporter, periplasmic binding protein	lipoprotein	036	Signal peptide	Signal peptide	1 alpha-helix	periplasmic	p>0,01	<0	-	>2,965	No beta-barrel	Outer membrane lipoprotein
CCA00600	29834707		peptide ABC transporter, periplasmic binding protein, putative	lipoprotein	015	Signal peptide	Signal peptide	No alpha-helix	unknown	p>0,01	<0	-	>2,965	No beta-barrel	Outer membrane lipoprotein
CCA00601	29834708	oppA	peptide ABC transporter, periplasmic binding protein	lipoprotein (putative)	001	Signal peptide	Signal peptide	1 alpha-helix	periplasmic	p>0,01	<0	-	>2,965	No beta-barrel	No lipoprotein
CCA00624	29834731		polymorphic outer membrane protein G family protein/autotransporter	integral	042	Signal peptide	Signal peptide	No alpha-helix	unknown	0.0014	0,015	-	2,954	Beta-barrel	No lipoprotein
CCA00668	29834775		hypothetical protein	integral (putative)	048	Signal peptide	Signal peptide	No alpha-helix	unknown	p>0,01	<0	-	>2,965	No beta-barrel	No lipoprotein
CCA00679	29834786		hypothetical protein	lipoprotein	062	Signal peptide	Signal peptide	1 alpha-helix	unknown	p>0,01	<0	-	>2,965	No beta-barrel	Outer membrane lipoprotein
CCA00729	29834835		hypothetical protein	integral	051	Signal peptide	Signal peptide	No alpha-helix	unknown	p>0,01	0,008	-	2,963	Beta-barrel	No lipoprotein
CCA00738	29834844		hypothetical protein	lipoprotein (putative)	082	Signal peptide	Signal peptide	No alpha-helix	unknown	p>0,01	<0	-	>2,965	Beta-barrel	No lipoprotein
CCA00755	29834861		hypothetical protein	integral	071	Non-secretory protein	Signal peptide	No alpha-helix	unknown	p>0,01	0,044	-	2,936	No beta-barrel	No lipoprotein
CCA00806	29834913		polymorphic outer membrane protein D family protein/autotransporter	integral	035	Non-secretory protein	Signal peptide	No alpha-helix	unknown	1.0e-06	0,005	-	2,95	Beta-barrel	No lipoprotein
CCA00812	29834919	ide	metalloprotease, insulinase family	lipoprotein	008	Signal peptide	Signal peptide	No alpha-helix	periplasmic	p>0,01	<0	-	>2,965	No beta-barrel	Outer membrane lipoprotein
CCA00892	29834998		hypothetical protein	lipoprotein	058	Signal peptide	Signal peptide	No alpha-helix	unknown	p>0,01	<0	-	>2,965	No beta-barrel	Outer membrane lipoprotein
CCA00913	29835018		major outer membrane protein, putative	integral	075	Signal peptide	Signal peptide	No alpha-helix	outer membrane	p>0,01	<0	4	2,92	No beta-barrel	No lipoprotein
CCA00935	29835040	sctJ	type III secretion protein SctJ	lipoprotein	028	Signal peptide	Signal peptide	1 alpha-helix	unknown	p>0,01	<0	-	2,952	No beta-barrel	Outer membrane lipoprotein
CCA00948	29835053	gspD	general secretion pathway protein D	lipoprotein (putative)	023	Signal peptide	Signal peptide	1 alpha-helix	outer membrane	p>0,01	<0	-	>2,965	No beta-barrel	No lipoprotein
CCA00975	29835080	tolB	translocation protein TolB precursor	lipoprotein (putative)	016	Signal peptide	Signal peptide	No alpha-helix	periplasmic	p>0,01	<0	-	>2,965	No beta-barrel	No lipoprotein
CCA00976	29835081		peptidoglycan-associated lipoprotein	lipoprotein	011	Signal peptide	Signal peptide	1 alpha-helix	outer membrane	p>0,01	<0	-	>2,965	No beta-barrel	Outer membrane lipoprotein
CCA01006	29835111		hypothetical protein	lipoprotein	063	Signal peptide	Signal peptide	1 alpha-helix	unknown	p>0,01	<0	-	>2,965	No beta-barrel	Outer membrane lipoprotein
***Chlamydophila pneumoniae* AR39**															
CP0017	7188956		hypothetical protein	integral	061	Signal peptide	Signal peptide	No alpha-helix	unknown	0.0026	<0	1	>2,965	Beta-barrel	No lipoprotein
CP0020	7188960		hypothetical protein	integral	043	Non-secretory protein	Non-secretory protein	No alpha-helix	unknown	p>0,01	0,017	-	2,932	No beta-barrel	No lipoprotein

CP0024	7188963		hypothetical protein	lipoprotein (putative)	072	Signal peptide	Signal peptide	1 alpha-helix	unknown	p>0,01	<0	-	>2,965	Beta-barrel	No lipoprotein
CP0044	7188983		type III secretion protein SctC	integral (putative)	020	Non-secretory protein	Non-secretory protein	No alpha-helix	outer membrane	p>0,01	<0	-	2,957	No beta-barrel	No lipoprotein
CP0051	7188990		major outer membrane protein, porin	integral	064	Signal peptide	Signal peptide	No alpha-helix	outer membrane	p>0,01	0,003	-	2,955	Beta-barrel	No lipoprotein
CP0070	7189006		hypothetical protein	integral (putative)	055	Non-secretory protein	Non-secretory protein	No alpha-helix	unknown	p>0,01	<0	-	2,937	No beta-barrel	No lipoprotein
CP0079	8163361		hypothetical protein	lipoprotein (putative)	040	Signal peptide	Signal peptide	No alpha-helix	unknown	p>0,01	<0	-	>2,965	No beta-barrel	Cytoplasmic membrane lipoprotein
CP0086	7189022		peptidyl-prolyl cis-trans isomerase Mip	integral (putative)	019	Signal peptide	Signal peptide	No alpha-helix	outer membrane	p>0,01	<0	-	2,931	No beta-barrel	Cytoplasmic membrane lipoprotein
CP0143	7189078		amino acid ABC transporter, periplasmic amino acid-binding protein	lipoprotein	002	Signal peptide	Signal peptide	1 alpha-helix	periplasmic	p>0,01	<0	-	>2,965	No beta-barrel	Outer membrane lipoprotein
CP0153	7189088		hypothetical protein	integral	049	Signal peptide	Signal peptide	No alpha-helix	outer membrane	0.0098	<0	-	>2,965	Beta-barrel	No lipoprotein
CP0158	7189093		hypothetical protein	integral	070	Non-secretory protein	Signal peptide	No alpha-helix	unknown	p>0,01	<0	1	>2,965	Beta-barrel	No lipoprotein
CP0166	7189100		hypothetical protein	lipoprotein	059	Non-secretory protein	Non-secretory protein	No alpha-helix	unknown	p>0,01	<0	-	>2,965	No beta-barrel	Outer membrane lipoprotein
CP0177	7189107		hypothetical protein	integral	027	Non-secretory protein	Non-secretory protein	No alpha-helix	outer membrane	p>0,01	0,037	-	2,914	Beta-barrel	No lipoprotein
CP0191	7189122		hypothetical protein	lipoprotein	078	Signal peptide	Signal peptide	No alpha-helix	unknown	p>0,01	<0	-	>2,965	No beta-barrel	Outer membrane lipoprotein
CP0193	7189124	omcA	OmcA	integral (putative)	068	Signal peptide	Non-secretory protein	No alpha-helix	unknown	p>0,01	<0	-	>2,965	No beta-barrel	No lipoprotein
CP0195	7189126	omcB	60 kDa outer membrane protein	lipoprotein	044	Signal peptide	Signal peptide	No alpha-helix	outer membrane	p>0,01	<0	-	>2,965	No beta-barrel	Unknown membrane lipoprotein
CP0196	7189127	srp	Srp	integral (putative)	079	Non-secretory protein	Non-secretory protein	3 alpha-helices	outer membrane	p>0,01	<0	-	2,948	No beta-barrel	No lipoprotein
CP0212	7189142		polymorphic membrane protein B/C family	integral	005	Signal peptide	Signal peptide	No alpha-helix	outer membrane	5.5e-08	0,028	-	2,921	Beta-barrel	No lipoprotein
CP0213	7189143		polymorphic membrane protein A family	integral	045	Signal peptide	Signal peptide	No alpha-helix	outer membrane	0.0014	0,005	4	>2,965	Beta-barrel	No lipoprotein
CP0272	7189197		amino acid ABC transporter, periplasmic amino acid-binding protein	lipoprotein	003	Signal peptide	Non-secretory protein	No alpha-helix	unknown	p>0,01	<0	-	>2,965	No beta-barrel	Outer membrane lipoprotein
CP0281	7189206		hypothetical protein	integral	057	Non-secretory protein	Non-secretory protein	No alpha-helix	outer membrane	p>0,01	0,009	-	2,99	No beta-barrel	No lipoprotein
CP0282	7189207		hypothetical protein	integral	057	Non-secretory protein	Non-secretory protein	No alpha-helix	unknown	p>0,01	<0	-	2,914	Beta-barrel	No lipoprotein
CP0283	7189208		polymorphic membrane protein E/F family	integral	037	Signal peptide	Non-secretory protein	No alpha-helix	unknown	0.00014	0,013	2	>2,965	Beta-barrel	No lipoprotein
CP0285	7189210		polymorphic membrane protein E/F family	integral	037	Signal peptide	Signal peptide	1 alpha-helix	unknown	9.2e-06	0,003	-	>2,965	Beta-barrel	No lipoprotein
CP0286	7189211		polymorphic membrane protein E/F family	integral	037	Signal peptide	Signal peptide	No alpha-helix	outer membrane	0.00012	0,005	1	>2,965	Beta-barrel	No lipoprotein
CP0298	7189224		polymorphic membrane protein H family	integral	007	Signal peptide	Signal peptide	No alpha-helix	unknown	1.5e-05	0,007	3	2,961	Beta-barrel	No lipoprotein
CP0299	7189225		polymorphic membrane protein G family	integral	007	Signal peptide	Signal peptide	No alpha-helix	outer membrane	3.7e-05	0,017	1	2,948	Beta-barrel	No lipoprotein

CP0301	7189228		polymorphic membrane protein G family	integral	045	Signal peptide	Signal peptide	No alpha-helix	unknown	0.00086	<0	-	>2,965	Beta-barrel	No lipoprotein
CP0302	7189229		polymorphic membrane protein G family	integral	052	Non-secretory protein	Signal peptide	No alpha-helix	outer membrane	4.1e-06	0,017	4	2,937	Beta-barrel	No lipoprotein
CP0303	7189230		polymorphic membrane protein G family	integral	007	Signal peptide	Signal peptide	No alpha-helix	unknown	8.7e-08	0,026	1	2,93	Beta-barrel	No lipoprotein
CP0306	7189233		polymorphic membrane protein G family	integral	052	Signal peptide	Signal peptide	1 alpha-helix	unknown	9.9e-07	0,032	1	2,915	Beta-barrel	No lipoprotein
CP0307	7189235		polymorphic membrane protein G family	integral	007	Signal peptide	Signal peptide	No alpha-helix	outer membrane	1.9e-05	0,032	1	2,912	Beta-barrel	No lipoprotein
CP0308	7189236		polymorphic membrane protein G family	integral	007	Signal peptide	Signal peptide	No alpha-helix	unknown	2.7e-06	0,034	1	2,937	Beta-barrel	No lipoprotein
CP0309	7189237		polymorphic membrane protein G family	integral	025	Signal peptide	Signal peptide	No alpha-helix	outer membrane	2.4e-07	0,015	1	2,91	Beta-barrel	No lipoprotein
CP0312	8163408		hypothetical protein	integral (putative)	050	Signal peptide	Non-secretory protein	No alpha-helix	unknown	p>0,01	<0	-	>2,965	Beta-barrel	No lipoprotein
CP0356	7189282		hypothetical protein	lipoprotein	066	Signal peptide	Signal peptide	No alpha-helix	unknown	p>0,01	<0	-	>2,965	Beta-barrel	Outer membrane lipoprotein
CP0411	7189335		ABC transporter, periplasmic substrate-binding protein, putative	lipoprotein (putative)	010	Signal peptide	Non-secretory protein	1 alpha-helix	unknown	p>0,01	<0	-	>2,965	No beta-barrel	No lipoprotein
CP0422	7189346		thiamine biosynthesis lipoprotein	lipoprotein	013	Signal peptide	Signal peptide	No alpha-helix	unknown	p>0,01	<0	-	>2,965	No beta-barrel	Outer membrane lipoprotein
CP0426	7189351		hypothetical protein	integral	054	Non-secretory protein	Non-secretory protein	No alpha-helix	outer membrane	p>0,01	<0	-	2,914	Beta-barrel	No lipoprotein
CP0457	7189380		cationic outer membrane protein OmpH, putative	integral (putative)	030	Signal peptide	Signal peptide	No alpha-helix	unknown	p>0,01	<0	-	>2,965	No beta-barrel	No lipoprotein
CP0458	8163436		outer membrane protein, putative	integral	009	Signal peptide	Non-secretory protein	No alpha-helix	outer membrane	0.0082	<0	-	>2,965	Beta-barrel	No lipoprotein
CP0506	7189423		hypothetical protein	integral	069	Non-secretory protein	Non-secretory protein	No alpha-helix	cytoplasmic membrane	0,0081	0,026	3	2,932	Beta-barrel	No lipoprotein
CP0569	7189481		peptide ABC transporter, periplasmic peptide-binding protein	lipoprotein	001	Signal peptide	Signal peptide	No alpha-helix	periplasmic	p>0,01	<0	-	>2,965	No beta-barrel	Outer membrane lipoprotein
CP0570	7189482		peptide ABC transporter, periplasmic peptide-binding protein, putative	lipoprotein	036	Signal peptide	Signal peptide	No alpha-helix	periplasmic	p>0,01	<0	-	>2,965	No beta-barrel	Outer membrane lipoprotein
CP0571	7189483		peptide ABC transporter, periplasmic peptide-binding protein, putative	lipoprotein	001	Signal peptide	Signal peptide	No alpha-helix	periplasmic	p>0,01	<0	-	>2,965	No beta-barrel	Outer membrane lipoprotein
CP0572	7189485		peptide ABC transporter, periplasmic peptide-binding protein, putative	lipoprotein	001	Signal peptide	Signal peptide	No alpha-helix	periplasmic	p>0,01	<0	-	>2,965	No beta-barrel	Outer membrane lipoprotein
CP0592	7189505		hypothetical protein	lipoprotein	067	Signal peptide	Signal peptide	No alpha-helix	unknown	p>0,01	<0	-	>2,965	Beta-barrel	Outer membrane lipoprotein
CP0593	8163463		MAC/perforin family protein	integral (putative)	080	Non-secretory protein	Non-secretory protein	No alpha-helix	unknown	p>0,01	<0	-	>2,965	No beta-barrel	No lipoprotein
CP0670	7189585		hypothetical protein	integral (putative)	048	Signal peptide	Signal peptide	No alpha-helix	unknown	p>0,01	<0	-	>2,965	Beta-barrel	No lipoprotein
CP0681	7189596		hypothetical protein	lipoprotein (putative)	062	Signal peptide	Non-secretory protein	1 alpha-helix	unknown	p>0,01	<0	-	>2,965	Beta-barrel	No lipoprotein
CP0756	7189671		hypothetical protein	integral (putative)	029	Signal peptide	Signal peptide	No alpha-helix	unknown	p>0,01	<0	-	>2,965	Beta-barrel	No lipoprotein
CP0761	7189674		polymorphic membrane protein G family	integral	052	Signal peptide	Signal peptide	No alpha-helix	outer membrane	0.00027	0,025	-	2,924	Beta-barrel	No lipoprotein
CP0770	7189685		polymorphic membrane protein G family	integral	052	Signal peptide	Signal peptide	No alpha-helix	outer membrane	0.00041	0,004	1	>2,965	Beta-barrel	No lipoprotein

CP0819	7189733		hypothetical protein	integral	051	Signal peptide	Signal peptide	No alpha-helix	unknown	p>0,01	0,02	1	2,937	Beta-barrel	No lipoprotein
CP0829	7189742		hypothetical protein	lipoprotein	082	Signal peptide	Signal peptide	1 alpha-helix	unknown	p>0,01	<0	-	>2,965	No beta-barrel	Outer membrane lipoprotein
CP0847	7189759		hypothetical protein	integral (putative)	071	Non-secretory protein	Non-secretory protein	No alpha-helix	unknown	p>0,01	0,056	-	>2,965	No beta-barrel	No lipoprotein
CP0897	7189810		polymorphic membrane protein D family	integral	035	Non-secretory protein	Signal peptide	1 alpha-helix	unknown	5.8e-06	<0	-	2,944	Beta-barrel	No lipoprotein
CP0903	7189816		metalloprotease, insulinase family	lipoprotein	008	Signal peptide	Signal peptide	No alpha-helix	periplasmic	p>0,01	<0	-	>2,965	No beta-barrel	Outer membrane lipoprotein
CP0994	7189907		hypothetical protein	lipoprotein	058	Signal peptide	Signal peptide	No alpha-helix	unknown	p>0,01	<0	-	>2,965	No beta-barrel	Outer membrane lipoprotein
CP0995	7189908		hypothetical protein	lipoprotein	073	Signal peptide	Signal peptide	No alpha-helix	unknown	p>0,01	<0	-	>2,965	No beta-barrel	Outer membrane lipoprotein
CP1015	7189929		major outer membrane protein, putative	integral	075	Signal peptide	Signal peptide	1 alpha-helix	outer membrane	p>0,01	0,02	1	2,944	Beta-barrel	No lipoprotein
CP1043	7189957		type III secretion protein SctJ	lipoprotein	028	Signal peptide	Signal peptide	2 alpha-helices	unknown	p>0,01	<0	-	2,943	No beta-barrel	Outer membrane lipoprotein
CP1056	7189969		general secretion pathway protein D	lipoprotein	023	Signal peptide	Signal peptide	1 alpha-helix	outer membrane	p>0,01	<0	-	>2,965	No beta-barrel	Outer membrane lipoprotein
CP1072	16752241		hypothetical protein CP1072	integral	089	Signal peptide	Non-secretory protein	No alpha-helix	unknown	0.0012	0,008	-	2,93	No beta-barrel	No lipoprotein
CP1074	16752243		hypothetical protein CP1074	integral	089	Signal peptide	Signal peptide	No alpha-helix	unknown	p>0,01	0,007	-	2,941	No beta-barrel	No lipoprotein
CP1075	16752244		hypothetical protein CP1075	integral	089	Non-secretory protein	Signal peptide	No alpha-helix	unknown	0.0069	0,01	-	2,928	Beta-barrel	No lipoprotein
CP1076	16752245		hypothetical protein CP1076	integral	089	Non-secretory protein	Non-secretory protein	No alpha-helix	outer membrane	p>0,01	0,012	1	>2,965	Beta-barrel	No lipoprotein
CP1077	16752246		hypothetical protein CP1077	integral	089	Signal peptide	Signal peptide	No alpha-helix	unknown	p>0,01	0,04	-	2,939	Beta-barrel	No lipoprotein
CP1090	7190004	tolB	translocation protein TolB precursor	lipoprotein (putative)	016	Signal peptide	Signal peptide	No alpha-helix	periplasmic	p>0,01	<0	-	>2,965	No beta-barrel	No lipoprotein
CP1091	7190005		peptidoglycan-associated lipoprotein	lipoprotein	011	Signal peptide	Signal peptide	No alpha-helix	outer membrane	p>0,01	<0	-	>2,965	No beta-barrel	Outer membrane lipoprotein
***Chlamydia trachomatis* D/UW-3/CX**															
CT007	3328395		hypothetical protein	integral	050	Signal peptide	Non-secretory protein	No alpha-helix	unknown	p>0,01	<0	4	>2,965	Beta-barrel	No lipoprotein
CT017	3328406		hypothetical protein	integral (putative)	048	Signal peptide	Non-secretory protein	No alpha-helix	unknown	p>0,01	<0	-	>2,965	No beta-barrel	No lipoprotein
CT050	3328442		hypothetical protein	integral (putative)	047	Non-secretory protein	Non-secretory protein	No alpha-helix	unknown	p>0,01	<0	-	2,952	No beta-barrel	No lipoprotein
CT051	3328443		hypothetical protein	integral	047	Non-secretory protein	Signal peptide	No alpha-helix	outer membrane	p>0,01	0,02	-	2,945	No beta-barrel	No lipoprotein
CT067	3328461	ytgA	Solute Protein Binding Family	lipoprotein	010	Signal peptide	Signal peptide	No alpha-helix	unknown	p>0,01	<0	-	>2,965	No beta-barrel	Outer membrane lipoprotein
CT077	3328472	yojL	hypothetical protein	lipoprotein	013	Signal peptide	Signal peptide	No alpha-helix	unknown	p>0,01	<0	-	>2,965	No beta-barrel	Outer membrane lipoprotein
CT082	3328477		hypothetical protein	integral	054	Non-secretory protein	Non-secretory protein	No alpha-helix	outer membrane	p>0,01	<0	-	2,92	Beta-barrel	No lipoprotein
CT105	3328502		hypothetical protein	lipoprotein	065	Non-secretory protein	Non-secretory protein	No alpha-helix	unknown	p>0,01	<0	-	>2,965	No beta-barrel	Unknown membrane
CT139	3328539	oppA_1	Oligopeptide Binding Protein	lipoprotein	036	Signal peptide	Non-secretory protein	No alpha-helix	periplasmic	p>0,01	<0	-	>2,965	No beta-barrel	Outer membrane lipoprotein
CT142	3328542		hypothetical protein	integral	069	Non-secretory protein	Non-secretory protein	No alpha-helix	unknown	p>0,01	0,012	-	2,957	Beta-barrel	No lipoprotein

CT153	3328555		hypothetical protein	integral (putative)	080	Non-secretory protein	Non-secretory protein	No alpha-helix	unknown	p>0,01	<0	-	>2,965	Beta-barrel	No lipoprotein
CT175	3328579	oppA_2	Oligopeptide binding protein permease	lipoprotein	001	Signal peptide	Signal peptide	No alpha-helix	periplasmic	p>0,01	<0	-	>2,965	No beta-barrel	Unknown membrane
CT198	3328604	oppA_3	Oligopeptide Binding Protein	lipoprotein	001	Signal peptide	Signal peptide	No alpha-helix	periplasmic	p>0,01	<0	-	>2,965	No beta-barrel	Outer membrane lipoprotein
CT241	3328651	yaeT	Omp85 Analog	integral	009	Signal peptide	Signal peptide	No alpha-helix	outer membrane	p>0,01	0,004	-	2,943	Beta-barrel	No lipoprotein
CT242	3328652		(OmpH-Like Outer Membrane Protein)	integral (putative)	030	Signal peptide	Signal peptide	No alpha-helix	unknown	p>0,01	<0	-	>2,965	No beta-barrel	No lipoprotein
CT253	3328664		hypothetical protein	lipoprotein (putative)	066	Signal peptide	Signal peptide	No alpha-helix	unknown	p>0,01	<0	-	>2,965	Beta-barrel	No lipoprotein
CT303	3328719		hypothetical protein	lipoprotein	062	Signal peptide	Signal peptide	No alpha-helix	unknown	p>0,01	<0	1	>2,965	Beta-barrel	Outer membrane lipoprotein
CT351	3328772		hypothetical protein	integral	029	Signal peptide	Non-secretory protein	No alpha-helix	unknown	0.0086	<0	-	>2,965	Beta-barrel	No lipoprotein
CT372	3328796		hypothetical protein	integral	051	Non-secretory protein	Non-secretory protein	No alpha-helix	unknown	0,0027	<0	-	>2,965	Beta-barrel	No lipoprotein
CT381	3328806	artJ	Arginine Binding Protein	lipoprotein	003	Signal peptide	Signal peptide	No alpha-helix	periplasmic	p>0,01	<0	-	>2,965	No beta-barrel	Outer membrane lipoprotein
CT412	3328840	pmpA	Putative outer membrane protein A	integral	045	Non-secretory protein	Non-secretory protein	1 alpha-helix	unknown	1.4e-07	<0	-	>2,965	Beta-barrel	No lipoprotein
CT413	3328841	pmpB	Putative outer membrane protein B	integral	005	Signal peptide	Non-secretory protein	No alpha-helix	outer membrane	2.3e-06	0,024	-	2,926	Beta-barrel	No lipoprotein
CT414	3328843	pmpC	Putative outer membrane protein C	integral	032	Signal peptide	Signal peptide	No alpha-helix	outer membrane	2.9e-06	0,017	-	2,936	Beta-barrel	No lipoprotein
CT442	3328873	crpA	15kDa Cysteine-Rich Protein	integral (putative)	079	Non-secretory protein	Non-secretory protein	2 alpha-helices	outer membrane	p>0,01	<0	-	2,934	No beta-barrel	No lipoprotein
CT443	3328874	omcB	60kDa Cysteine-Rich OMP	integral	044	Signal peptide	Signal peptide	No alpha-helix	outer membrane	0.00053	<0	-	>2,965	No beta-barrel	No lipoprotein
CT444	3328876	omcA	9kDa-Cysteine-Rich Lipoprotein	integral (putative)	068	Signal peptide	Signal peptide	No alpha-helix	unknown	p>0,01	<0	-	>2,965	Beta-barrel	No lipoprotein
CT444.1	3522897		hypothetical protein	lipoprotein	078	Signal peptide	Signal peptide	No alpha-helix	unknown	p>0,01	<0	-	>2,965	No beta-barrel	Outer membrane lipoprotein
CT456	3328889		hypothetical protein	integral	027	Non-secretory protein	Signal peptide	No alpha-helix	unknown	p>0,01	0,044	-	2,886	Beta-barrel	No lipoprotein
CT465	3328899		hypothetical protein	lipoprotein	059	Signal peptide	Signal peptide	No alpha-helix	unknown	p>0,01	<0	-	>2,965	No beta-barrel	Outer membrane lipoprotein
CT471	3328905		hypothetical protein	integral (putative)	070	Signal peptide	Signal peptide	1 alpha-helix	unknown	p>0,01	<0	-	>2,965	Beta-barrel	No lipoprotein
CT476	3328911		hypothetical protein	integral (putative)	049	Signal peptide	Signal peptide	No alpha-helix	unknown	p>0,01	<0	-	>2,965	Beta-barrel	No lipoprotein
CT486	3328922	fliY	Glutamine Binding Protein	lipoprotein (putative)	002	Signal peptide	Non-secretory protein	No alpha-helix	periplasmic	p>0,01	<0	-	2,924	No beta-barrel	No lipoprotein
CT541	3328979	mip	FKBP-type peptidyl-prolyl cis-trans isomerase	integral (putative)	019	Signal peptide	Signal peptide	No alpha-helix	outer membrane	p>0,01	<0	-	>2,965	No beta-barrel	Cytoplasmic membrane lipoprotein
CT548	3328987		hypothetical protein	lipoprotein	060	Signal peptide	Signal peptide	1 alpha-helix	unknown	p>0,01	<0	-	>2,965	Beta-barrel	Lipoprotein unknown location
CT559	3329000	yscJ	Yop proteins translocation lipoprotein J	lipoprotein (putative)	28	Signal peptide	Signal peptide	1 alpha-helix	unknown	p>0,01	<0	-	2,947	No beta-barrel	Cytoplasmic membrane lipoprotein
CT572	3329013	gspD	Gen. Secretion Protein D	lipoprotein (putative)	23	Signal peptide	Signal peptide	1 alpha-helix	outer membrane	p>0,01	<0	-	2,964	No beta-barrel	No lipoprotein

CT599	3329043	tolB	translocation protein TolB precursor	lipoprotein (putative)	16	Signal peptide	Non-secretory protein	No alpha-helix	periplasmic	p>0,01	<0	-	>2,965	No beta-barrel	No lipoprotein
CT600	3329044	pal	Peptidoglycan-Associated Lipoprotein	lipoprotein	11	Signal peptide	Signal peptide	No alpha-helix	outer membrane	p>0,01	<0	-	>2,965	No beta-barrel	Outer membrane lipoprotein
CT620	3329066		hypothetical protein	integral (putative)	43	Non-secretory protein	Non-secretory protein	No alpha-helix	unknown	p>0,01	0,009	-	>2,965	No beta-barrel	No lipoprotein
CT623	6578109		CHLPN 76kDa Homolog	integral	61	Signal peptide	Signal peptide	No alpha-helix	unknown	p>0,01	0,007	1	>2,965	Beta-barrel	No lipoprotein
CT654	3329104		hypothetical protein	lipoprotein (putative)	72	Signal peptide	Signal peptide	1 alpha-helix	unknown	p>0,01	<0	-	>2,965	Beta-barrel	No lipoprotein
CT674	3329125	yscC	probable Yop proteins translocation protein C/general secretion pathway protein	integral (putative)	20	Non-secretory protein	Signal peptide	No alpha-helix	outer membrane	p>0,01	<0	-	2,947	No beta-barrel	No lipoprotein
CT681	3329133	ompA	Major Outer Membrane Protein	integral	64	Signal peptide	Signal peptide	No alpha-helix	outer membrane	p>0,01	<0	2	2,955	Beta-barrel	No lipoprotein
CT694	3329148		hypothetical protein	integral (putative)	55	Non-secretory protein	Non-secretory protein	No alpha-helix	unknown	p>0,01	<0	-	>2,965	No beta-barrel	No lipoprotein
CT713	3329169	porB	Outer Membrane Protein Analog	integral	75	Signal peptide	Signal peptide	No alpha-helix	outer membrane	p>0,01	<0	1	>2,965	Beta-barrel	No lipoprotein
CT733	3329191		hypothetical protein	lipoprotein (putative)	73	Signal peptide	Signal peptide	No alpha-helix	unknown	p>0,01	<0	-	>2,965	No beta-barrel	No lipoprotein
CT734	3329192		hypothetical protein	lipoprotein	58	Signal peptide	Signal peptide	No alpha-helix	unknown	p>0,01	<0	-	2,928	No beta-barrel	Outer membrane lipoprotein
CT812	3329279	pmpD	Putative Outer Membrane Protein D	integral	35	Signal peptide	Signal peptide	No alpha-helix	outer membrane	1.7e-06	<0	2	2,919	Beta-barrel	No lipoprotein
CT849	3329321		hypothetical protein	integral	71	Non-secretory protein	Non-secretory protein	No alpha-helix	unknown	p>0,01	0,056	-	2,944	No beta-barrel	No lipoprotein
CT869	3329344	pmpE	Putative Outer Membrane Protein E	integral	37	Signal peptide	Signal peptide	No alpha-helix	outer membrane	3.0e-05	<0	1	>2,965	Beta-barrel	No lipoprotein
CT870	3329345	pmpF	Putative Outer Membrane Protein F	integral	46	Signal peptide	Signal peptide	No alpha-helix	outer membrane	8.1e-07	0,015	-	>2,965	Beta-barrel	No lipoprotein
CT871	3329346	pmpG	Putative Outer Membrane Protein G	integral	7	Signal peptide	Signal peptide	No alpha-helix	outer membrane	5.9e-06	0,016	1	2,952	Beta-barrel	No lipoprotein
CT872	3329347	pmpH	Putative Outer Membrane Protein H	integral	7	Signal peptide	Signal peptide	No alpha-helix	extracellular	5.5e-08	0,01	1	>2,965	Beta-barrel	No lipoprotein
CT874	3329350	pmpI	Putative Outer Membrane Protein I	integral	52	Signal peptide	Signal peptide	No alpha-helix	unknown	0.0024	<0	4	>2,965	Beta-barrel	No lipoprotein
CT875	3329351		hypothetical protein	integral (putative)	57	Non-secretory protein	Non-secretory protein	No alpha-helix	cytoplasmic	p>0,01	<0	-	>2,965	Beta-barrel	No lipoprotein
***Chlamydia muridarum* Nigg**															
TC0020	7190060		hypothetical protein	lipoprotein (putative)	63	Signal peptide	Signal peptide	No alpha-helix	unknown	p>0,01	<0	-	>2,965	No beta-barrel	No lipoprotein
TC0024	7190064		hypothetical protein	lipoprotein (putative)	72	Signal peptide	Signal peptide	1 alpha-helix	unknown	p>0,01	<0	-	>2,965	Beta-barrel	No lipoprotein
TC0045	7190084	sctC	type III secretion protein SctC	integral (putative)	20	Non-secretory protein	Non-secretory protein	No alpha-helix	outer membrane	p>0,01	<0	-	2,928	No beta-barrel	No lipoprotein
TC0052	7190091	ompA	major outer membrane protein, porin	integral	64	Signal peptide	Signal peptide	No alpha-helix	outer membrane	p>0,01	<0	2	>2,965	Beta-barrel	No lipoprotein
TC0066	7190101		hypothetical protein	integral (putative)	55	Non-secretory protein	Non-secretory protein	No alpha-helix	unknown	p>0,01	0,013	-	>2,965	No beta-barrel	No lipoprotein
TC0086	7190120		major outer membrane protein, putative	integral (putative)	75	Signal peptide	Signal peptide	No alpha-helix	outer membrane	p>0,01	<0	-	>2,965	Beta-barrel	No lipoprotein
TC0106	7190142		hypothetical protein	lipoprotein (putative)	73	Signal peptide	Signal peptide	No alpha-helix	unknown	p>0,01	<0	-	>2,965	No beta-barrel	No lipoprotein
TC0107	7190143		hypothetical protein	lipoprotein	58	Signal peptide	Signal peptide	No alpha-helix	unknown	p>0,01	<0	-	>2,965	No beta-barrel	Outer membrane lipoprotein

TC0190	7190228		metalloprotease, insulinase family	lipoprotein	8	Signal peptide	Signal peptide	No alpha-helix	periplasmic	p>0,01	<0	-	>2,965	No beta-barrel	Outer membrane lipoprotein
TC0197	7190234	pmpD	polymorphic membrane protein D family	integral	35	Non-secretory protein	Signal peptide	No alpha-helix	unknown	1.2e-07	<0	2	2,962	Beta-barrel	No lipoprotein
TC0237	7190276		hypothetical protein	integral	71	Non-secretory protein	Non-secretory protein	No alpha-helix	unknown	p>0,01	0,041	-	2,96	No beta-barrel	No lipoprotein
TC0261	7190301	pmpE/ F-1	polymorphic membrane protein E/F family	integral	37	Signal peptide	Non-secretory protein	No alpha-helix	outer membrane	0.00034	<0	-	>2,965	Beta-barrel	No lipoprotein
TC0262	7190302	pmpE/ F-2	polymorphic membrane protein E/F family	integral	46	Signal peptide	Signal peptide	No alpha-helix	outer membrane	3.5e-05	0,017	1	>2,965	Beta-barrel	No lipoprotein
TC0263	7190303	pmpG-1	polymorphic membrane protein G family	integral	7	Signal peptide	Signal peptide	No alpha-helix	outer membrane	5.6e-05	0,005	1	2,961	Beta-barrel	No lipoprotein
TC0264	7190304	pmpH	polymorphic membrane protein H family	integral	7	Signal peptide	Signal peptide	No alpha-helix	outer membrane	6.2e-07	0,002	1	>2,965	Beta-barrel	No lipoprotein
TC0267	7190308	pmpG-2	polymorphic membrane protein G family	integral	52	Signal peptide	Signal peptide	No alpha-helix	unknown	7.9e-05	<0	2	>2,965	Beta-barrel	No lipoprotein
TC0268	7190309		hypothetical protein	integral (putative)	57	Signal peptide	Non-secretory protein	No alpha-helix	unknown	p>0,01	<0	-	>2,965	Beta-barrel	No lipoprotein
TC0275	7190316		hypothetical protein	integral	50	Signal peptide	Non-secretory protein	No alpha-helix	unknown	p>0,01	<0	1	>2,965	Beta-barrel	No lipoprotein
TC0285	7190327		hypothetical protein	integral (putative)	48	Signal peptide	Signal peptide	No alpha-helix	unknown	p>0,01	<0	-	>2,965	No beta-barrel	No lipoprotein
TC0320	7190362		hypothetical protein	integral	47	Non-secretory protein	Non-secretory protein	No alpha-helix	unknown	p>0,01	0,005	-	>2,965	Beta-barrel	No lipoprotein
TC0321	7190363		hypothetical protein	integral	47	Non-secretory protein	Non-secretory protein	No alpha-helix	unknown	p>0,01	0,03	-	2,938	No beta-barrel	No lipoprotein
TC0338	7190380		ABC transporter, periplasmic substrate-binding protein, putative	lipoprotein	10	Signal peptide	Signal peptide	No alpha-helix	unknown	p>0,01	<0	-	>2,965	No beta-barrel	Outer membrane lipoprotein
TC0349	7190390		thiamine biosynthesis lipoprotein, putative	lipoprotein (putative)	13	Non-secretory protein	Non-secretory protein	No alpha-helix	unknown	p>0,01	<0	-	>2,965	No beta-barrel	No lipoprotein
TC0355	7190397		hypothetical protein	integral	54	Non-secretory protein	Non-secretory protein	No alpha-helix	outer membrane	p>0,01	0,01	-	2,926	Beta-barrel	No lipoprotein
TC0416	7190458		peptide ABC transporter, periplasmic peptide-binding protein, putative	lipoprotein	36	Signal peptide	Signal peptide	1 alpha-helix	periplasmic	p>0,01	<0	-	>2,965	No beta-barrel	Outer membrane lipoprotein
TC0419	7190461		hypothetical protein	integral	69	Non-secretory protein	Non-secretory protein	No alpha-helix	outer membrane	0.0023	0,023	-	2,944	Beta-barrel	No lipoprotein
TC0431	8163219		MAC/perforin family protein	integral (putative)	80	Non-secretory protein	Non-secretory protein	No alpha-helix	outer membrane	p>0,01	<0	-	>2,965	Beta-barrel	No lipoprotein
TC0437	7190480		adherence factor	integral	77	Non-secretory protein	Non-secretory protein	No alpha-helix	unknown	0.0070	<0	-	>2,965	No beta-barrel	No lipoprotein
TC0438	7190481		adherence factor	integral	77	Non-secretory protein	Non-secretory protein	No alpha-helix	unknown	0.00090	<0	-	>2,965	No beta-barrel	No lipoprotein
TC0439	7190483		adherence factor	integral	77	Non-secretory protein	Non-secretory protein	No alpha-helix	unknown	0.0092	<0	-	>2,965	No beta-barrel	No lipoprotein
TC0446	7190491		peptide ABC transporter, periplasmic peptide-binding protein	lipoprotein	1	Signal peptide	Signal peptide	No alpha-helix	unknown	p>0,01	<0	-	>2,965	No beta-barrel	Outer membrane lipoprotein
TC0449	7190494		hypothetical protein	lipoprotein	33	Signal peptide	Signal peptide	1 alpha-helix	unknown	p>0,01	<0	-	>2,965	No beta-barrel	Outer membrane lipoprotein
TC0471	7190512		peptide ABC transporter, periplasmic peptide-binding protein, putative	lipoprotein	1	Signal peptide	Signal peptide	No alpha-helix	periplasmic	p>0,01	<0	-	>2,965	No beta-barrel	Outer membrane lipoprotein
TC0512	7190552		outer membrane protein, putative	integral	9	Signal peptide	Signal peptide	No alpha-helix	outer membrane	p>0,01	0,005	-	2,94	Beta-barrel	No lipoprotein

TC0513	7190553		cationic outer membrane protein OmpH, putative	integral (putative)	30	Signal peptide	Signal peptide	No alpha-helix	unknown	p>0,01	<0	-	>2,965	No beta-barrel	No lipoprotein
TC0524	7190565		hypothetical protein	lipoprotein	66	Signal peptide	Signal peptide	No alpha-helix	unknown	p>0,01	<0	-	>2,965	Beta-barrel	Outer membrane lipoprotein
TC0577	7190616		hypothetical protein	lipoprotein (putative)	62	Signal peptide	Non-secretory protein	No alpha-helix	unknown	p>0,01	<0	-	>2,965	Beta-barrel	No lipoprotein
TC0630	7190668		hypothetical protein	integral	29	Signal peptide	Signal peptide	No alpha-helix	unknown	0.0099	<0	-	>2,965	Beta-barrel	No lipoprotein
TC0651	7190688		hypothetical protein	integral	51	Signal peptide	Non-secretory protein	No alpha-helix	unknown	0.0043	<0	-	>2,965	Beta-barrel	No lipoprotein
TC0660	7190695		amino acid ABC transporter, periplasmic amino acid-binding protein	lipoprotein	3	Signal peptide	Signal peptide	No alpha-helix	unknown	p>0,01	<0	-	>2,965	No beta-barrel	Outer membrane lipoprotein
TC0693	7190725	pmpA	polymorphic membrane protein A family	integral	45	Non-secretory protein	Non-secretory protein	No alpha-helix	outer membrane	3.7e-07	<0	-	>2,965	Beta-barrel	No lipoprotein
TC0694	7190726	pmpB/ C-1	polymorphic membrane protein B/C family	integral	5	Signal peptide	Signal peptide	No alpha-helix	unknown	6.2e-05	0,02	1	2,925	Beta-barrel	No lipoprotein
TC0695	7190727	pmpB/ C-2	polymorphic membrane protein B/C family	integral	32	Signal peptide	Signal peptide	No alpha-helix	outer membrane	4.0e-06	0,024	-	2,924	Beta-barrel	No lipoprotein
TC0726	7190755	srp	Srp	integral (putative)	79	Non-secretory protein	Non-secretory protein	2 alpha-helices	outer membrane	p>0,01	<0	-	2,918	No beta-barrel	No lipoprotein
TC0727	7190756	omcB	60 kDa outer membrane protein	integral	44	Signal peptide	Signal peptide	No alpha-helix	outer membrane	0.0018	<0	-	>2,965	No beta-barrel	No lipoprotein
TC0728	7190757	omcA	OmcA	integral (putative)	68	Signal peptide	Signal peptide	No alpha-helix	unknown	p>0,01	<0	-	>2,965	No beta-barrel	No lipoprotein
TC0729	7190758		hypothetical protein	lipoprotein	78	Signal peptide	Signal peptide	No alpha-helix	unknown	p>0,01	<0	-	>2,965	No beta-barrel	Outer membrane lipoprotein
TC0741	7190770		hypothetical protein	integral	27	Non-secretory protein	Signal peptide	No alpha-helix	unknown	p>0,01	0,024	-	2,918	No beta-barrel	No lipoprotein
TC0750	7190777		hypothetical protein	lipoprotein	59	Signal peptide	Signal peptide	No alpha-helix	unknown	p>0,01	<0	-	>2,965	No beta-barrel	Outer membrane lipoprotein
TC0756	7190781		hypothetical protein	integral (putative)	70	Signal peptide	Signal peptide	1 alpha-helix	unknown	p>0,01	<0	-	>2,965	Beta-barrel	No lipoprotein
TC0761	7190787		hypothetical protein	integral (putative)	49	Signal peptide	Signal peptide	No alpha-helix	unknown	p>0,01	<0	-	>2,965	Beta-barrel	No lipoprotein
TC0773	7190799		amino acid ABC transporter, periplasmic amino acid-binding protein	lipoprotein (putative)	2	Non-secretory protein	Signal peptide	1 alpha-helix	unknown	p>0,01	<0	-	2,945	No beta-barrel	No lipoprotein
TC0828	7190855	mip	peptidyl-prolyl cis-trans isomerase Mip	integral (putative)	19	Non-secretory protein	Signal peptide	1 alpha-helix	outer membrane	p>0,01	<0	-	>2,965	No beta-barrel	Cytoplasmic membrane lipoprotein
TC0836	7190864		hypothetical protein	lipoprotein (putative)	60	Signal peptide	Signal peptide	1 alpha-helix	unknown	p>0,01	<0	-	>2,965	Beta-barrel	No lipoprotein
TC0848	7190875	sctJ	type III secretion protein SctJ	lipoprotein (putative)	28	Signal peptide	Signal peptide	1 alpha-helix	cytoplasmic membrane	p>0,01	<0	-	2,94	No beta-barrel	Cytoplasmic membrane lipoprotein
TC0861	7190887	gspD	general secretion pathway protein D	lipoprotein (putative)	23	Signal peptide	Non-secretory protein	No alpha-helix	outer membrane	p>0,01	<0	-	>2,965	No beta-barrel	No lipoprotein
TC0888	7190916	tolB	translocation protein TolB precursor	lipoprotein	16	Signal peptide	Signal peptide	No alpha-helix	periplasmic	p>0,01	<0	-	>2,965	No beta-barrel	Outer membrane lipoprotein
TC0889	7190917		peptidoglycan associated lipoprotein, putative	lipoprotein (putative)	11	Signal peptide	Signal peptide	No alpha-helix	outer membrane	p>0,01	<0	-	>2,965	No beta-barrel	No lipoprotein
TC0910	7190938		hypothetical protein	integral	43	Non-secretory protein	Non-secretory protein	No alpha-helix	unknown	p>0,01	0,013	-	2,958	No beta-barrel	No lipoprotein
***Protochlamydia amoebophila* UWE25**															

pc0004	46399279		hypothetical protein	integral (putative)	61	Non-secretory protein	Signal peptide	No alpha-helix	unknown	p>0,01	<0	-	>2,965	Beta-barrel	No lipoprotein
pc0036	46445670		unknown protein	integral	89	Signal peptide	Non-secretory protein	No alpha-helix	outer membrane	p>0,01	0,033	1	>2,965	Beta-barrel	No lipoprotein
pc0074	46445708		unknown protein	integral	89	Signal peptide	Non-secretory protein	No alpha-helix	unknown	p>0,01	0,065	-	2,929	Beta-barrel	No lipoprotein
pc0081	46399356		hypothetical protein	lipoprotein	85	Signal peptide	Signal peptide	No alpha-helix	unknown	p>0,01	<0	-	>2,965	Beta-barrel	Outer membrane lipoprotein
pc0108	46399383	fliY	putative amino acid ABC transporter, periplasmic amino acid-binding protein	lipoprotein (putative)	2	Non-secretory protein	Signal peptide	1 alpha-helix	periplasmic	p>0,01	<0	-	>2,965	No beta-barrel	No lipoprotein
pc0186	46399461	wza	putative polysaccharide export protein wza	lipoprotein	24	Signal peptide	Signal peptide	No alpha-helix	unknown	p>0,01	<0	-	>2,965	No beta-barrel	Outer membrane lipoprotein
pc0205	46399480	yscJ	putative type III secretion protein SctJ	lipoprotein	28	Signal peptide	Non-secretory protein	2 alpha-helices	unknown	p>0,01	<0	-	2,948	No beta-barrel	Outer membrane lipoprotein
pc0256	46399531	ytgA	putative ABC transporter periplasmic substrate-binding protein ytgA	lipoprotein	10	Signal peptide	Signal peptide	No alpha-helix	cytoplasmic membrane	p>0,01	<0	-	>2,965	No beta-barrel	Outer membrane lipoprotein
pc0289	46399564	czcB	putative cation efflux system membrane protein B	integral (putative)	14	Signal peptide	Signal peptide	No alpha-helix	cytoplasmic	p>0,01	<0	-	>2,965	No beta-barrel	No lipoprotein
pc0290	46399565	czcC	putative cation efflux system membrane protein C	integral (putative)	22	Non-secretory protein	Non-secretory protein	No alpha-helix	outer membrane	p>0,01	0,002	-	>2,965	No beta-barrel	No lipoprotein
pc0291	46445925		unknown protein	lipoprotein	89	Non-secretory protein	Non-secretory protein	No alpha-helix	unknown	p>0,01	<0	-	>2,965	No beta-barrel	Outer membrane lipoprotein
pc0330	46399605		hypothetical protein	lipoprotein (putative)	59	Non-secretory protein	Signal peptide	No alpha-helix	unknown	p>0,01	<0	-	>2,965	Beta-barrel	No lipoprotein
pc0370	46399645		hypothetical protein	lipoprotein	49	Signal peptide	Signal peptide	No alpha-helix	unknown	p>0,01	<0	-	>2,965	Beta-barrel	Outer membrane lipoprotein
pc0383	46399658	mip	putative mip (macrophage infectivity potentiator, fkbp-type peptidyl-prolyl cis-trans isomerase)	integral (putative)	19	Signal peptide	Signal peptide	No alpha-helix	outer membrane	p>0,01	<0	-	>2,965	No beta-barrel	No lipoprotein
pc0390	46399665		hypothetical protein	lipoprotein (putative)	40	Signal peptide	Signal peptide	No alpha-helix	unknown	p>0,01	<0	-	>2,965	No beta-barrel	No lipoprotein
pc0391	46399666		hypothetical protein	lipoprotein	60	Signal peptide	Signal peptide	No alpha-helix	cytoplasmic	p>0,01	<0	-	>2,965	No beta-barrel	Outer membrane lipoprotein
pc0482	46399757		hypothetical protein	integral (putative)	48	Signal peptide	Signal peptide	No alpha-helix	unknown	p>0,01	<0	-	>2,965	No beta-barrel	No lipoprotein
pc0498	46446132		unknown protein	lipoprotein	89	Signal peptide	Signal peptide	No alpha-helix	cytoplasmic	p>0,01	<0	-	>2,965	No beta-barrel	Outer membrane lipoprotein
pc0552	46399827	oprK	putative outer membrane protein, componenet of multidrug efflux systems	integral (putative)	12	Signal peptide	Non-secretory protein	No alpha-helix	outer membrane	p>0,01	<0	-	2,93	No beta-barrel	No lipoprotein
pc0606	46446240	pal	strongly similar to peptidoglycan-associated lipoprotein precursor (pal)	lipoprotein	89	Signal peptide	Signal peptide	No alpha-helix	unknown	p>0,01	0,029	-	2,849	No beta-barrel	Outer membrane lipoprotein
pc0607	46399882		hypothetical protein	integral	29	Signal peptide	Signal peptide	No alpha-helix	unknown	0,0041	<0	-	>2,965	Beta-barrel	No lipoprotein
pc0616	46399891	omcB	putative 60 kDa cysteine-rich outer membrane protein	lipoprotein	44	Signal peptide	Signal peptide	No alpha-helix	outer membrane	p>0,01	<0	-	>2,965	No beta-barrel	Outer membrane lipoprotein
pc0634	46399909		hypothetical protein	integral (putative)	14	Signal peptide	Signal peptide	No alpha-helix	unknown	p>0,01	<0	-	>2,965	No beta-barrel	No lipoprotein
pc0635	46399910	tolC	putative outer membrane protein TolC	integral (putative)	4	Signal peptide	Signal peptide	No alpha-helix	unknown	p>0,01	<0	-	>2,965	Beta-barrel	No lipoprotein
pc0675	46399950		hypothetical protein	integral	50	Signal peptide	Signal peptide	No alpha-helix	outer membrane	p>0,01	0,022	-	2,941	Beta-barrel	No lipoprotein
pc0739	46400014	rhs	putative rhs core protein with extension	integral	31	Signal peptide	Signal peptide	No alpha-helix	outer membrane	0,0074	<0	3	>2,965	Beta-barrel	No lipoprotein

pc0777	46400052		hypothetical protein	lipoprotein	87	Signal peptide	Non-secretory protein	No alpha-helix	cytoplasmic	p>0,01	<0	-	>2,965	No beta-barrel	Outer membrane lipoprotein
pc0790	46446424		hypothetical protein	integral	89	Signal peptide	Signal peptide	1 alpha-helix	unknown	p>0,01	0,022	3	>2,965	No beta-barrel	No lipoprotein
pc0843	46400118		hypothetical protein	lipoprotein	84	Signal peptide	Signal peptide	No alpha-helix	unknown	p>0,01	<0	-	>2,965	Beta-barrel	Outer membrane lipoprotein
pc0874	46400149	potD	putative spermidine/putrescine-binding protein precursor, component of ABC transporter system	lipoprotein	6	Signal peptide	Signal peptide	No alpha-helix	periplasmic	p>0,01	<0	-	>2,965	No beta-barrel	Outer membrane lipoprotein
pc0895	46400170		putative outer membrane protein	lipoprotein	34	Signal peptide	Non-secretory protein	No alpha-helix	outer membrane	p>0,01	0,002	-	>2,965	No beta-barrel	Outer membrane lipoprotein
pc0921	46400196		hypothetical protein	integral (putative)	38	Non-secretory protein	Signal peptide	No alpha-helix	unknown	p>0,01	<0	-	>2,965	No beta-barrel	No lipoprotein
pc0938	46400213		hypothetical protein	integral	17	Signal peptide	Signal peptide	No alpha-helix	unknown	1,7e-005	0,044	1	2,891	No beta-barrel	No lipoprotein
pc0944	46400219		hypothetical protein	lipoprotein (putative)	58	Signal peptide	Non-secretory protein	1 alpha-helix	outer membrane	p>0,01	<0	-	>2,965	No beta-barrel	No lipoprotein
pc1030	46446664		unknown protein	integral	89	Signal peptide	Non-secretory protein	No alpha-helix	unknown	p>0,01	0,003	-	2,918	Beta-barrel	No lipoprotein
pc1071	46446705		hypothetical protein	integral	89	Signal peptide	Signal peptide	No alpha-helix	unknown	0,0031	0,015	-	>2,965	Beta-barrel	No lipoprotein
pc1077	46400352		hypothetical protein	integral	81	Signal peptide	Signal peptide	No alpha-helix	outer membrane	p>0,01	<0	4	2,958	Beta-barrel	No lipoprotein
pc1171	46400446	osmY	putative hyperosmotically inducible periplasmic protein	lipoprotein	41	Signal peptide	Signal peptide	No alpha-helix	cytoplasmic	p>0,01	<0	-	>2,965	No beta-barrel	Outer membrane lipoprotein
pc1219	46400494	oprM	putative outer membrane protein of AcrAB(MexAB)-OprM multidrug efflux pump	lipoprotein	18	Signal peptide	Non-secretory protein	No alpha-helix	outer membrane	p>0,01	<0	-	>2,965	No beta-barrel	Outer membrane lipoprotein
pc1295	46446929		unknown protein	integral	89	Signal peptide	Signal peptide	No alpha-helix	unknown	p>0,01	<0	2	>2,965	Beta-barrel	No lipoprotein
pc1304	46446938		unknown protein	integral	89	Signal peptide	Signal peptide	No alpha-helix	unknown	p>0,01	<0	1	>2,965	Beta-barrel	No lipoprotein
pc1353	46400628		conserved hypothetical protein (possible outer surface protein wsp)	integral	53	Non-secretory protein	Non-secretory protein	No alpha-helix	unknown	p>0,01	0,002	-	2,942	Beta-barrel	No lipoprotein
pc1380	46400655		hypothetical protein	integral	39	Non-secretory protein	Non-secretory protein	No alpha-helix	outer membrane	p>0,01	0,033	-	2,87	Beta-barrel	No lipoprotein
pc1463	46447097		unknown protein	integral	89	Signal peptide	Non-secretory protein	No alpha-helix	unknown	p>0,01	<0	-	2,917	Beta-barrel	No lipoprotein
pc1475	46400750	gspD	putative component D of type II secretion pathway	integral (putative)	20	Non-secretory protein	Non-secretory protein	No alpha-helix	outer membrane	p>0,01	<0	-	2,929	No beta-barrel	No lipoprotein
pc1489	46400764		hypothetical protein	integral	81	Signal peptide	Signal peptide	No alpha-helix	outer membrane	p>0,01	0,011	5	2,955	Beta-barrel	No lipoprotein
pc1502	46400777	oppA	probable oligo/dipeptide-binding protein oppA	lipoprotein (putative)	1	Signal peptide	Non-secretory protein	No alpha-helix	unknown	p>0,01	<0	-	>2,965	No beta-barrel	No lipoprotein
pc1659	46400934		hypothetical protein	lipoprotein	62	Signal peptide	Signal peptide	No alpha-helix	unknown	p>0,01	<0	-	>2,965	Beta-barrel	Outer membrane lipoprotein
pc1702	46400977	apbE	putative Thiamine biosynthesis lipoprotein apbE precursor	lipoprotein (putative)	13	Signal peptide	Signal peptide	No alpha-helix	unknown	p>0,01	<0	-	>2,965	No beta-barrel	No lipoprotein
pc1712	46400987		hypothetical protein	integral	83	Signal peptide	Signal peptide	No alpha-helix	unknown	p>0,01	<0	-	2,964	Beta-barrel	No lipoprotein
pc1726	46401001	omp85	putative outer membrane protein Omp85	integral (putative)	9	Non-secretory protein	Non-secretory protein	1 alpha-helix	outer membrane	p>0,01	<0	-	>2,965	Beta-barrel	No lipoprotein
pc1727	46401002		hypothetical protein	integral (putative)	30	Signal peptide	Signal peptide	1 alpha-helix	unknown	p>0,01	<0	-	>2,965	No beta-barrel	No lipoprotein
pc1749	46401024		hypothetical protein	integral (putative)	21	Signal peptide	Non-secretory protein	1 alpha-helix	outer membrane	p>0,01	<0	-	>2,965	Beta-barrel	No lipoprotein

pc1815	46401090	fbp	putative fibronectin/fibrinogen binding protein	integral (putative)	26	Signal peptide	Non-secretory protein	No alpha-helix	unknown	p>0,01	<0	-	>2,965	No beta-barrel	No lipoprotein
pc1850	46447484	tolB	translocation protein TolB	lipoprotein (putative)	16	Signal peptide	Signal peptide	No alpha-helix	periplasmic	p>0,01	<0	-	>2,965	No beta-barrel	No lipoprotein
pc1851	46401126	pal	probable peptidoglycan-associated lipoprotein precursor (pal)	lipoprotein	11	Signal peptide	Signal peptide	No alpha-helix	unknown	p>0,01	<0	-	>2,965	No beta-barrel	Outer membrane lipoprotein
pc1860	46401135		hypothetical protein	lipoprotein	81	Signal peptide	Signal peptide	No alpha-helix	outer membrane	p>0,01	<0	4	>2,965	Beta-barrel	Outer membrane lipoprotein
pc1862	46447496		unknown protein	integral	89	Signal peptide	Signal peptide	No alpha-helix	unknown	p>0,01	0,039	1	>2,965	Beta-barrel	No lipoprotein
pc1863	46447497		unknown protein	integral	89	Signal peptide	Signal peptide	No alpha-helix	unknown	p>0,01	0,002	-	2,924	Beta-barrel	No lipoprotein
pc1864	46447498		unknown protein	integral	89	Signal peptide	Signal peptide	No alpha-helix	outer membrane	p>0,01	0,048	-	2,939	Beta-barrel	No lipoprotein
pc1885	46401160		hypothetical protein	integral	74	Signal peptide	Signal peptide	No alpha-helix	cytoplasmic membrane	p>0,01	<0	1	2,944	Beta-barrel	No lipoprotein
pc1898	46401173	gspD	putative protein of the general secretion pathway	lipoprotein (putative)	23	Non-secretory protein	Non-secretory protein	No alpha-helix	outer membrane	p>0,01	<0	-	>2,965	No beta-barrel	No lipoprotein

Table S4: Predicted species-specific cell envelope components from *C. trachomatis* D/UW3/CX, *C. caviae* GPIC, *C. pneumoniae* AR39, and *P. amoebophila* UWE25.

LocusTag	Gi	Gene	Annotation	Location	TargetP	SignalP	TMHMM	Cpsortdb	Betawrap	MCMBB	BOMP	Pred-TMBB	HMBMM-B2R	LipoP
***Chlamydophila caviae* GPIC**														
CCA00140	29839908		hypothetical protein CCA00140	cell envelope	Signal peptide	Signal peptide	No alpha-helix	unknown	p>0,01	<0	-	>2,965	Beta-barrel	No lipoprotein
CCA00141	29839909		hypothetical protein CCA00141	cell envelope	Signal peptide	Signal peptide	No alpha-helix	unknown	p>0,01	<0	-	2,897	No beta-barrel	No lipoprotein
CCA00251	29840018		hypothetical protein CCA00251	cell envelope	Signal peptide	Non-secretory protein	No alpha-helix	unknown	p>0,01	0,01	-	>2,965	No beta-barrel	No lipoprotein
CCA00310	29840075		hypothetical protein CCA00310	cell envelope	Signal peptide	Signal peptide	No alpha-helix	unknown	p>0,01	<0	-	>2,965	No beta-barrel	No lipoprotein
CCA00332	29840096		hypothetical protein CCA00332	cell envelope	Signal peptide	Non-secretory protein	No alpha-helix	unknown	p>0,01	<0	-	>2,965	No beta-barrel	No lipoprotein
CCA00396	29840158		hypothetical protein CCA00396	cell envelope	Signal peptide	Non-secretory protein	No alpha-helix	unknown	p>0,01	<0	-	>2,965	No beta-barrel	No lipoprotein
CCA00405	29840167		hypothetical protein CCA00405	cell envelope	Signal peptide	Non-secretory protein	No alpha-helix	unknown	p>0,01	<0	-	>2,965	No beta-barrel	No lipoprotein
CCA00589	29840347		hypothetical protein CCA00589	cell envelope	Signal peptide	Non-secretory protein	No alpha-helix	unknown	p>0,01	<0	-	>2,965	No beta-barrel	No lipoprotein
CCA00704	29840462		hypothetical protein CCA00704	cell envelope	Signal peptide	Non-secretory protein	No alpha-helix	unknown	p>0,01	<0	-	>2,965	No beta-barrel	No lipoprotein
CCA00840	29840597		hypothetical protein CCA00840	cell envelope	Signal peptide	Non-secretory protein	No alpha-helix	unknown	p>0,01	<0	-	>2,965	No beta-barrel	No lipoprotein
***Chlamydophila pneumoniae* AR39**														
CP0083	16752376		hypothetical protein CP0083	cell envelope	Signal peptide	Non-secretory protein	No alpha-helix	unknown	p>0,01	<0	-	>2,965	No beta-barrel	No lipoprotein
CP0147	16753079		hypothetical protein CP0147	cell envelope	Signal peptide	Signal peptide	No alpha-helix	unknown	p>0,01	<0	-	2,837	No beta-barrel	No lipoprotein
CP0269	16752558		hypothetical protein CP0269	cell envelope	Signal peptide	Non-secretory protein	No alpha-helix	unknown	p>0,01	<0	-	>2,965	No beta-barrel	No lipoprotein
CP0347	16752632		hypothetical protein CP0347	cell envelope	Signal peptide	Non-secretory protein	No alpha-helix	unknown	p>0,01	<0	-	>2,965	No beta-barrel	No lipoprotein
CP0380	16752663		hypothetical protein CP0380	cell envelope	Non-secretory protein	Signal peptide	No alpha-helix	unknown	p>0,01	<0	-	>2,965	Beta-barrel	No lipoprotein
CP0443	16752724		hypothetical protein CP0443	cell envelope	Signal peptide	Non-secretory protein	No alpha-helix	unknown	p>0,01	0,004	-	>2,965	No beta-barrel	No lipoprotein
CP0562	16752836		hypothetical protein CP0562	cell envelope	Signal peptide	Non-secretory protein	No alpha-helix	unknown	p>0,01	<0	-	>2,965	No beta-barrel	No lipoprotein
CP0604	16752876		hypothetical protein CP0604	cell envelope	Signal peptide	Non-secretory protein	No alpha-helix	unknown	p>0,01	<0	-	>2,965	No beta-barrel	No lipoprotein
CP0758	16753026		hypothetical protein CP0758	cell envelope	Signal peptide	Signal peptide	No alpha-helix	unknown	p>0,01	<0	-	>2,965	No beta-barrel	No lipoprotein
CP0767	16753033		hypothetical protein CP0767	cell envelope	Signal peptide	Non-secretory protein	No alpha-helix	unknown	p>0,01	<0	-	>2,965	No beta-barrel	No lipoprotein
CP0988	16752158		hypothetical protein CP0988	cell envelope	Signal peptide	Non-secretory protein	No alpha-helix	unknown	p>0,01	<0	-	>2,965	No beta-barrel	No lipoprotein
CP1078	16752247		hypothetical protein CP1078	cell envelope	Signal peptide	Signal peptide	No alpha-helix	unknown	p>0,01	<0	-	>2,965	No beta-barrel	No lipoprotein
***Chlamydia trachomatis* D/UW-3/CX**														
CT638.1	15605370		hypothetical protein CT638.1	cell envelope	Signal peptide	Non-secretory protein	No alpha-helix	unknown	p>0,01	<0	-	>2,965	No beta-barrel	No lipoprotein
***Chlamydia muridarum* Nigg**														
TC0114	29337301		hypothetical protein TC0114	cell envelope	Signal peptide	Non-secretory protein	No alpha-helix	unknown	p>0,01	<0	-	>2,965	No beta-barrel	No lipoprotein
TC0165	15834785		hypothetical protein TC0165	cell envelope	Signal peptide	Non-secretory protein	No alpha-helix	unknown	p>0,01	<0	-	>2,965	Beta-barrel	No lipoprotein

TC0427	15835045		hypothetical protein TC0427	cell envelope	Signal peptide	Non-secretory protein	No alpha-helix	unknown	p>0,01	<0	-	>2,965	No beta-barrel	No lipoprotein
TC0766	15835380		hypothetical protein TC0766	cell envelope	Signal peptide	Signal peptide	No alpha-helix	unknown	p>0,01	<0	-	>2,965	No beta-barrel	No lipoprotein
***Protochlamydia amoebophila* UWE25**														
pc0068	46445702		hypothetical protein	cell envelope	Signal peptide	Non-secretory protein	No alpha-helix	unknown	p>0,01	<0	-	>2,965	No beta-barrel	No lipoprotein
pc0292	46445926		unknown protein	cell envelope	Signal peptide	Non-secretory protein	No alpha-helix	unknown	p>0,01	<0	-	>2,965	No beta-barrel	No lipoprotein
pc0348	46445982		conserved hypothetical protein	cell envelope	Signal peptide	Non-secretory protein	No alpha-helix	unknown	p>0,01	<0	-	>2,965	Beta-barrel	No lipoprotein
pc0359	46445993		unknown protein	cell envelope	Signal peptide	Non-secretory protein	No alpha-helix	unknown	p>0,01	<0	-	>2,965	Beta-barrel	No lipoprotein
pc0377	46446011		unknown protein	cell envelope	Signal peptide	Non-secretory protein	No alpha-helix	unknown	p>0,01	<0	-	>2,965	No beta-barrel	No lipoprotein
pc0405	46446039		hypothetical protein	cell envelope	Signal peptide	Non-secretory protein	No alpha-helix	unknown	p>0,01	<0	-	>2,965	No beta-barrel	No lipoprotein
pc0411	46446045		unknown protein	cell envelope	Signal peptide	Non-secretory protein	1 alpha-helix	unknown	p>0,01	<0	-	>2,965	No beta-barrel	No lipoprotein
pc0510	46446144		unknown protein	cell envelope	Non-secretory protein	Signal peptide	No alpha-helix	unknown	p>0,01	<0	-	>2,965	No beta-barrel	No lipoprotein
pc0522	46446156		unknown protein	cell envelope	Signal peptide	Signal peptide	No alpha-helix	unknown	p>0,01	<0	-	>2,965	No beta-barrel	No lipoprotein
pc0535	46446169		unknown protein	cell envelope	Signal peptide	Non-secretory protein	No alpha-helix	unknown	p>0,01	<0	-	>2,965	No beta-barrel	No lipoprotein
pc0554	46446188		conserved hypothetical protein	cell envelope	Non-secretory protein	Non-secretory protein	No alpha-helix	outer membrane	p>0,01	<0	-	>2,965	No beta-barrel	No lipoprotein
pc0574	46446208		unknown protein	cell envelope	Signal peptide	Signal peptide	No alpha-helix	unknown	p>0,01	<0	-	>2,965	Beta-barrel	No lipoprotein
pc0617	46446251	omcA	similar to 9 kDa cysteine-rich outer membrane protein	cell envelope	Non-secretory protein	Non-secretory protein	No alpha-helix	unknown	p>0,01	<0	-	>2,965	Beta-barrel	No lipoprotein
pc0629	46446263		unknown protein	cell envelope	Signal peptide	Non-secretory protein	No alpha-helix	unknown	p>0,01	<0	-	>2,965	Beta-barrel	No lipoprotein
pc0661	46446295		unknown protein	cell envelope	Signal peptide	Non-secretory protein	No alpha-helix	unknown	p>0,01	<0	-	>2,965	No beta-barrel	No lipoprotein
pc0694	46446328		unknown protein	cell envelope	Signal peptide	Signal peptide	No alpha-helix	unknown	p>0,01	<0	-	>2,965	Beta-barrel	No lipoprotein
pc0748	46446382		unknown protein	cell envelope	Non-secretory protein	Signal peptide	No alpha-helix	unknown	p>0,01	<0	-	>2,965	No beta-barrel	No lipoprotein
pc0770	46446404		unknown protein	cell envelope	Signal peptide	Non-secretory protein	No alpha-helix	unknown	p>0,01	<0	-	>2,965	Beta-barrel	No lipoprotein
pc0832	46446466		unknown protein	cell envelope	Signal peptide	Non-secretory protein	No alpha-helix	unknown	p>0,01	<0	-	>2,965	Beta-barrel	No lipoprotein
pc0876	46446510		unknown protein	cell envelope	Signal peptide	Non-secretory protein	No alpha-helix	unknown	p>0,01	<0	-	>2,965	No beta-barrel	No lipoprotein
pc0899	46446533		unknown protein	cell envelope	Non-secretory protein	Non-secretory protein	No alpha-helix	unknown	0,0072	<0	-	>2,965	No beta-barrel	No lipoprotein
pc0943	46446577	blc	similar to outer membrane lipoprotein	cell envelope	Signal peptide	Non-secretory protein	No alpha-helix	unknown	p>0,01	<0	-	>2,965	Beta-barrel	No lipoprotein
pc0991	46446625		unknown protein	cell envelope	Signal peptide	Non-secretory protein	No alpha-helix	unknown	p>0,01	<0	-	>2,965	Beta-barrel	No lipoprotein
pc1013	46446647		unknown protein	cell envelope	Signal peptide	Non-secretory protein	No alpha-helix	unknown	p>0,01	0,021	-	>2,965	No beta-barrel	No lipoprotein
pc1020	46446654		unknown protein	cell envelope	Signal peptide	Non-secretory protein	No alpha-helix	unknown	p>0,01	<0	-	>2,965	Beta-barrel	No lipoprotein
pc1024	46446658		unknown protein	cell envelope	Signal peptide	Non-secretory protein	No alpha-helix	unknown	p>0,01	<0	-	>2,965	Beta-barrel	No lipoprotein
pc1027	46446661		unknown protein	cell envelope	Signal peptide	Non-secretory protein	No alpha-helix	unknown	p>0,01	<0	-	>2,965	No beta-barrel	No lipoprotein

pc1037	46446671		unknown protein	cell envelope	Signal peptide	Non-secretory protein	No alpha-helix	unknown	p>0,01	<0	-	>2,965	No beta-barrel	No lipoprotein
pc1065	46446699		conserved hypothetical protein	cell envelope	Non-secretory protein	Non-secretory protein	No alpha-helix	outer membrane	p>0,01	<0	-	>2,965	No beta-barrel	No lipoprotein
pc1170	46446804		unknown protein	cell envelope	Signal peptide	Non-secretory protein	No alpha-helix	unknown	p>0,01	<0	-	>2,965	Beta-barrel	No lipoprotein
pc1185	46446819		unknown protein	cell envelope	Signal peptide	Signal peptide	No alpha-helix	unknown	p>0,01	<0	-	>2,965	No beta-barrel	No lipoprotein
pc1263	46446897		hypothetical protein	cell envelope	Signal peptide	Non-secretory protein	No alpha-helix	unknown	p>0,01	<0	-	>2,965	No beta-barrel	No lipoprotein
pc1294	46446928		unknown protein	cell envelope	Signal peptide	Non-secretory protein	No alpha-helix	unknown	p>0,01	<0	-	>2,965	Beta-barrel	No lipoprotein
pc1326	46446960		unknown protein	cell envelope	Signal peptide	Non-secretory protein	No alpha-helix	unknown	p>0,01	<0	-	>2,965	No beta-barrel	No lipoprotein
pc1350	46446984		unknown protein	cell envelope	Signal peptide	Non-secretory protein	1 alpha-helix	unknown	p>0,01	<0	-	>2,965	Beta-barrel	No lipoprotein
pc1366	46447000		unknown protein	cell envelope	Signal peptide	Non-secretory protein	No alpha-helix	unknown	p>0,01	<0	-	>2,965	No beta-barrel	No lipoprotein
pc1427	46447061		conserved hypothetical protein	cell envelope	Signal peptide	Non-secretory protein	No alpha-helix	unknown	p>0,01	0,003	-	>2,965	No beta-barrel	No lipoprotein
pc1437	46447071	traN	similar to conjugative transfer protein traN precursor	cell envelope	Non-secretory protein	Non-secretory protein	No alpha-helix	outer membrane	p>0,01	<0	-	>2,965	No beta-barrel	No lipoprotein
pc1465	46447099		hypothetical protein	cell envelope	Signal peptide	Non-secretory protein	No alpha-helix	unknown	p>0,01	<0	-	>2,965	No beta-barrel	No lipoprotein
pc1494	46447128		unknown protein	cell envelope	Signal peptide	Non-secretory protein	No alpha-helix	unknown	p>0,01	<0	-	>2,965	Beta-barrel	No lipoprotein
pc1500	46447134		unknown protein	cell envelope	Signal peptide	Non-secretory protein	No alpha-helix	unknown	p>0,01	<0	-	>2,965	Beta-barrel	No lipoprotein
pc1509	46447143		hypothetical protein	cell envelope	Signal peptide	Non-secretory protein	No alpha-helix	unknown	p>0,01	<0	-	>2,965	No beta-barrel	No lipoprotein
pc1554	46447188		conserved hypothetical protein	cell envelope	Signal peptide	Non-secretory protein	No alpha-helix	unknown	p>0,01	<0	-	>2,965	No beta-barrel	No lipoprotein
pc1555	46447189		unknown protein	cell envelope	Signal peptide	Signal peptide	No alpha-helix	unknown	p>0,01	<0	-	>2,965	Beta-barrel	No lipoprotein
pc1560	46447194		unknown protein	cell envelope	Signal peptide	Signal peptide	No alpha-helix	unknown	p>0,01	<0	-	>2,965	No beta-barrel	No lipoprotein
pc1582	46447216		unknown protein	cell envelope	Signal peptide	Non-secretory protein	No alpha-helix	unknown	p>0,01	<0	-	>2,965	Beta-barrel	No lipoprotein
pc1653	46447287		conserved hypothetical protein	cell envelope	Signal peptide	Non-secretory protein	No alpha-helix	unknown	p>0,01	<0	-	>2,965	No beta-barrel	No lipoprotein
pc1687	46447321		unknown protein	cell envelope	Signal peptide	Signal peptide	No alpha-helix	unknown	p>0,01	<0	-	>2,965	No beta-barrel	No lipoprotein
pc1716	46447350		unknown protein	cell envelope	Non-secretory protein	Non-secretory protein	No alpha-helix	outer membrane	p>0,01	<0	-	>2,965	Beta-barrel	No lipoprotein
pc1817	46447451		unknown protein	cell envelope	Signal peptide	Non-secretory protein	No alpha-helix	unknown	p>0,01	<0	-	>2,965	No beta-barrel	No lipoprotein
pc1821	46447455		unknown protein	cell envelope	Signal peptide	Signal peptide	No alpha-helix	unknown	p>0,01	<0	-	>2,965	No beta-barrel	No lipoprotein
pc1908	46447542		unknown protein	cell envelope	Signal peptide	Non-secretory protein	No alpha-helix	unknown	p>0,01	<0	-	>2,965	No beta-barrel	No lipoprotein
pc1919	46447553		conserved hypothetical protein	cell envelope	Signal peptide	Non-secretory protein	No alpha-helix	unknown	p>0,01	<0	-	>2,965	No beta-barrel	No lipoprotein
pc1927	46447561		conserved hypothetical protein	cell envelope	Signal peptide	Non-secretory protein	No alpha-helix	unknown	p>0,01	<0	-	>2,965	No beta-barrel	No lipoprotein
pc1948	46447582		unknown protein	cell envelope	Signal peptide	Non-secretory protein	No alpha-helix	unknown	p>0,01	<0	-	>2,965	No beta-barrel	No lipoprotein
pc1994	46447628		unknown protein	cell envelope	Signal peptide	Non-secretory protein	1 alpha-helix	unknown	p>0,01	<0	-	>2,965	No beta-barrel	No lipoprotein
pc2018	46447652		unknown protein	cell envelope	Signal peptide	Non-secretory protein	No alpha-helix	unknown	p>0,01	<0	-	>2,965	No beta-barrel	No lipoprotein

pc2026	46447660		unknown protein	cell envelope	Signal peptide	Non-secretory protein	No alpha-helix	unknown	p>0,01	<0	-	>2,965	No beta-barrel	No lipoprotein

Table S5: Predicted cell envelope components from *C. trachomatis* D/UW3/CX, *C. caviae* GPIC, *C. pneumoniae* AR39, and *P. amoebophila* UWE25 in clusters not assigned to the outer membrane.

LocusTag	Gi	Genes	Annotation	Location	TargetP	SignalP	TMHMM	Cpsortdb	Betawrap p-values	MCMBB	BOMP	Pred-TMBB	HMBMM-B2R	LipoP
***Chlamydophila caviae* GPIC**														
CCA00067	29839835		D-alanyl-D-alanine carboxypeptidase	cell envelope	Non-secretory protein	Signal peptide	1 alpha-helix	cytoplasmic membrane	p>0,01	<0	-	>2,965	No beta-barrel	No lipoprotein
CCA00131	29839899		hypothetical protein CCA00131	cell envelope	Signal peptide	Non-secretory protein	No alpha-helix	unknown	p>0,01	<0	-	>2,965	No beta-barrel	No lipoprotein
CCA00148	29839916		LysM domain protein	cell envelope	Signal peptide	Non-secretory protein	1 alpha-helix	cytoplasmic	p>0,01	<0	-	>2,965	No beta-barrel	No lipoprotein
CCA00155	29839923		sigma-54 dependent response regulator	cell envelope	Non-secretory protein	Non-secretory protein	No alpha-helix	cytoplasmic	p>0,01	<0	-	>2,965	No beta-barrel	No lipoprotein
CCA00163	29839930		hypothetical protein CCA00163	cell envelope	Signal peptide	Signal peptide	No alpha-helix	unknown	p>0,01	<0	-	>2,965	No beta-barrel	No lipoprotein
CCA00189	29839956		hypothetical protein CCA00189	cell envelope	Signal peptide	Signal peptide	No alpha-helix	unknown	p>0,01	<0	-	>2,965	No beta-barrel	No lipoprotein
CCA00377	29840140		N-acetylmuramoyl-L-alanine amidase, family 3	cell envelope	Non-secretory protein	Signal peptide	1 alpha-helix	unknown	p>0,01	<0	-	>2,965	No beta-barrel	No lipoprotein
CCA00382	29840145		hypothetical protein CCA00382	cell envelope	Signal peptide	Non-secretory protein	No alpha-helix	unknown	p>0,01	<0	-	>2,965	No beta-barrel	No lipoprotein
CCA00407	29840169		hypothetical protein CCA00407	cell envelope	Signal peptide	Signal peptide	No alpha-helix	unknown	p>0,01	<0	-	>2,965	No beta-barrel	No lipoprotein
CCA00443	29840205		hypothetical protein CCA00443	cell envelope	Signal peptide	Signal peptide	No alpha-helix	unknown	p>0,01	<0	-	>2,965	No beta-barrel	No lipoprotein
CCA00513	29840273		hypothetical protein CCA00513	cell envelope	Non-secretory protein	Non-secretory protein	2 alpha-helices	outer membrane	p>0,01	<0	-	2,877	No beta-barrel	No lipoprotein
CCA00578	29840336		hypothetical protein CCA00578	cell envelope	Signal peptide	Signal peptide	No alpha-helix	unknown	p>0,01	<0	-	>2,965	Beta-barrel	No lipoprotein
CCA00595	29840353		hypothetical protein CCA00595	cell envelope	Signal peptide	Signal peptide	No alpha-helix	unknown	p>0,01	<0	-	>2,965	Beta-barrel	No lipoprotein
CCA00623	29840381		hypothetical protein CCA00623	cell envelope	Signal peptide	Non-secretory protein	No alpha-helix	unknown	p>0,01	<0	-	>2,965	No beta-barrel	No lipoprotein
CCA00640	29840398		conserved hypothetical protein TIGR00486	cell envelope	Signal peptide	Non-secretory protein	No alpha-helix	unknown	p>0,01	<0	-	>2,965	No beta-barrel	No lipoprotein
CCA00644	29840402		hypothetical protein CCA00644	cell envelope	Signal peptide	Non-secretory protein	No alpha-helix	unknown	p>0,01	<0	-	>2,965	No beta-barrel	No lipoprotein
CCA00687	29840445		hypothetical protein CCA00687	cell envelope	Signal peptide	Signal peptide	No alpha-helix	unknown	p>0,01	<0	-	>2,965	No beta-barrel	No lipoprotein
CCA00824	29840581		hypothetical protein CCA00824	cell envelope	Signal peptide	Signal peptide	No alpha-helix	unknown	p>0,01	<0	-	>2,965	No beta-barrel	No lipoprotein
CCA00825	29840582		hypothetical protein CCA00825	cell envelope	Signal peptide	Signal peptide	No alpha-helix	unknown	p>0,01	<0	-	>2,965	No beta-barrel	No lipoprotein
CCA00826	29840583		hypothetical protein CCA00826	cell envelope	Signal peptide	Signal peptide	No alpha-helix	unknown	p>0,01	<0	-	>2,965	No beta-barrel	No lipoprotein
CCA00827	29840584		hypothetical protein CCA00827	cell envelope	Signal peptide	Signal peptide	No alpha-helix	unknown	p>0,01	<0	-	2,91	No beta-barrel	No lipoprotein
CCA00842	29840599		hypothetical protein CCA00842	cell envelope	Signal peptide	Signal peptide	No alpha-helix	unknown	p>0,01	<0	-	>2,965	No beta-barrel	No lipoprotein
CCA00847	29840604		acylglycerophosphoethanolamine acyltransferase	cell envelope	Non-secretory protein	Non-secretory protein	1 alpha-helix	cytoplasmic membrane	p>0,01	<0	-	>2,965	No beta-barrel	No lipoprotein
CCA00863	29840620	murC/ddlA	UDP-N-acetylmuramate--alanine ligase/D-alanine--D-alanine ligase	cell envelope	Non-secretory protein	Signal peptide	1 alpha-helix	cytoplasmic	p>0,01	<0	-	>2,965	No beta-barrel	No lipoprotein
CCA00866	29840623		LysM domain protein	cell envelope	Signal peptide	Non-secretory protein	1 alpha-helix	unknown	p>0,01	<0	-	>2,965	No beta-barrel	No lipoprotein
CCA00942	29840697		hypothetical protein CCA00942	cell envelope	Signal peptide	Signal peptide	No alpha-helix	unknown	p>0,01	<0	-	>2,965	No beta-barrel	No lipoprotein
CCA00949	29840704		hypothetical protein CCA00949	cell envelope	Signal peptide	Non-secretory protein	No alpha-helix	unknown	p>0,01	<0	-	>2,965	Beta-barrel	No lipoprotein
CCA00993	29840748		hypothetical protein CCA00993	cell envelope	Signal peptide	Signal peptide	No alpha-helix	unknown	p>0,01	<0	-	>2,965	Beta-barrel	No lipoprotein
***Chlamydophila pneumoniae* AR39**														
CP0022	16752316		hypothetical protein CP0022	cell envelope	Signal peptide	Non-secretory protein	No alpha-helix	unknown	p>0,01	<0	-	>2,965	No beta-barrel	No lipoprotein

CP0075	16752369		D-alanyl-D-alanine carboxypeptidase	cell envelope	Signal peptide	Signal peptide	2 alpha-helices	cytoplasmic membrane	p>0,01	<0	-	>2,965	No beta-barrel	No lipoprotein
CP0162	16752452		sigma-54 dependent response regulator	cell envelope	Non-secretory protein	Non-secretory protein	No alpha-helix	cytoplasmic	p>0,01	<0	-	>2,965	No beta-barrel	No lipoprotein
CP0170	16752460		hypothetical protein CP0170	cell envelope	Signal peptide	Non-secretory protein	No alpha-helix	unknown	p>0,01	<0	-	>2,965	No beta-barrel	No lipoprotein
CP0199	16752488		hypothetical protein CP0199	cell envelope	Signal peptide	Signal peptide	No alpha-helix	unknown	p>0,01	<0	-	>2,965	No beta-barrel	No lipoprotein
CP0327	16753071		hypothetical protein CP0327	cell envelope	Signal peptide	Non-secretory protein	No alpha-helix	unknown	p>0,01	<0	-	>2,965	No beta-barrel	No lipoprotein
CP0337	16752622		N-acetylmuramoyl-L-alanine amidase, putative	cell envelope	Non-secretory protein	Signal peptide	No alpha-helix	unknown	p>0,01	<0	-	>2,965	No beta-barrel	No lipoprotein
CP0417	16752699		hypothetical protein CP0417	cell envelope	Signal peptide	Signal peptide	No alpha-helix	unknown	p>0,01	<0	-	>2,965	No beta-barrel	No lipoprotein
CP0529	16753081		hypothetical protein CP0529	cell envelope	Signal peptide	Signal peptide	No alpha-helix	unknown	p>0,01	<0	-	>2,965	Beta-barrel	No lipoprotein
CP0578	16752851		hypothetical protein CP0578	cell envelope	Signal peptide	Signal peptide	1 alpha-helix	outer membrane	p>0,01	<0	-	>2,965	Beta-barrel	No lipoprotein
CP0620	16752892		hypothetical protein CP0620	cell envelope	Signal peptide	Signal peptide	No alpha-helix	unknown	p>0,01	<0	-	>2,965	No beta-barrel	No lipoprotein
CP0628	16752899		hypothetical protein CP0628	cell envelope	Signal peptide	Non-secretory protein	No alpha-helix	unknown	p>0,01	<0	-	>2,965	No beta-barrel	No lipoprotein
CP0690	16752960		hypothetical protein CP0690	cell envelope	Signal peptide	Signal peptide	No alpha-helix	unknown	p>0,01	<0	-	>2,965	No beta-barrel	No lipoprotein
CP0755	16753024		hypothetical protein CP0755	cell envelope	Signal peptide	Signal peptide	No alpha-helix	unknown	p>0,01	<0	-	2,955	No beta-barrel	No lipoprotein
CP0914	16752085		hypothetical protein CP0914	cell envelope	Signal peptide	Signal peptide	No alpha-helix	unknown	p>0,01	<0	-	2,896	No beta-barrel	No lipoprotein
CP0915	16752086		hypothetical protein CP0915	cell envelope	Signal peptide	Signal peptide	No alpha-helix	unknown	p>0,01	<0	-	>2,965	No beta-barrel	No lipoprotein
CP0917	16752088		hypothetical protein CP0917	cell envelope	Signal peptide	Signal peptide	No alpha-helix	unknown	p>0,01	<0	-	>2,965	No beta-barrel	No lipoprotein
CP0928	16752098		hypothetical protein CP0928	cell envelope	Signal peptide	Signal peptide	No alpha-helix	unknown	p>0,01	<0	-	>2,965	No beta-barrel	No lipoprotein
CP0940	16752110		hypothetical protein CP0940	cell envelope	Signal peptide	Signal peptide	No alpha-helix	unknown	p>0,01	<0	-	>2,965	No beta-barrel	No lipoprotein
CP0944	16752114		acylglycerophosphoethanolamine acyltransferase	cell envelope	Non-secretory protein	Non-secretory protein	1 alpha-helix	cytoplasmic membrane	p>0,01	<0	-	>2,965	No beta-barrel	No lipoprotein
CP0961	16752131		UDP-N-acetylmuramate--alanine ligase/D-alanine--D-alanine ligase	cell envelope	Non-secretory protein	Non-secretory protein	No alpha-helix	cytoplasmic	p>0,01	<0	-	>2,965	No beta-barrel	No lipoprotein
CP1050	16752219		hypothetical protein CP1050	cell envelope	Signal peptide	Signal peptide	No alpha-helix	unknown	p>0,01	<0	-	>2,965	No beta-barrel	No lipoprotein
CP1108	16752277		hypothetical protein CP1108	cell envelope	Signal peptide	Signal peptide	No alpha-helix	unknown	p>0,01	<0	-	>2,965	Beta-barrel	No lipoprotein
***Chlamydia trachomatis* D/UW-3/CX**														
CT041	15604760		hypothetical protein CT041	cell envelope	Signal peptide	Signal peptide	No alpha-helix	unknown	p>0,01	<0	-	>2,965	No beta-barrel	No lipoprotein
CT073	15604792		predicted OMP	cell envelope	Signal peptide	Signal peptide	No alpha-helix	unknown	p>0,01	<0	-	>2,965	No beta-barrel	No lipoprotein
CT109	15604828		CHLPS hypothetical protein	cell envelope	Signal peptide	Non-secretory protein	No alpha-helix	unknown	p>0,01	<0	-	>2,965	Beta-barrel	No lipoprotein
CT114	15604833		hypothetical protein CT114	cell envelope	Signal peptide	Non-secretory protein	No alpha-helix	outer membrane	p>0,01	<0	-	>2,965	No beta-barrel	No lipoprotein
CT131	15604850		(Possible Transmembrane Protein)	cell envelope	Signal peptide	Non-secretory protein	1 alpha-helix	outer membrane	p>0,01	<0	-	>2,965	Beta-barrel	No lipoprotein
CT149	15604868		possible hydrolase	cell envelope	Signal peptide	Signal peptide	No alpha-helix	unknown	p>0,01	<0	-	>2,965	No beta-barrel	No lipoprotein
CT181	15604901		hypothetical protein CT181	cell envelope	Signal peptide	Signal peptide	No alpha-helix	unknown	p>0,01	<0	-	>2,965	Beta-barrel	No lipoprotein
CT268	15604989	amiA	N-Acetylmuramoyl Alanine Amidase	cell envelope	Non-secretory protein	Signal peptide	No alpha-helix	unknown	p>0,01	<0	-	>2,965	No beta-barrel	No lipoprotein
CT271	15604992		hypothetical protein CT271	cell envelope	Signal peptide	Non-secretory protein	No alpha-helix	unknown	p>0,01	<0	-	>2,965	No beta-barrel	No lipoprotein
CT311	15605032		hypothetical protein CT311	cell envelope	Signal peptide	Signal peptide	No alpha-helix	unknown	p>0,01	<0	-	>2,965	No beta-barrel	No lipoprotein
CT360	15605084		hypothetical protein CT360	cell envelope	Signal peptide	Signal peptide	No alpha-helix	unknown	p>0,01	<0	-	>2,965	No beta-barrel	No lipoprotein
CT363	15605087	asd	aspartate-semialdehyde dehydrogenase	cell envelope	Signal peptide	Non-secretory protein	No alpha-helix	unknown	p>0,01	<0	-	>2,965	No beta-barrel	No lipoprotein
CT468	15605195	atoC	2-component regulatory system-ATPase	cell envelope	Non-secretory protein	Non-secretory protein	No alpha-helix	cytoplasmic	p>0,01	<0	-	>2,965	No beta-barrel	No lipoprotein
CT480.1	15605208		hypothetical protein CT480.1	cell envelope	Signal peptide	Signal peptide	No alpha-helix	unknown	p>0,01	<0	-	2,837	No beta-barrel	No lipoprotein

CT546	15605275		predicted OMP	cell envelope	Signal peptide	Non-secretory protein	No alpha-helix	unknown	p>0,01	<0	-	>2,965	No beta-barrel	No lipoprotein
CT551	15605280	dacC	D-Ala-D-Ala Carboxypeptidase	cell envelope	Signal peptide	Signal peptide	1 alpha-helix	cytoplasmic membrane	p>0,01	<0	-	>2,965	No beta-barrel	No lipoprotein
CT552	15605281		hypothetical protein CT552	cell envelope	Signal peptide	Signal peptide	No alpha-helix	unknown	p>0,01	<0	-	>2,965	No beta-barrel	No lipoprotein
CT566	15605295		hypothetical protein CT566	cell envelope	Signal peptide	Signal peptide	No alpha-helix	unknown	p>0,01	<0	-	>2,965	No beta-barrel	No lipoprotein
CT569	15605298		predicted OMP	cell envelope	Signal peptide	Non-secretory protein	No alpha-helix	unknown	p>0,01	<0	-	>2,965	No beta-barrel	No lipoprotein
CT573	15605302		hypothetical protein CT573	cell envelope	Signal peptide	Signal peptide	No alpha-helix	unknown	p>0,01	<0	-	>2,965	Beta-barrel	No lipoprotein
CT593.1	15605323		hypothetical protein CT593.1	cell envelope	Signal peptide	Non-secretory protein	No alpha-helix	unknown	p>0,01	<0	-	>2,965	No beta-barrel	No lipoprotein
CT648	15605380		hypothetical protein CT648	cell envelope	Signal peptide	Non-secretory protein	No alpha-helix	unknown	p>0,01	<0	-	>2,965	Beta-barrel	No lipoprotein
CT744	15605477		CHLTR possible phosphoprotein	cell envelope	Signal peptide	Signal peptide	No alpha-helix	unknown	p>0,01	<0	-	>2,965	Beta-barrel	No lipoprotein
CT759	15605492	nlpD	Muramidase (invasin repeat family)	cell envelope	Signal peptide	Signal peptide	1 alpha-helix	unknown	p>0,01	<0	-	>2,965	No beta-barrel	No lipoprotein
CT762	15605495	murC/ddlA	UDP-N-acetylmuramate-alanine ligase and D-Ala-D-Ala Ligase	cell envelope	Signal peptide	Signal peptide	1 alpha-helix	cytoplasmic	p>0,01	<0	-	>2,965	No beta-barrel	No lipoprotein
CT776	15605509	aas	acylglycerophosphoethanolamine acyltransferase	cell envelope	Non-secretory protein	Non-secretory protein	1 alpha-helix	cytoplasmic membrane	p>0,01	<0	-	>2,965	No beta-barrel	No lipoprotein
CT783	15605516		predicted disulfide bond isomerase	cell envelope	Signal peptide	Signal peptide	No alpha-helix	unknown	p>0,01	<0	-	>2,965	No beta-barrel	No lipoprotein
CT795	15605529		hypothetical protein CT795	cell envelope	Signal peptide	Signal peptide	No alpha-helix	unknown	p>0,01	<0	-	2,927	No beta-barrel	No lipoprotein
***Chlamydia muridarum* Nigg**														
TC0097	15834722		hypothetical protein TC0097	cell envelope	Signal peptide	Non-secretory protein	No alpha-helix	unknown	p>0,01	<0	-	>2,965	No beta-barrel	No lipoprotein
TC0120	15834744		hypothetical protein TC0120	cell envelope	Signal peptide	Signal peptide	No alpha-helix	unknown	p>0,01	<0	-	>2,965	Beta-barrel	No lipoprotein
TC0143	15834763	murC/ddlA	UDP-N-acetylmuramate--alanine ligase/D-alanine--D-alanine ligase	cell envelope	Non-secretory protein	Non-secretory protein	1 alpha-helix	cytoplasmic	p>0,01	<0	-	>2,965	No beta-barrel	No lipoprotein
TC0157	15834777		acylglycerophosphoethanolamine acyltransferase	cell envelope	Non-secretory protein	Non-secretory protein	No alpha-helix	cytoplasmic membrane	p>0,01	<0	-	>2,965	No beta-barrel	No lipoprotein
TC0160	15834780		hypothetical protein TC0160	cell envelope	Signal peptide	Non-secretory protein	No alpha-helix	unknown	p>0,01	<0	-	>2,965	No beta-barrel	No lipoprotein
TC0161	15834781		hypothetical protein TC0161	cell envelope	Signal peptide	Signal peptide	No alpha-helix	unknown	p>0,01	<0	-	>2,965	No beta-barrel	No lipoprotein
TC0166	15834786		hypothetical protein TC0166	cell envelope	Non-secretory protein	Signal peptide	No alpha-helix	unknown	p>0,01	<0	-	>2,965	No beta-barrel	No lipoprotein
TC0176	15834796		hypothetical protein TC0176	cell envelope	Signal peptide	Signal peptide	No alpha-helix	unknown	p>0,01	<0	-	2,937	No beta-barrel	No lipoprotein
TC0177	15834797		hypothetical protein TC0177	cell envelope	Signal peptide	Signal peptide	No alpha-helix	unknown	p>0,01	<0	-	2,946	No beta-barrel	No lipoprotein
TC0211	15834831		metalloprotease, insulinase family	cell envelope	Non-secretory protein	Non-secretory protein	No alpha-helix	outer membrane	p>0,01	<0	-	>2,965	No beta-barrel	No lipoprotein
TC0254	15834874		hypothetical protein TC0254	cell envelope	Signal peptide	Signal peptide	No alpha-helix	unknown	p>0,01	<0	-	>2,965	Beta-barrel	No lipoprotein
TC0286	15834906		hypothetical protein TC0286	cell envelope	Signal peptide	Signal peptide	No alpha-helix	unknown	p>0,01	<0	-	>2,965	No beta-barrel	No lipoprotein
TC0345	15834964		hypothetical protein TC0345	cell envelope	Signal peptide	Signal peptide	No alpha-helix	unknown	p>0,01	<0	-	>2,965	No beta-barrel	No lipoprotein
TC0354	15834973		hypothetical protein TC0354	cell envelope	Signal peptide	Non-secretory protein	No alpha-helix	unknown	p>0,01	<0	-	>2,965	Beta-barrel	No lipoprotein
TC0385	15835004		hypothetical protein TC0385	cell envelope	Signal peptide	Non-secretory protein	No alpha-helix	unknown	p>0,01	<0	-	>2,965	Beta-barrel	No lipoprotein
TC0408	15835026		hypothetical protein TC0408	cell envelope	Signal peptide	Signal peptide	1 alpha-helix	outer membrane	p>0,01	<0	-	>2,965	Beta-barrel	No lipoprotein
TC0426	15835044		hypothetical protein TC0426	cell envelope	Signal peptide	Signal peptide	No alpha-helix	unknown	p>0,01	<0	-	>2,965	No beta-barrel	No lipoprotein
TC0453	15835071		hypothetical protein TC0453	cell envelope	Signal peptide	Signal peptide	No alpha-helix	unknown	p>0,01	<0	-	>2,965	Beta-barrel	No lipoprotein
TC0534	15835152		hypothetical protein TC0534	cell envelope	Signal peptide	Non-secretory protein	No alpha-helix	unknown	p>0,01	<0	-	2,957	Beta-barrel	No lipoprotein

TC0539	15835157		N-acetylmuramoyl-L-alanine amidase, putative	cell envelope	Non-secretory protein	Non-secretory protein	No alpha-helix	unknown	p>0,01	<0	-	>2,965	No beta-barrel	No lipoprotein
TC0543	15835161		hypothetical protein TC0543	cell envelope	Signal peptide	Non-secretory protein	No alpha-helix	unknown	p>0,01	<0	-	>2,965	No beta-barrel	No lipoprotein
TC0585	15835202		hypothetical protein TC0585	cell envelope	Signal peptide	Signal peptide	No alpha-helix	unknown	p>0,01	<0	-	>2,965	No beta-barrel	No lipoprotein
TC0629	15835244		hypothetical protein TC0629	cell envelope	Signal peptide	Signal peptide	No alpha-helix	unknown	p>0,01	<0	-	2,926	No beta-barrel	Cytoplasmic membrane protein
TC0735	15835350	uppS	undecaprenyl pyrophosphate synthetase	cell envelope	Non-secretory protein	Non-secretory protein	No alpha-helix	unknown	p>0,01	<0	-	>2,965	No beta-barrel	No lipoprotein
TC0753	15835367		sigma-54 dependent response regulator	cell envelope	Non-secretory protein	Non-secretory protein	No alpha-helix	cytoplasmic	p>0,01	<0	-	>2,965	No beta-barrel	No lipoprotein
TC0834	15835448		hypothetical protein TC0834	cell envelope	Signal peptide	Non-secretory protein	No alpha-helix	unknown	p>0,01	<0	-	>2,965	No beta-barrel	No lipoprotein
TC0839	15835453		D-alanyl-D-alanine carboxypeptidase, putative	cell envelope	Signal peptide	Signal peptide	1 alpha-helix	cytoplasmic membrane	p>0,01	<0	-	>2,965	No beta-barrel	No lipoprotein
TC0840	15835454		hypothetical protein TC0840	cell envelope	Signal peptide	Signal peptide	No alpha-helix	unknown	p>0,01	<0	-	>2,965	No beta-barrel	No lipoprotein
TC0855	15835469		hypothetical protein TC0855	cell envelope	Signal peptide	Signal peptide	No alpha-helix	unknown	p>0,01	<0	-	>2,965	No beta-barrel	No lipoprotein
***Protochlamydia amoebophila* UWE25**														
pc0025	46445659		conserved hypothetical protein	cell envelope	Signal peptide	Non-secretory protein	No alpha-helix	unknown	p>0,01	<0	-	>2,965	No beta-barrel	No lipoprotein
pc0026	46445660		conserved hypothetical protein	cell envelope	Signal peptide	Signal peptide	No alpha-helix	outer membrane	p>0,01	<0	-	>2,965	Beta-barrel	No lipoprotein
pc0116	46445750		unknown protein	cell envelope	Non-secretory protein	Signal peptide	No alpha-helix	unknown	p>0,01	<0	-	>2,965	No beta-barrel	No lipoprotein
pc0214	46445848	tsp	similar to carboxy-terminal (= tail-specific) proteinase	cell envelope	Signal peptide	Signal peptide	No alpha-helix	unknown	p>0,01	<0	-	>2,965	No beta-barrel	No lipoprotein
pc0225	46445859		conserved hypothetical protein	cell envelope	Signal peptide	Signal peptide	No alpha-helix	unknown	p>0,01	<0	-	>2,965	No beta-barrel	No lipoprotein
pc0305	46445939	comF	similar to competence-related protein comF	cell envelope	Signal peptide	Non-secretory protein	No alpha-helix	unknown	p>0,01	<0	-	>2,965	No beta-barrel	No lipoprotein
pc0315	46445949	amiA	similar to N-acetylmuramoyl-L-alanine amidase	cell envelope	Signal peptide	Signal peptide	No alpha-helix	unknown	p>0,01	<0	-	>2,965	No beta-barrel	No lipoprotein
pc0326	46445960	ykuE	conserved hypothetical protein	cell envelope	Signal peptide	Non-secretory protein	No alpha-helix	unknown	p>0,01	<0	-	>2,965	No beta-barrel	No lipoprotein
pc0332	46445966		hypothetical protein	cell envelope	Signal peptide	Signal peptide	No alpha-helix	unknown	p>0,01	<0	-	>2,965	Beta-barrel	No lipoprotein
pc0354	46445988		unknown protein	cell envelope	Signal peptide	Non-secretory protein	No alpha-helix	unknown	p>0,01	<0	-	>2,965	No beta-barrel	No lipoprotein
pc0363	46445997		unknown protein	cell envelope	Signal peptide	Signal peptide	No alpha-helix	unknown	p>0,01	<0	-	>2,965	Beta-barrel	No lipoprotein
pc0394	46446028	dac	similar to serine-type D-Ala-D-Ala carboxypeptidase (penicillin binding protein)	cell envelope	Non-secretory protein	Non-secretory protein	1 alpha-helix	cytoplasmic membrane	p>0,01	<0	-	>2,965	No beta-barrel	No lipoprotein
pc0452	46446086		hypothetical protein	cell envelope	Signal peptide	Non-secretory protein	1 alpha-helix	unknown	p>0,01	<0	-	>2,965	No beta-barrel	No lipoprotein
pc0497	46446131		hypothetical protein	cell envelope	Signal peptide	Signal peptide	No alpha-helix	unknown	p>0,01	<0	-	>2,965	Beta-barrel	No lipoprotein
pc0500	46446134		conserved hypothetical protein	cell envelope	Signal peptide	Non-secretory protein	No alpha-helix	unknown	p>0,01	<0	-	>2,965	No beta-barrel	No lipoprotein
pc0501	46446135		conserved hypothetical protein	cell envelope	Signal peptide	Signal peptide	No alpha-helix	unknown	p>0,01	<0	-	>2,965	Beta-barrel	No lipoprotein
pc0538	46446172	rtxA	similar to RTX-toxin, partial length	cell envelope	Non-secretory protein	Non-secretory protein	No alpha-helix	unknown	p>0,01	<0	-	>2,965	No beta-barrel	No lipoprotein
pc0553	46446187		hypothetical protein	cell envelope	Signal peptide	Signal peptide	No alpha-helix	unknown	p>0,01	0,007	-	>2,965	No beta-barrel	No lipoprotein
pc0575	46446209		conserved hypothetical protein	cell envelope	Signal peptide	Signal peptide	No alpha-helix	unknown	p>0,01	<0	-	>2,965	Beta-barrel	No lipoprotein
pc0585	46446219		hypothetical protein	cell envelope	Signal peptide	Signal peptide	No alpha-helix	unknown	p>0,01	<0	-	>2,965	No beta-barrel	No lipoprotein
pc0609	46446243		hypothetical protein	cell envelope	Signal peptide	Signal peptide	No alpha-helix	unknown	p>0,01	<0	-	>2,965	No beta-barrel	No lipoprotein
pc0719	46446353		similar to serine protease	cell envelope	Signal peptide	Signal peptide	No alpha-helix	unknown	p>0,01	<0	-	>2,965	No beta-barrel	No lipoprotein

pc0773	46446407		unknown protein	cell envelope	Signal peptide	Signal peptide	No alpha-helix	unknown	p>0,01	<0	-	>2,965	Beta-barrel	No lipoprotein
pc0839	46446473		unknown protein	cell envelope	Signal peptide	Non-secretory protein	No alpha-helix	unknown	p>0,01	<0	-	>2,965	Beta-barrel	No lipoprotein
pc0885	46446519		unknown protein	cell envelope	Signal peptide	Signal peptide	No alpha-helix	unknown	p>0,01	<0	-	>2,965	Beta-barrel	No lipoprotein
pc0945	46446579		unknown protein	cell envelope	Signal peptide	Signal peptide	No alpha-helix	unknown	p>0,01	<0	-	>2,965	Beta-barrel	No lipoprotein
pc0977	46446611		unknown protein	cell envelope	Signal peptide	Signal peptide	No alpha-helix	unknown	p>0,01	0,02	-	>2,965	No beta-barrel	No lipoprotein
pc1061	46446695		conserved hypothetical protein	cell envelope	Signal peptide	Non-secretory protein	No alpha-helix	unknown	p>0,01	<0	-	>2,965	No beta-barrel	No lipoprotein
pc1067	46446701		conserved hypothetical protein	cell envelope	Signal peptide	Non-secretory protein	No alpha-helix	unknown	p>0,01	<0	-	>2,965	No beta-barrel	No lipoprotein
pc1091	46446725		unknown protein	cell envelope	Signal peptide	Signal peptide	No alpha-helix	unknown	p>0,01	<0	-	>2,965	Beta-barrel	No lipoprotein
pc1099	46446733		conserved hypothetical protein	cell envelope	Signal peptide	Non-secretory protein	No alpha-helix	unknown	p>0,01	<0	-	>2,965	No beta-barrel	No lipoprotein
pc1107	46446741		hypothetical protein	cell envelope	Signal peptide	Signal peptide	No alpha-helix	unknown	p>0,01	<0	-	2,928	No beta-barrel	No lipoprotein
pc1174	46446808		unknown protein	cell envelope	Signal peptide	Signal peptide	No alpha-helix	unknown	p>0,01	<0	-	>2,965	Beta-barrel	No lipoprotein
pc1208	46446842		unknown protein	cell envelope	Signal peptide	Non-secretory protein	No alpha-helix	unknown	p>0,01	<0	-	>2,965	Beta-barrel	No lipoprotein
pc1236	46446870	aas	similar to bifunctional AAS protein	cell envelope	Non-secretory protein	Signal peptide	No alpha-helix	cytoplasmic membrane	p>0,01	<0	-	>2,965	No beta-barrel	No lipoprotein
pc1247	46446881	murC	similar to UDP-N-acetylmuramate-alanine ligase murC	cell envelope	Non-secretory protein	Non-secretory protein	No alpha-helix	cytoplasmic	p>0,01	<0	-	>2,965	No beta-barrel	No lipoprotein
pc1250	46446884	lytF	similar to muropeptidase (autolysin)	cell envelope	Signal peptide	Signal peptide	1 alpha-helix	unknown	p>0,01	<0	-	2,931	No beta-barrel	No lipoprotein
pc1337	46446971		conserved hypothetical protein	cell envelope	Signal peptide	Signal peptide	No alpha-helix	unknown	p>0,01	<0	-	>2,965	No beta-barrel	No lipoprotein
pc1419	46447053		hypothetical protein	cell envelope	Signal peptide	Non-secretory protein	No alpha-helix	unknown	p>0,01	<0	-	>2,965	No beta-barrel	No lipoprotein
pc1424	46447058		unknown protein	cell envelope	Signal peptide	Signal peptide	No alpha-helix	unknown	p>0,01	<0	-	>2,965	No beta-barrel	No lipoprotein
pc1428	46447062		unknown protein	cell envelope	Signal peptide	Non-secretory protein	No alpha-helix	unknown	p>0,01	<0	-	>2,965	Beta-barrel	No lipoprotein
pc1447	46447081		unknown protein	cell envelope	Signal peptide	Signal peptide	No alpha-helix	unknown	p>0,01	<0	-	>2,965	No beta-barrel	No lipoprotein
pc1448	46447082		unknown protein	cell envelope	Signal peptide	Signal peptide	No alpha-helix	unknown	p>0,01	<0	-	>2,965	Beta-barrel	No lipoprotein
pc1476	46447110		unknown protein	cell envelope	Signal peptide	Non-secretory protein	No alpha-helix	unknown	p>0,01	<0	-	>2,965	No beta-barrel	No lipoprotein
pc1477	46447111		conserved hypothetical protein, partial length	cell envelope	Signal peptide	Non-secretory protein	No alpha-helix	unknown	p>0,01	<0	-	>2,965	No beta-barrel	No lipoprotein
pc1546	46447180		unknown protein	cell envelope	Signal peptide	Signal peptide	No alpha-helix	unknown	p>0,01	<0	-	>2,965	Beta-barrel	No lipoprotein
pc1600	46447234		hypothetical protein	cell envelope	Signal peptide	Signal peptide	No alpha-helix	unknown	p>0,01	<0	-	>2,965	No beta-barrel	No lipoprotein
pc1683	46447317		hypothetical protein	cell envelope	Signal peptide	Signal peptide	No alpha-helix	unknown	p>0,01	<0	-	>2,965	Beta-barrel	No lipoprotein
pc1688	46447322		hypothetical protein	cell envelope	Signal peptide	Non-secretory protein	No alpha-helix	unknown	p>0,01	<0	-	>2,965	No beta-barrel	No lipoprotein
pc1769	46447403		hypothetical protein	cell envelope	Signal peptide	Non-secretory protein	No alpha-helix	unknown	p>0,01	<0	-	>2,965	No beta-barrel	No lipoprotein
pc1991	46447625		conserved hypothetical protein	cell envelope	Signal peptide	Signal peptide	No alpha-helix	unknown	p>0,01	<0	-	>2,965	Beta-barrel	No lipoprotein

Table S6: Overview of prediction results used for classification. Plus; positive prediction result obligatory; minus, negative prediction result obligatory; ~, prediction result has no influence; $+^*$, one of the predictions labelled as such has to be positive.

	Integral	Integral (putative)	Lipoprotein	Lipoprotein (putative)	Component	Ambiguous
**Signal peptide**	$+^*$	$+^*$	~	$+^*$	$+^*$	-
**cpsortB outer membrane**	$+^*$	$+^*$	~	$+^*$	$+^*$	-
**Annotation outer membrane**	$+^*$	$+^*$	~	$+^*$	$+^*$	-
**Beta-barrel ≥2 out of 4 or beta-helix**	+	-	~	~	-	-
**Beta barrel 1**	-	$+^*$	~	~	$+^*$	-
**In integral /mixed cluster**	+	+	-	-	-	~
**Lipoprotein**	-	-	+	-	-	-
**In lipoprotein cluster**	-	-	+	+	-	~
**Transmembrane helix**	-	-	-	-	-	~
**Annotation cytoplasm, cytoplasmic membrane, periplasm**	-	-	-	-	-	~

Table S7: Ingroup and outgroup organisms used for cluster formation. Taxonomic affiliation, lifestyle and obligate intracellular pathogenicity for humans are indicated.

Organism	Intracellular	Phylum	Human pathogen	Ingroup/ Outgroup	Genome size [nt]
Anaplasma marginale str. St. Maries	obligate	Alphaproteobacteria		outgroup	1197687
Anaplasma phagocytophilum HZ	obligate	Alphaproteobacteria	yes	outgroup	1471282
Aster yellows witches'-broom phytoplasma AYWB	obligate	Firmicutes		outgroup	723970
Baumannia cicadellinicola str. Hc (Homalodisca coagulata)	obligate	Gammaproteobacteria		outgroup	686194
Buchnera aphidicola str. APS (Acyrthosiphon pisum)	obligate	Gammaproteobacteria		outgroup	640681
Buchnera aphidicola str. Bp (Baizongia pistaciae)	obligate	Gammaproteobacteria		outgroup	618379
Buchnera aphidicola str. Cc (Cinara cedri)	obligate	Gammaproteobacteria		outgroup	416380
Buchnera aphidicola str. Sg (Schizaphis graminum)	obligate	Gammaproteobacteria		outgroup	641454
Candidatus Amoebophilus asiaticus 5a2	obligate	Bacteroidetes		outgroup	1837115
Candidatus Blochmannia floridanus	obligate	Gammaproteobacteria		outgroup	705557
Candidatus Blochmannia pennsylvanicus str. BPEN	obligate	Gammaproteobacteria		outgroup	791654
Candidatus Carsonella ruddii PV	obligate	Gammaproteobacteria		outgroup	159662
Candidatus Ruthia magnifica str. Cm (Calyptogena magnifica)	obligate	unknown Proteobacteria		outgroup	1160782
Chlamydia muridarum Nigg	obligate	Chlamydia		ingroup1	1080451
Chlamydia trachomatis A/HAR-13	obligate	Chlamydia	yes	ingroup1	1051969
Chlamydia trachomatis D/UW-3/CX	obligate	Chlamydia	yes	ingroup1	1042519
Chlamydophila abortus S26/3	obligate	Chlamydia		ingroup1	1144377
Chlamydophila caviae GPIC	obligate	Chlamydia		ingroup1	1181356
Chlamydophila felis Fe/C-56	obligate	Chlamydia		ingroup1	1173791
Chlamydophila pneumoniae AR39	obligate	Chlamydia	yes	ingroup1	1229853
Coxiella burnetii RSA 493	obligate	Gammaproteobacteria	yes	outgroup	2032674
Ehrlichia canis str. Jake	obligate	Alphaproteobacteria	yes	outgroup	1315030
Ehrlichia chaffeensis str. Arkansas	obligate	Alphaproteobacteria	yes	outgroup	1176248
Ehrlichia ruminantium str. Gardel	obligate	Alphaproteobacteria		outgroup	1499920
Ehrlichia ruminantium str. Welgevonden	obligate	Alphaproteobacteria		outgroup	1512977
Lawsonia intracellularis PHE/MN1-00	obligate	Deltaproteobacteria		outgroup	1457619
Mycobacterium leprae TN	obligate	Actinobacteria	yes	outgroup	3268203
Neorickettsia sennetsu str. Miyayama	obligate	Alphaproteobacteria	yes	outgroup	859006
Onion yellows phytoplasma OY-M	obligate	Firmicutes		outgroup	860631
Parachlamydia acanthamoebae	obligate	Chlamydia		ingroup1	3015107
Candidatus Protochlamydia amoebophila UWE25	obligate	Chlamydia		ingroup1	2414465
Rickettsia bellii RML369-C	obligate	Alphaproteobacteria		outgroup	1522076
Rickettsia conorii str. Malish 7	obligate	Alphaproteobacteria	yes	outgroup	1268755
Rickettsia felis URRWXCal2	obligate	Alphaproteobacteria	yes	outgroup	1587240
Rickettsia prowazekii str. Madrid E	obligate	Alphaproteobacteria	yes	outgroup	1111523
Rickettsia typhi str. Wilmington	obligate	Alphaproteobacteria	yes	outgroup	1111496
Simkania negevensis	obligate	Chlamydia		ingroup1	2628375
Sodalis glossinidius str. 'morsitans'	obligate	Gammaproteobacteria		outgroup	4292502
Ureaplasma parvum serovar 3 str. ATCC 700970	obligate	Firmicutes	yes	outgroup	751719
Waddlia chondrophila	obligate	Chlamydia		ingroup1	2141577
Wigglesworthia glossinidia endosymbiont of Glossina brevipalpis	obligate	Gammaproteobacteria		outgroup	697724
Wolbachia endosymbiont of Drosophila melanogaster	obligate	Alphaproteobacteria		outgroup	1267782

Wolbachia endosymbiont strain TRS of Brugia malayi	obligate	Alphaproteobacteria		outgroup	1080084
Bartonella henselae str. Houston-1	facultative	Alphaproteobacteria		outgroup	1931047
Bartonella quintana str. Toulouse	facultative	Alphaproteobacteria		outgroup	1581384
Brucella abortus biovar 1 str. 9-941	facultative	Alphaproteobacteria		outgroup	3286445
Brucella melitensis 16M	facultative	Alphaproteobacteria		outgroup	3294931
Brucella melitensis biovar Abortus 2308	facultative	Alphaproteobacteria		outgroup	3278307
Brucella suis 1330	facultative	Alphaproteobacteria		outgroup	3315175
Burkholderia pseudomallei 1710b	facultative	Betaproteobacteria		outgroup	7308054
Burkholderia pseudomallei k96243	facultative	Betaproteobacteria		outgroup	7247547
Francisella tularensis subsp. holarctica	facultative	Gammaproteobacteria		outgroup	1895994
Francisella tularensis subsp. holarctica OSU18	facultative	Gammaproteobacteria		outgroup	1895727
Francisella tularensis subsp. novicida U112	facultative	Gammaproteobacteria		outgroup	1910031
Francisella tularensis subsp. tularensis FSC 198	facultative	Gammaproteobacteria		outgroup	1892616
Francisella tularensis subsp. tularensis SCHU S4	facultative	Gammaproteobacteria		outgroup	1892819
Legionella pneumophila str. Lens	facultative	Gammaproteobacteria		outgroup	3405519
Legionella pneumophila str. Paris	facultative	Gammaproteobacteria		outgroup	3635495
Legionella pneumophila subsp. pneumophila str. Philadelphia 1	facultative	Gammaproteobacteria		outgroup	3397754
Listeria monocytogenes EGD-e	facultative	Firmicutes		outgroup	2944528
Listeria monocytogenes str. 4b F2365	facultative	Firmicutes		outgroup	2905187
Mesorhizobium loti MAFF303099	facultative	Alphaproteobacteria		outgroup	7596297
Mesorhizobium sp. BNC1	facultative	Alphaproteobacteria		outgroup	4935185
Mycobacterium avium 104	facultative	Actinobacteria		outgroup	5475491
Mycobacterium avium subsp. paratuberculosis K-10	facultative	Actinobacteria		outgroup	4829781
Mycobacterium bovis AF2122/97	facultative	Actinobacteria		outgroup	4345492
Mycobacterium marinum M	facultative	Actinobacteria		outgroup	6660144
Mycobacterium tuberculosis CDC1551	facultative	Actinobacteria		outgroup	4403837
Mycobacterium tuberculosis H37RV	facultative	Actinobacteria		outgroup	4411532
Mycoplasma penetrans HF-2	facultative	Firmicutes		outgroup	1358633
Neisseria gonorrhoeae FA 1090	facultative	Betaproteobacteria		outgroup	2153922
Neisseria lactamica	facultative	Betaproteobacteria		outgroup	2223456
Neisseria meningitidis MC58	facultative	Betaproteobacteria		outgroup	2272360
Neisseria meningitidis Z2491	facultative	Betaproteobacteria		outgroup	2184406
Nocardia farcinica IFM 10152	facultative	Actinobacteria		outgroup	6292344
Pasteurella multocida subsp. multocida str. Pm70	facultative	Gammaproteobacteria		outgroup	2257487
Porphyromonas gingivalis W83	facultative	Bacteroidetes		outgroup	2343476
Pseudomonas aeruginosa PA01	facultative	Gammaproteobacteria		outgroup	6264404
Pseudomonas aeruginosa UCBPP-PA14	facultative	Gammaproteobacteria		outgroup	6537648
Rhizobium etli CFN 42	facultative	Alphaproteobacteria		outgroup	6530228
Rhizobium leguminosarum bv. viciae 3841	facultative	Alphaproteobacteria		outgroup	7751309
Salmonella enterica subsp. enterica serovar Choleraesuis str. SC-B67	facultative	Gammaproteobacteria		ingroup2	4944000
Salmonella enterica subsp. enterica serovar Gallinarum	facultative	Gammaproteobacteria		ingroup2	4658697
Salmonella enterica subsp. enterica serovar Paratyphi A str. ATCC 9150	facultative	Gammaproteobacteria		ingroup2	4585229
Salmonella enterica subsp. enterica serovar Typhi str. CT18	facultative	Gammaproteobacteria		ingroup2	5133713
Salmonella enterica subsp. enterica serovar Typhi Ty2	facultative	Gammaproteobacteria		ingroup2	4791961
Salmonella enteritidis	facultative	Gammaproteobacteria		ingroup2	4685848
Salmonella typhimurium LT2	facultative	Gammaproteobacteria		ingroup2	4951371
Shigella boydii Sb227	facultative	Gammaproteobacteria		ingroup2	4646520

Shigella dysenteriae Sd197	facultative	Gammaproteobacteria		ingroup2	4551958
Shigella flexneri 2a str. 2457T	facultative	Gammaproteobacteria		ingroup2	4599354
Shigella flexneri 2a str. 301	facultative	Gammaproteobacteria		ingroup2	4828821
Shigella flexneri 5 str. 8401	facultative	Gammaproteobacteria		ingroup2	4574284
Shigella sonnei Ss046	facultative	Gammaproteobacteria		ingroup2	5039661
Sinorhizobium medicae WSM419	facultative	Alphaproteobacteria		outgroup	6817576
Sinorhizobium meliloti 1021	facultative	Alphaproteobacteria		outgroup	6691694
Streptococcus agalactiae 2603V/R	facultative	Firmicutes		outgroup	2160267
Streptococcus agalactiae A909	facultative	Firmicutes		outgroup	2127839
Streptococcus agalactiae NEM316	facultative	Firmicutes		outgroup	2211485
Streptococcus pyogenes M1 GAS	facultative	Firmicutes		outgroup	1852441
Streptococcus pyogenes MGAS10270	facultative	Firmicutes		outgroup	1928252
Streptococcus pyogenes MGAS10394	facultative	Firmicutes		outgroup	1899877
Streptococcus pyogenes MGAS10750	facultative	Firmicutes		outgroup	1937111
Streptococcus pyogenes MGAS2096	facultative	Firmicutes		outgroup	1860355
Streptococcus pyogenes MGAS315	facultative	Firmicutes		outgroup	1900521
Streptococcus pyogenes MGAS5005	facultative	Firmicutes		outgroup	1838554
Streptococcus pyogenes MGAS6180	facultative	Firmicutes		outgroup	1897573
Streptococcus pyogenes MGAS8232	facultative	Firmicutes		outgroup	1895017
Streptococcus pyogenes MGAS9429	facultative	Firmicutes		outgroup	1836467
Streptococcus pyogenes SSI-1	facultative	Firmicutes		outgroup	1894275
Treponema pallidum subsp. pallidum str. Nichols	facultative	Spirochaetes		outgroup	1138011
Tropheryma whipplei str. Twist	facultative	Actinobacteria		outgroup	927303
Tropheryma whipplei TW08/27	facultative	Actinobacteria		outgroup	925938
Vibrio cholerae O1 biovar eltor str. N16961	facultative	Gammaproteobacteria		outgroup	4033464
Yersinia pestis Antiqua	facultative	Gammaproteobacteria		outgroup	4879836
Yersinia pestis biovar Microtus str. 91001	facultative	Gammaproteobacteria		outgroup	4803217
Yersinia pestis CO92	facultative	Gammaproteobacteria		outgroup	4829855
Yersinia pestis KIM	facultative	Gammaproteobacteria		outgroup	4953402
Yersinia pestis Nepal516	facultative	Gammaproteobacteria		outgroup	4646286
Yersinia pseudotuberculosis IP 32953	facultative	Gammaproteobacteria		outgroup	4840898
Acidobacteria bacterium Ellin345	no	Acidobacteria		outgroup	5650368
Acidothermus cellulolyticus 11B	no	Actinobacteria		outgroup	2443540
Acidovorax avenae subsp. citrulli AAC00-1	no	Betaproteobacteria		outgroup	5352772
Acinetobacter sp. ADP1	no	Gammaproteobacteria		outgroup	3598621
Aeromonas hydrophila subsp. hydrophila ATCC 7966	no	Gammaproteobacteria		outgroup	4744448
Aeropyrum pernix K1	no	Crenarchaeota		outgroup	1669695
Agrobacterium tumefaciens str. C58	no	Alphaproteobacteria		outgroup	5673465
Alcanivorax borkumensis SK2	no	Gammaproteobacteria		outgroup	3120143
Alkalilimnicola ehrlichei MLHE-1	no	Gammaproteobacteria		outgroup	3275944
Anabaena variabilis ATCC 29413	no	Cyanobacteria		outgroup	7068601
Anaeromyxobacter dehalogenans 2CP-C	no	Deltaproteobacteria		outgroup	5013479
Aquifex aeolicus VF5	no	Aquificae		outgroup	1590791
Archaeoglobus fulgidus DSM 4304	no	Euryarchaeota		outgroup	2178400
Arthrobacter aurescens TC1	no	Actinobacteria		outgroup	5226648
Arthrobacter sp. FB24	no	Actinobacteria		outgroup	5070478
Azoarcus sp. BH72	no	Betaproteobacteria		outgroup	4376040
Azoarcus sp. EbN1	no	Betaproteobacteria		outgroup	4727255
Bacillus anthracis str. 'Ames Ancestor'	no	Firmicutes		outgroup	5503926
Bacillus anthracis str. Ames	no	Firmicutes		outgroup	5227293
Bacillus anthracis str. Sterne	no	Firmicutes		outgroup	5228663

Bacillus cereus ATCC 10987	no	Firmicutes		outgroup	5432652
Bacillus cereus ATCC 14579	no	Firmicutes		outgroup	5427083
Bacillus cereus E33L	no	Firmicutes		outgroup	5843235
Bacillus clausii KSM-K16	no	Firmicutes		outgroup	4303871
Bacillus halodurans C-125	no	Firmicutes		outgroup	4202352
Bacillus licheniformis ATCC 14580	no	Firmicutes		outgroup	4222645
Bacillus subtilis subsp. subtilis str. 168	no	Firmicutes		outgroup	4214630
Bacillus thuringiensis serovar konkukian str. 97-27	no	Firmicutes		outgroup	5314794
Bacillus thuringiensis str. Al Hakam	no	Firmicutes		outgroup	5313030
Bacteroides fragilis NCTC 9343	no	Bacteroidetes		outgroup	5241700
Bacteroides fragilis YCH46	no	Bacteroidetes		outgroup	5310990
Bacteroides thetaiotaomicron VPI-5482	no	Bacteroidetes		outgroup	6293399
Bdellovibrio bacteriovorus HD100	no	Deltaproteobacteria		outgroup	3782950
Bifidobacterium adolescentis ATCC 15703	no	Actinobacteria		outgroup	2089645
Bifidobacterium longum NCC2705	no	Actinobacteria		outgroup	2260266
Blastopirellula marina DSM 3645	no	Planctomycetes		outgroup	6653746
Bordetella bronchiseptica RB50	no	Betaproteobacteria		outgroup	5339179
Bordetella parapertussis 12822	no	Betaproteobacteria		outgroup	4773551
Bordetella pertussis Tohama I	no	Betaproteobacteria		outgroup	4086189
Borrelia afzelii PKO	no	Spirochaetes		outgroup	1145124
Borrelia burgdorferi B31	no	Spirochaetes		outgroup	1519856
Borrelia garinii PBi	no	Spirochaetes		outgroup	986914
Bradyrhizobium japonicum USDA 110	no	Alphaproteobacteria		outgroup	9105828
Bradyrhizobium sp. BTAi1	no	Alphaproteobacteria		outgroup	8493513
Bradyrhizobium sp. ORS278	no	Alphaproteobacteria		outgroup	7456587
Burkholderia cenocepacia AU 1054	no	Betaproteobacteria		outgroup	7279116
Burkholderia cenocepacia HI2424	no	Betaproteobacteria		outgroup	7702840
Burkholderia cepacia AMMD	no	Betaproteobacteria		outgroup	7528567
Burkholderia mallei ATCC 23344	no	Betaproteobacteria		outgroup	5835527
Burkholderia sp. 383	no	Betaproteobacteria		outgroup	8676277
Burkholderia thailandensis E264	no	Betaproteobacteria		outgroup	6723972
Burkholderia xenovorans LB400	no	Betaproteobacteria		outgroup	9731138
Campylobacter fetus subsp. fetus 82-40	no	Epsilonproteobacteria		outgroup	1773615
Campylobacter jejuni RM1221	no	Epsilonproteobacteria		outgroup	1777831
Campylobacter jejuni subsp. jejuni NCTC 11168	no	Epsilonproteobacteria		outgroup	1641481
Candidatus Kuenenia stuttgartiensis	no	Planctomycetes		outgroup	4218325
Candidatus Pelagibacter ubique HTCC1062	no	Alphaproteobacteria		outgroup	1308759
Carboxydothermus hydrogenoformans Z-2901	no	Firmicutes		outgroup	2401520
Caulobacter crescentus CB15	no	Alphaproteobacteria		outgroup	4016947
Chlorobium chlorochromatii CAD3	no	Chlorobi		outgroup	2572079
Chlorobium phaeobacteroides DSM 266	no	Chlorobi		outgroup	3133902
Chlorobium tepidum TLS	no	Chlorobi		outgroup	2154946
Chromobacterium violaceum ATCC 12472	no	Betaproteobacteria		outgroup	4751080
Chromohalobacter salexigens DSM 3043	no	Gammaproteobacteria		outgroup	3696649
Clostridium acetobutylicum ATCC 824	no	Firmicutes		outgroup	4132880
Clostridium novyi NT	no	Firmicutes		outgroup	2547720
Clostridium perfringens ATCC 13124	no	Firmicutes		outgroup	3256683
Clostridium perfringens SM101	no	Firmicutes		outgroup	2921996
Clostridium perfringens str. 13	no	Firmicutes		outgroup	3085740
Clostridium tetani E88	no	Firmicutes		outgroup	2873333
Colwellia psychrerythraea 34H	no	Gammaproteobacteria		outgroup	5373180

Corynebacterium diphtheriae NCtC 13129	no	Actinobacteria		outgroup	2488635
Corynebacterium efficiens YS-314	no	Actinobacteria		outgroup	3147090
Corynebacterium glutamicum ATCC 13032	no	Actinobacteria		outgroup	3282708
Corynebacterium jeikeium K411	no	Actinobacteria		outgroup	2476822
Cytophaga hutchinsonii ATCC 33406	no	Bacteroidetes		outgroup	4433218
Dechloromonas aromatica RCB	no	Betaproteobacteria		outgroup	4501104
Dehalococcoides ethenogenes 195	no	Chloroflexi		outgroup	1469720
Dehalococcoides sp. CBDB1	no	Chloroflexi		outgroup	1395502
Deinococcus geothermalis DSM 11300	no	Deinococcus-Thermus		outgroup	3041332
Deinococcus radiodurans R1	no	Deinococcus-Thermus		outgroup	3284156
Desulfitobacterium hafniense Y51	no	Firmicutes		outgroup	5727534
Desulfotalea psychrophila LSV54	no	Deltaproteobacteria		outgroup	3659634
Desulfovibrio desulfuricans G20	no	Deltaproteobacteria		outgroup	3730232
Desulfovibrio vulgaris subsp. vulgaris DP4	no	Deltaproteobacteria		outgroup	3661391
Desulfovibrio vulgaris subsp. vulgaris str. Hildenborough	no	Deltaproteobacteria		outgroup	3773159
Enterobacter sakazakii	no	Gammaproteobacteria		outgroup	4530777
Enterococcus faecalis V583	no	Firmicutes		outgroup	3359974
Erwinia carotovora subsp. atroseptica SCRI1043	no	Gammaproteobacteria		outgroup	5064019
Erythrobacter litoralis HTCC2594	no	Alphaproteobacteria		outgroup	3052398
Escherichia coli 536	no	Gammaproteobacteria		ingroup2	4938920
Escherichia coli APEC O1	no	Gammaproteobacteria		ingroup2	5082025
Escherichia coli CFT073	no	Gammaproteobacteria		ingroup2	5231428
Escherichia coli K12	no	Gammaproteobacteria		ingroup2	4639675
Escherichia coli O157:H7 EDL933	no	Gammaproteobacteria		ingroup2	5528445
Escherichia coli O157:H7 str. Sakai	no	Gammaproteobacteria		ingroup2	5594477
Escherichia coli UTI89	no	Gammaproteobacteria		ingroup2	5179971
Escherichia coli W3110	no	Gammaproteobacteria		ingroup2	4646332
Frankia alni ACN14a	no	Actinobacteria		outgroup	7497934
Frankia sp. CcI3	no	Actinobacteria		outgroup	5433628
Fusobacterium nucleatum subsp. nucleatum ATCC 25586	no	Fusobacteria		outgroup	2174500
Gemmata obscuriglobus	no	Planctomycetes		outgroup	9161841
Geobacillus kaustophilus HTA426	no	Firmicutes		outgroup	3592666
Geobacter metallireducens GS-15	no	Deltaproteobacteria		outgroup	4011182
Geobacter sulfurreducens PCA	no	Deltaproteobacteria		outgroup	3814139
Gloeobacter violaceus PCC 7421	no	Cyanobacteria		outgroup	4659019
Gluconobacter oxydans 621H	no	Alphaproteobacteria		outgroup	2922384
Gramella forsetii KT0803	no	Bacteroidetes		outgroup	3798465
Granulibacter bethesdensis CGDNIH1	no	Alphaproteobacteria		outgroup	2708355
Haemophilus ducreyi 35000HP	no	Gammaproteobacteria		outgroup	1698955
Haemophilus influenzae 86-028NP	no	Gammaproteobacteria		outgroup	1914490
Haemophilus influenzae Rd KW20	no	Gammaproteobacteria		outgroup	1830138
Haemophilus somnus 129PT	no	Gammaproteobacteria		outgroup	2012878
Hahella chejuensis KCTC 2396	no	Gammaproteobacteria		outgroup	7215267
Haloarcula marismortui ATCC 43049	no	Euryarchaeota		outgroup	3985628
Halobacterium sp. NRC-1	no	Euryarchaeota		outgroup	2571010
Haloquadratum walsbyi DSM 16790	no	Euryarchaeota		outgroup	3179361
Helicobacter acinonychis str. Sheeba	no	Epsilonproteobacteria		outgroup	1557588
Helicobacter hepaticus ATCC 51449	no	Epsilonproteobacteria		outgroup	1799146
Helicobacter pylori 26695	no	Epsilonproteobacteria		outgroup	1667867
Helicobacter pylori HPAG1	no	Epsilonproteobacteria		outgroup	1605736
Helicobacter pylori J99	no	Epsilonproteobacteria		outgroup	1643831

Hyphomonas neptunium ATCC 15444	no	Alphaproteobacteria		outgroup	3705021
Idiomarina loihiensis L2TR	no	Gammaproteobacteria		outgroup	2839318
Jannaschia sp. CCS1	no	Alphaproteobacteria		outgroup	4404049
Lactobacillus acidophilus NCFM	no	Firmicutes		outgroup	1993564
Lactobacillus brevis ATCC 367	no	Firmicutes		outgroup	2340228
Lactobacillus casei ATCC 334	no	Firmicutes		outgroup	2924325
Lactobacillus delbrueckii subsp. bulgaricus ATCC 11842	no	Firmicutes		outgroup	1864998
Lactobacillus delbrueckii subsp. bulgaricus ATCC BAA-365	no	Firmicutes		outgroup	1856951
Lactobacillus gasseri ATCC 33323	no	Firmicutes		outgroup	1894360
Lactobacillus johnsonii NCC 533	no	Firmicutes		outgroup	1992676
Lactobacillus plantarum WCFS1	no	Firmicutes		outgroup	3348625
Lactobacillus sakei subsp. sakei 23K	no	Firmicutes		outgroup	1884661
Lactobacillus salivarius subsp. salivarius UCC118	no	Firmicutes		outgroup	2133977
Lactococcus lactis subsp. cremoris SK11	no	Firmicutes		outgroup	2598348
Lactococcus lactis subsp. lactis Il1403	no	Firmicutes		outgroup	2365589
Leifsonia xyli subsp. xyli str. CTCB07	no	Actinobacteria		outgroup	2584158
Lentisphaera araneosa	no	Lentisphaerae		outgroup	6023180
Leptospira borgpetersenii serovar Hardjo-bovis JB197	no	Spirochaetes		outgroup	3876235
Leptospira borgpetersenii serovar Hardjo-bovis L550	no	Spirochaetes		outgroup	3931782
Leptospira interrogans serovar Copenhageni str. Fiocruz L1-130	no	Spirochaetes		outgroup	4627366
Leptospira interrogans serovar Lai str. 56601	no	Spirochaetes		outgroup	4691184
Leuconostoc mesenteroides subsp. mesenteroides ATCC 8293	no	Firmicutes		outgroup	2075763
Listeria innocua Clip11262	no	Firmicutes		outgroup	3011208
Listeria welshimeri serovar 6b str. SLCC5334	no	Firmicutes		outgroup	2814130
Magnetococcus sp. MC-1	no	unknown Proteobacteria		outgroup	4719581
Magnetospirillum magneticum AMB-1	no	Alphaproteobacteria		outgroup	4967148
Mannheimia succiniciproducens MBEL55E	no	Gammaproteobacteria		outgroup	2314078
Maricaulis maris MCS10	no	Alphaproteobacteria		outgroup	3368780
Marinobacter aquaeolei VT8	no	Gammaproteobacteria		outgroup	4779762
Mesoplasma florum L1	no	Firmicutes		outgroup	793224
Methanocaldococcus jannaschii DSM 2661	no	Euryarchaeota		outgroup	1739927
Methanococcoides burtonii DSM 6242	no	Euryarchaeota		outgroup	2575032
Methanococcus maripaludis S2	no	Euryarchaeota		outgroup	1661137
Methanopyrus kandleri AV19	no	Euryarchaeota		outgroup	1694969
Methanosaeta thermophila PT	no	Euryarchaeota		outgroup	1879471
Methanosarcina acetivorans C2A	no	Euryarchaeota		outgroup	5751492
Methanosarcina barkeri str. Fusaro	no	Euryarchaeota		outgroup	4873766
Methanosarcina mazei Go1	no	Euryarchaeota		outgroup	4096345
Methanosphaera stadtmanae DSM 3091	no	Euryarchaeota		outgroup	1767403
Methanospirillum hungatei JF-1	no	Euryarchaeota		outgroup	3544738
Methanothermobacter thermautotrophicus str. Delta H	no	Euryarchaeota		outgroup	1751377
Methylobacillus flagellatus KT	no	Betaproteobacteria		outgroup	2971517
Methylococcus capsulatus str. Bath	no	Gammaproteobacteria		outgroup	3304561
Moorella thermoacetica ATCC 39073	no	Firmicutes		outgroup	2628784
Mycobacterium smegmatis str. MC2 155	no	Actinobacteria		outgroup	6988209
Mycobacterium sp. KMS	no	Actinobacteria		outgroup	6256079
Mycobacterium sp. MCS	no	Actinobacteria		outgroup	5920523
Mycobacterium ulcerans Agy99	no	Actinobacteria		outgroup	5631606

Mycobacterium vanbaalenii PYR-1	no	Actinobacteria		outgroup	6491865
Mycoplasma capricolum subsp. capricolum ATCC 27343	no	Firmicutes		outgroup	1010023
Mycoplasma gallisepticum R	no	Firmicutes		outgroup	996422
Mycoplasma genitalium G37	no	Firmicutes		outgroup	580074
Mycoplasma hyopneumoniae 232	no	Firmicutes		outgroup	892758
Mycoplasma hyopneumoniae 7448	no	Firmicutes		outgroup	920079
Mycoplasma hyopneumoniae J	no	Firmicutes		outgroup	897405
Mycoplasma mobile 163K	no	Firmicutes		outgroup	777079
Mycoplasma mycoides subsp. mycoides SC str. PG1	no	Firmicutes		outgroup	1211703
Mycoplasma pneumoniae M129	no	Firmicutes		outgroup	816394
Mycoplasma pulmonis UAB CTIP	no	Firmicutes		outgroup	963879
Mycoplasma synoviae 53	no	Firmicutes		outgroup	799476
Myxococcus xanthus DK 1622	no	Deltaproteobacteria		outgroup	9139763
Nanoarchaeum equitans Kin4-M	no	Nanoarchaeota		outgroup	490885
Natronomonas pharaonis DSM 2160	no	Euryarchaeota		outgroup	2749696
Nitrobacter hamburgensis X14	no	Alphaproteobacteria		outgroup	5011522
Nitrobacter winogradskyi Nb-255	no	Alphaproteobacteria		outgroup	3402093
Nitrosococcus oceani ATCC 19707	no	Gammaproteobacteria		outgroup	3522111
Nitrosomonas europaea ATCC 19718	no	Betaproteobacteria		outgroup	2812094
Nitrosomonas eutropha C91	no	Betaproteobacteria		outgroup	2781824
Nitrosospira multiformis ATCC 25196	no	Betaproteobacteria		outgroup	3234309
Nocardioides sp. JS614	no	Actinobacteria		outgroup	5293685
Nostoc sp. PCC 7120	no	Cyanobacteria		outgroup	7211789
Novosphingobium aromaticivorans DSM 12444	no	Alphaproteobacteria		outgroup	3561584
Oceanobacillus iheyensis HTE831	no	Firmicutes		outgroup	3630528
Oenococcus oeni PSU-1	no	Firmicutes		outgroup	1780517
Paracoccus denitrificans PD1222	no	Alphaproteobacteria		outgroup	5236194
Pediococcus pentosaceus ATCC 25745	no	Firmicutes		outgroup	1832387
Pelobacter carbinolicus DSM 2380	no	Deltaproteobacteria		outgroup	3665893
Pelobacter propionicus DSM 2379	no	Deltaproteobacteria		outgroup	4241119
Pelodictyon luteolum DSM 273	no	Chlorobi		outgroup	2364842
Photobacterium profundum SS9	no	Gammaproteobacteria		outgroup	6403280
Photorhabdus asymbiotica	no	Gammaproteobacteria		outgroup	5094138
Photorhabdus luminescens subsp. laumondii TTO1	no	Gammaproteobacteria		outgroup	5688987
Picrophilus torridus DSM 9790	no	Euryarchaeota		outgroup	1545895
Polaromonas sp. Js666	no	Betaproteobacteria		outgroup	5898676
Prochlorococcus marinus str. MIT 9312	no	Cyanobacteria		outgroup	1709204
Prochlorococcus marinus str. NATL2A	no	Cyanobacteria		outgroup	1842899
Prochlorococcus marinus subsp. marinus str. CCMP1375	no	Cyanobacteria		outgroup	1751080
Prochlorococcus marinus subsp. pastoris str. CCMP1986	no	Cyanobacteria		outgroup	1657990
Propionibacterium acnes KPA171202	no	Actinobacteria		outgroup	2560265
Pseudoalteromonas atlantica T6c	no	Gammaproteobacteria		outgroup	5187005
Pseudoalteromonas haloplanktis TAC125	no	Gammaproteobacteria		outgroup	3850272
Pseudomonas entomophila L48	no	Gammaproteobacteria		outgroup	5888780
Pseudomonas fluorescens Pf-5	no	Gammaproteobacteria		outgroup	7074893
Pseudomonas fluorescens Pfo-1	no	Gammaproteobacteria		outgroup	6438405
Pseudomonas putida KT2440	no	Gammaproteobacteria		outgroup	6181863
Pseudomonas syringae pv. phaseolicola 1448A	no	Gammaproteobacteria		outgroup	6112448
Pseudomonas syringae pv. syringae B728a	no	Gammaproteobacteria		outgroup	6093698

Pseudomonas syringae pv. tomato str. DC3000	no	Gammaproteobacteria		outgroup	6538260
Psychrobacter arcticus 273-4	no	Gammaproteobacteria		outgroup	2650701
Psychrobacter cryohalolentis K5	no	Gammaproteobacteria		outgroup	3059876
Psychromonas ingrahamii 37	no	Gammaproteobacteria		outgroup	4559598
Pyrobaculum aerophilum str. IM2	no	Crenarchaeota		outgroup	2222430
Pyrobaculum islandicum DSM 4184	no	Crenarchaeota		outgroup	1826402
Pyrococcus abyssi GE5	no	Euryarchaeota		outgroup	1768562
Pyrococcus furiosus DSM 3638	no	Euryarchaeota		outgroup	1908256
Pyrococcus horikoshii OT3	no	Euryarchaeota		outgroup	1738505
Ralstonia eutropha H16	no	Betaproteobacteria		outgroup	6964522
Ralstonia eutropha JMP134	no	Betaproteobacteria		outgroup	7255290
Ralstonia metallidurans CH34	no	Betaproteobacteria		outgroup	6913352
Ralstonia solanacearum GMI1000	no	Betaproteobacteria		outgroup	5810922
Rhodococcus sp. RHA1	no	Actinobacteria		outgroup	9702737
Rhodoferax ferrireducens T118	no	Betaproteobacteria		outgroup	4969784
Rhodopirellula baltica SH 1	no	Planctomycetes		outgroup	7145576
Rhodopseudomonas palustris BisA53	no	Alphaproteobacteria		outgroup	5505494
Rhodopseudomonas palustris BisB18	no	Alphaproteobacteria		outgroup	5513844
Rhodopseudomonas palustris BisB5	no	Alphaproteobacteria		outgroup	4892717
Rhodopseudomonas palustris CGA009	no	Alphaproteobacteria		outgroup	5459213
Rhodopseudomonas palustris HaA2	no	Alphaproteobacteria		outgroup	5331656
Rhodospirillum rubrum ATCC 11170	no	Alphaproteobacteria		outgroup	4406557
Roseobacter denitrificans OCh 114	no	Alphaproteobacteria		outgroup	4331234
Rubrobacter xylanophilus DSM 9941	no	Actinobacteria		outgroup	3225748
Saccharophagus degradans 2-40	no	Gammaproteobacteria		outgroup	5057531
Salinibacter ruber DSM 13855	no	Bacteroidetes		outgroup	3587328
Shewanella amazonensis SB2B	no	Gammaproteobacteria		outgroup	4306142
Shewanella denitrificans OS217	no	Gammaproteobacteria		outgroup	4545906
Shewanella frigidimarina NCIMB 400	no	Gammaproteobacteria		outgroup	4845257
Shewanella oneidensis MR-1	no	Gammaproteobacteria		outgroup	5131416
Shewanella sp. ANA-3	no	Gammaproteobacteria		outgroup	5251146
Shewanella sp. MR-4	no	Gammaproteobacteria		outgroup	4706287
Shewanella sp. MR-7	no	Gammaproteobacteria		outgroup	4799109
Shewanella sp. W3-18-1	no	Gammaproteobacteria		outgroup	4708380
Silicibacter pomeroyi DSS-3	no	Alphaproteobacteria		outgroup	4601053
Silicibacter sp. TM1040	no	Alphaproteobacteria		outgroup	4153699
Solibacter usitatus Ellin6076	no	Acidobacteria		outgroup	9965640
Sphingopyxis alaskensis RB2256	no	Alphaproteobacteria		outgroup	3373713
Staphylococcus aureus RF122	no	Firmicutes		outgroup	2742531
Staphylococcus aureus subsp. aureus COL	no	Firmicutes		outgroup	2813862
Staphylococcus aureus subsp. aureus MRSA252	no	Firmicutes		outgroup	2902619
Staphylococcus aureus subsp. aureus MSSA476	no	Firmicutes		outgroup	2820454
Staphylococcus aureus subsp. aureus Mu50	no	Firmicutes		outgroup	2903636
Staphylococcus aureus subsp. aureus Mw2	no	Firmicutes		outgroup	2820462
Staphylococcus aureus subsp. aureus N315	no	Firmicutes		outgroup	2839469
Staphylococcus aureus subsp. aureus NCTC 8325	no	Firmicutes		outgroup	2821361
Staphylococcus aureus subsp. aureus USA300	no	Firmicutes		outgroup	2872769
Staphylococcus epidermidis ATCC 12228	no	Firmicutes		outgroup	2564615
Staphylococcus epidermidis RP62A	no	Firmicutes		outgroup	2643840

Staphylococcus haemolyticus JCSC1435	no	Firmicutes		outgroup	2685015
Staphylococcus saprophyticus subsp. saprophyticus ATCC 15305	no	Firmicutes		outgroup	2577899
Streptococcus mutans UA159	no	Firmicutes		outgroup	2030921
Streptococcus pneumoniae D39	no	Firmicutes		outgroup	2046115
Streptococcus pneumoniae R6	no	Firmicutes		outgroup	2038615
Streptococcus pneumoniae TIGR4	no	Firmicutes		outgroup	2160842
Streptococcus thermophilus CNRZ1066	no	Firmicutes		outgroup	1796226
Streptococcus thermophilus LMD-9	no	Firmicutes		outgroup	1864178
Streptococcus thermophilus LMG 18311	no	Firmicutes		outgroup	1796846
Streptomyces avermitilis MA-4680	no	Actinobacteria		outgroup	9119895
Streptomyces coelicolor A3(2)	no	Actinobacteria		outgroup	9054847
Sulfolobus acidocaldarius DSM 639	no	Crenarchaeota		outgroup	2225959
Sulfolobus solfataricus P2	no	Crenarchaeota		outgroup	2992245
Sulfolobus tokodaii str. 7	no	Crenarchaeota		outgroup	2694756
Symbiobacterium thermophilum IAM 14863	no	Firmicutes		outgroup	3566135
Synechococcus elongatus PCC 6301	no	Cyanobacteria		outgroup	2696255
Synechococcus elongatus PCC 7942	no	Cyanobacteria		outgroup	2742269
Synechococcus sp. CC9311	no	Cyanobacteria		outgroup	2606748
Synechococcus sp. CC9605	no	Cyanobacteria		outgroup	2510659
Synechococcus sp. CC9902	no	Cyanobacteria		outgroup	2234828
Synechococcus sp. JA-2-3B'a(2-13)	no	Cyanobacteria		outgroup	3046682
Synechococcus sp. JA-3-3Ab	no	Cyanobacteria		outgroup	2932766
Synechococcus sp. WH 8102	no	Cyanobacteria		outgroup	2434428
Synechocystis sp. PCC 6803	no	Cyanobacteria		outgroup	3949364
Syntrophobacter fumaroxidans MPOB	no	Deltaproteobacteria		outgroup	4990251
Syntrophomonas wolfei subsp. wolfei str. Goettingen	no	Firmicutes		outgroup	2936195
Syntrophus aciditrophicus	no	Deltaproteobacteria		outgroup	3179300
Thermoanaerobacter tengcongensis MB4	no	Firmicutes		outgroup	2689445
Thermobifida fusca YX	no	Actinobacteria		outgroup	3642249
Thermococcus kodakarensis KOD1	no	Euryarchaeota		outgroup	2088737
Thermofilum pendens Hrk 5	no	Crenarchaeota		outgroup	1813393
Thermoplasma acidophilum DSM 1728	no	Euryarchaeota		outgroup	1564906
Thermoplasma volcanium GSS1	no	Euryarchaeota		outgroup	1584804
Thermosynechococcus elongatus BP-1	no	Cyanobacteria		outgroup	2593857
Thermotoga maritima MSB8	no	Thermotogae		outgroup	1860725
Thermus thermophilus HB27	no	Deinococcus-Thermus		outgroup	1894877
Thermus thermophilus HB8	no	Deinococcus-Thermus		outgroup	2116056
Thiobacillus denitrificans ATCC 25259	no	Betaproteobacteria		outgroup	2909809
Thiomicrospira crunogena XCL-2	no	Gammaproteobacteria		outgroup	2427734
Thiomicrospira denitrificans ATCC 33889	no	Gammaproteobacteria		outgroup	2201561
Treponema denticola ATCC 35405	no	Spirochaetes		outgroup	2843201
Trichodesmium erythraeum IMS101	no	Cyanobacteria		outgroup	7750108
Verrucomicrobium spinosum	no	Verrucomicrobia		outgroup	8220857
Vibrio fischeri ES114	no	Gammaproteobacteria		outgroup	4284050
Vibrio parahaemolyticus RIMD 2210633	no	Gammaproteobacteria		outgroup	5165770
Vibrio vulnificus CMCP6	no	Gammaproteobacteria		outgroup	5126797
Vibrio vulnificus YJ016	no	Gammaproteobacteria		outgroup	5260086
Victivallis vadensis	no	Lentisphaerae		outgroup	4577257
Wolinella succinogenes DSM 1740	no	Epsilonproteobacteria		outgroup	2110355
Xanthomonas axonopodis pv. citri str. 306	no	Gammaproteobacteria		outgroup	5274174
Xanthomonas campestris pv. campestris str. 8004	no	Gammaproteobacteria		outgroup	5148708

Xanthomonas campestris pv. campestris str. ATCC 33913	no	Gammaproteobacteria		outgroup	5076188
Xanthomonas campestris pv. vesicatoria str. 85-10	no	Gammaproteobacteria		outgroup	5178466
Xanthomonas oryzae pv. oryzae KACC10331	no	Gammaproteobacteria		outgroup	4941439
Xanthomonas oryzae pv. oryzae MAFF 311018	no	Gammaproteobacteria		outgroup	4940217
Xylella fastidiosa 9a5c	no	Gammaproteobacteria		outgroup	2731750
Xylella fastidiosa Temecula1	no	Gammaproteobacteria		outgroup	2521148
Zymomonas mobilis subsp. mobilis ZM4	no	Alphaproteobacteria		outgroup	2056416

Table S8: Taxonomic profiles of chlamydial protein clusters.
* *Cc*, *Chlamydiaceae*; *Pc*, *Parachlamydiaceae*; OM, outer membrane clusters; CP, all clusters except the outer membrane clusters.

	Cc* OM	***Cc* CP***	***Pc* OM***	***Pc* CP***
Acidobacteria	24	523	26	864
Actinobacteria	19	586	14	981
Aquificae	9	428	13	591
Bacteroidetes	17	584	22	992
Chlorobi	15	470	22	758
Chloroflexi	7	341	6	502
Crenarchaeota	8	315	6	535
Cyanobacteria	24	559	24	945
Deinococcus-Thermus	18	492	17	752
Euryarchaeota	21	446	14	801
Firmicutes	33	641	21	1079
Fusobacteria	13	423	17	596
Lentisphaerae	17	527	23	808
Planctomycetes	24	589	25	982
Alphaproteobacteria	38	624	34	1011
Betaproteobacteria	28	603	30	1056
Gammaproteobacteria	37	653	34	1165
Deltaproteobacteria	33	647	36	1116
Epsilonproteobacteria	26	500	23	743
**unclassified *Proteobacteria***	11	483	17	716
Spirochaetes	21	573	25	880
Thermotogae	12	408	15	557
Verrucomicrobia	21	499	28	790

Table S9: Distribution of orthologous proteins based on lifestyle.
***** *Cc*, *Chlamydiaceae*; *Pc*, *Parachlamydiaceae*; *Ec*, *E. coli*; OM, outer membrane clusters; CP, all clusters except the outer membrane clusters.

	Cc* OM	***Pc* OM***	***Ec* OM***
**obligate intracellular outgroup organism**	29	28	85
**facultative intracellular outgroup organism**	33	33	123
**free-living outgroup organism**	47	41	125

Table S10: Cluster analysis based on distribution of obligate intracellular human pathogen
* OM, outer membrane clusters; CP, all clusters except the outer membrane clusters.

	Cc* OM	***Pc* OM***	***Ec* OM***
**obligate intracellular human pathogen**	21	20	36
**no obligate intracellular human pathogen**	26	21	89

Table S11: Representation of the PVC superphylum in outer membrane clusters.
***** *Cc*, *Chlamydiaceae*; *Pc*, *Parachlamydiaceae*; *Ec*, *E. coli*; OM, outer membrane clusters; CP, all clusters except the outer membrane clusters.

	Cc* OM	***Pc* OM***	***Ec* OM***
Verrucomicrobia	21	28	25
Lentisphaerae	17	23	28
Planctomycetes	24	25	33
**No PVC**	15	8	133
